# Reduced neural selectivity for mental states in deaf children with delayed exposure to sign language

**DOI:** 10.1038/s41467-020-17004-y

**Published:** 2020-06-26

**Authors:** Hilary Richardson, Jorie Koster-Hale, Naomi Caselli, Rachel Magid, Rachel Benedict, Halie Olson, Jennie Pyers, Rebecca Saxe

**Affiliations:** 1 Department of Brain and Cognitive Sciences, Massachusetts Institute of Technology, 43 Vassar Street, 46-4021, Cambridge, MA 02139 USA; 2 Laboratories of Cognitive Neuroscience, Division of Developmental Medicine, Boston Children’s Hospital, 1 Autumn Street, Rm. 527, Boston, MA 02215 USA; 3 Department of Pediatrics, Harvard Medical School, 1 Autumn Street, Rm. 527, Boston, MA 02215 USA; 4 Wheelock College of Education and Human Development, Boston University, 621 Commonwealth Avenue, Rm. 218, Boston, MA 02215 USA; 5 Department of Psychology, Wellesley College, 106 Central Street, Wellesley, MA 02481 USA

**Keywords:** Cognitive neuroscience, Psychology

## Abstract

Language provides a rich source of information about other people’s thoughts and feelings. Consequently, delayed access to language may influence conceptual development in Theory of Mind (ToM). We use functional magnetic resonance imaging and behavioral tasks to study ToM development in child (*n* = 33, 4–12 years old) and adult (*n* = 36) fluent signers of American Sign Language (ASL), and characterize neural ToM responses during ASL and movie-viewing tasks. Participants include deaf children whose first exposure to ASL was delayed up to 7 years (*n* = 12). Neural responses to ToM stories (specifically, selectivity of the right temporo-parietal junction) in these children resembles responses previously observed in young children, who have similar linguistic experience, rather than those in age-matched native-signing children, who have similar biological maturation. Early linguistic experience may facilitate ToM development, via the development of a selective brain region for ToM.

## Abstract (web summary)

Early linguistic experience directly facilitates social development in childhood. Here, the authors reveal that children with delayed access to language show delayed development of selective responses in cortical regions involved in thinking about others’ thoughts.

## Introduction

The human ability to reason about the mental states of others is described as having a “Theory of Mind” (ToM): a rich, structured theory that explains observable behaviors in terms of unobservable beliefs, desires, and emotions. Like many cognitive capacities, ToM reasoning develops dramatically during early childhood. While evidence suggests that ToM development is domain-specific^1,2^, environmental and experiential factors, like a child’s linguistic experiences, clearly contribute to social cognitive change^3–7^. Does linguistic experience directly impact ToM development, or does it mainly enable performance on ToM tasks? Here, we leverage recent discoveries about the development of brain regions that support ToM reasoning in order to test for a direct influence of linguistic experience on ToM development.

Most studies on ToM use the false-belief task, in which participants predict the action of a character who has a false belief (e.g., Sally thinks that the cookie is in the drawer, but it is actually in the desk; where will Sally look for her cookie first?). Exposure to mental-state vocabulary (e.g., think, know) and syntactic complement structures (e.g., Sally thinks that…) predicts performance on false-belief tasks^8,9^, even when controlling for current language abilities^8^. Nevertheless, it remains unclear whether improved performance on false-belief tasks reflects more sophisticated ToM representations or an improved ability to meet linguistic task demands.

Traditional false-belief tasks require children to comprehend linguistically sophisticated narratives and questions. Consequently, performance on these tasks reflects children’s ability to follow, understand, and remember a linguistic narrative and question, and (typically) to form, select, and produce a linguistic response^10^. Linguistic abilities correlate with children’s performance on traditional false-belief tasks^11,12^, and children often perform better on conceptually analogous non- or minimally linguistic false belief tasks^13^, suggesting that linguistic abilities may be a rate-limiting factor for performance on traditional ToM tasks.

One possibility is therefore that linguistic experience primarily affects performance on ToM tasks indirectly, via a direct effect on the linguistic abilities children need for traditional ToM tasks. This possibility is particularly salient given controversial evidence that toddlers and infants pass nonlinguistic false-belief tasks^14^ (though see ref. ^15^). Some have claimed that infants have the concepts and representational capacities to reason about others’ beliefs, and that subsequent improvement on traditional ToM tasks reflects development of language and executive functions that enable children to meet task demands^16^. However, the representational capacities required to pass nonlinguistic false-belief tasks remain debated^17,18^.

On the other hand, conversational experience may play a direct role in the construction of ToM concepts and representations^19–21^. Children could learn to differentiate mental-state concepts (e.g., think vs. know) from the way adults use mental-state verbs in conversation^8,22^. Conversations also provide experience with linguistic structures that could be particularly important for ToM representations used during false-belief tasks, like syntactic complement structures^8,9,23,24^. Even utterances that do not contain mental-state verbs or syntactic complement structures (e.g., where are my keys?) provide evidence about beliefs and desires, and the link between those mental states and observable behavior^25^. Experience with linguistically rich conversations could thus directly promote development of a more precise and accurate understanding of other minds.

The role of language in facilitating ToM development is especially relevant for understanding ToM development in children who are d/Deaf^26^. (Note that the capitalized word “Deaf” refers to the cultural and linguistic minority group, and the lower-case “deaf” refers to the audiological status^27^. We use “deaf” because we were often unable to distinguish between the two (e.g., in young children)). Many deaf or hard-of-hearing children are at risk of not learning any language in early childhood because they have limited auditory access to spoken language and their families do not know sign language at the time of birth^28^. Deaf children with delayed exposure to sign language show delays in ToM relative to hearing children and to deaf children exposed to sign language from infancy^29–37^. Interestingly, this delay appears not to be fully explained by the linguistic demands of ToM tasks. Delayed exposure to sign language affects performance on linguistic and minimally linguistic false-belief tasks^29–36^, and even on nonlinguistic anticipatory looking paradigms^37^. Also, the effects on ToM are correlated not just with the child’s language abilities, but specifically with the richness of maternal mental-state language^38^.

In sum, it remains an open question whether early linguistic experiences influence ToM mainly indirectly, by enhancing children’s linguistic abilities to succeed on ToM tasks, or also directly, by supporting children’s ToM development. Neuroimaging evidence can provide insight into this question because alternative hypotheses about the role of linguistic experience in ToM development make distinct predictions for the response profiles we should observe in the brains of children who experience delayed access to language.

Human adults and children recruit a specific cortical network when reasoning about the minds of others, including bilateral temporoparietal junction (TPJ), precuneus, and medial prefrontal cortex^39,40^. While many brain regions are recruited to process narratives and movies, these brain regions (the “ToM network”) show high hemodynamic responses to linguistic narratives^41^, nonlinguistic cartoons^42,43^, and movies^44^ that evoke considerations of characters’ mental states, relative to non-mentalistic control stimuli. As children get older, the ToM network becomes increasingly functionally distinct^45,46^ and structurally connected (i.e., via increased myelination^47^). Also, the right TPJ becomes more selective for mental states^48,49^. By adulthood, the RTPJ preferentially responds when people think about beliefs and desires, relative to meta-representations like photographs or maps^41^, internal states like physical pain^44,50^ or bodily sensations^51^, and social information like a person’s appearance or place of origin^52,53^.

In many cortical regions, increasing selectivity during childhood occurs by the suppression of responses to non-preferred stimuli. For example, selectivity of the fusiform face area develops through the suppression of responses to non-face objects^54,55^. Selectivity of the visual word form area develops through the suppression of responses to faces^54^. Similarly, selectivity of the RTPJ develops through the suppression of responses to other (non-mentalistic) social information^48,49^. While the development of selective brain regions for perception of faces or words appears to depend on extensive experience with those visual categories^56–58^, there is currently no evidence to test whether development of selective responses in RTPJ is influenced by environmental input, or which aspects of the environment are most important.

If linguistic experience directly influences development of domain-specific ToM concepts, then delayed access to language may affect the development of selective responses in RTPJ. That is, instead of resembling responses in chronologically age-matched children, who have the same amount of biological maturation, the RTPJ response in delayed signers might most resemble responses in younger typically developing children, who have the same amount of linguistic experience. We predicted that the response in RTPJ in native signers would be similar to previously observed ToM-selective responses in age-matched hearing children, and test whether RTPJ responses are less selective as a function of delayed access to language. These specific neural predictions provide a complementary way to investigate the role of linguistic experience in ToM development—which is not only theoretically significant, but also provides important information for parents, who must make difficult choices about how their child learns language.

We measure ToM behaviorally and neurally in 33 fluent-signing children (4–12 years old; *n* = 33) and adults (*n* = 36), including native (NS) and delayed signers (DS). All participants were exposed to American Sign Language (ASL) and had fluent, age-appropriate comprehension of ASL morphology and syntax at the time of the study (Supplementary Table 1). Because fluent-signing children with delayed exposure to ASL but no cochlear implant are rare, intense recruitment efforts over 4 years yielded a small sample of DS children (*n* = 12), with age of first exposure to ASL, a proxy for linguistic experience, ranging from 0.25–7 years. In the primary fMRI experiment, a narrator tells stories in ASL describing physical events (Physical), characters’ appearance and relationships (Social), and characters’ beliefs, desires, and emotions (Mental). We use pre-registered analysis protocols to measure response selectivity in RTPJ to the Mental stories as a function of delayed exposure to language (https://osf.io/mhgp8). However, because this dataset is exceedingly difficult to collect, and as such, is precious, we additionally conduct pre-registered analyses to test effects of age of ASL onset on other aspects of cortical development (ToM: https://osf.io/mhgp8; Language: https://osf.io/7y263).

We report evidence that delayed exposure to sign language delays the development of selective responses for mental states in the RTPJ. Early linguistic experience may facilitate social development via the development of a brain region that is selective for reasoning about minds.

## Results

### Behavioral results

Child participants completed a measure of receptive ASL (ASL-RST^59^). Receptive ASL proficiency increased with age (Pearson’s correlation: *r*(29) = 0.54, 95% CI = [0.23, 0.75], *P* = 0.002), but did not vary as a function of age of ASL onset (M(SE) proportion correct in delayed signers: 0.79 (0.02); native signers: 0.70 (0.04); effect of age of ASL onset: *b* = −0.09, *t* = −0.55, 95% CI = [−0.44, 0.25], *P* = 0.59, effect of age: *b* = 0.57, *t* = 3.4, 95% CI = [0.23, 0.92], *P* = 0.002).

**Fig. 1:**
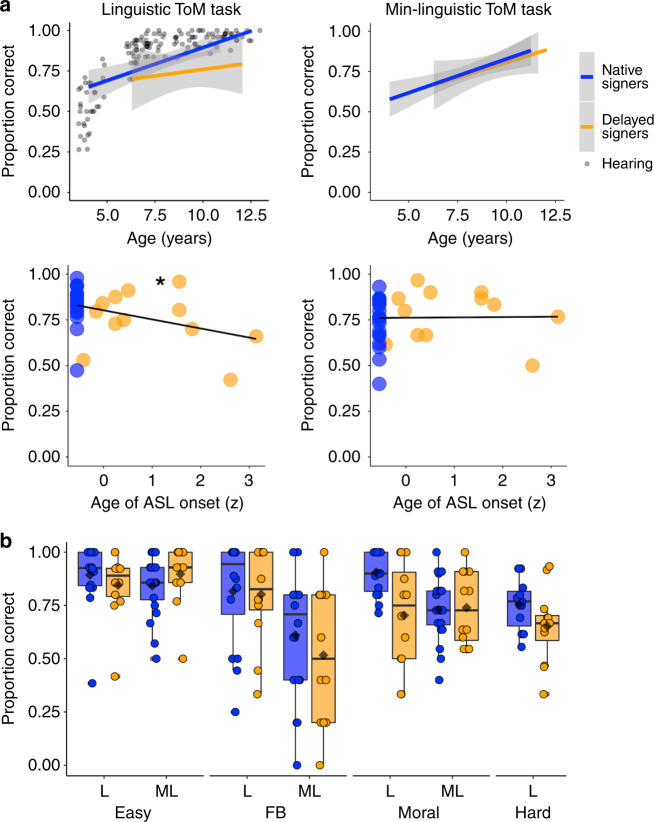
Theory of mind behavior. **a** Proportion correct on linguistic (L (ASL); left) and minimally linguistic (ML; right) ToM tasks (*y*-axis) by age (top row; *x*-axis) or by *Z*-scored age of ASL onset (middle row; *x*-axis). *Z* scores are used for de-identification of data. Native signers (*n* = 16, L task; *n* = 21, ML task) are shown in blue; delayed signers (*n* = 12 (both tasks)) are shown in orange. For the linguistic task, gray dots show 3–12 year-old hearing participants who completed an analogous task in English in other studies (*n* = 137). Gray shading (top row) shows the 95% confidence level interval for a linear regression predicting the effect of age on ToM performance, per ASL signing group. Asterisk (middle left) indicates a significant effect of age of ASL onset on linguistic ToM performance (*b* = −0.54, *t* = −3.4, 95% CI = [−0.86, −0.21], *P* = 0.002; linear regression controlling for age). **b** Standard boxplots of proportion correct on linguistic and minimally linguistic ToM tasks for native (blue; *n* = 16, L task; *n* = 21, ML task) and delayed (orange, *n* = 12, both tasks) signers (*y*-axis) by question category (*x*-axis; FB refers to false-belief items). Center line indicates median, box reflects interquartile range, whiskers show first quartile/third quartile −/+ 1.5*IQR, means are shown with black diamonds, and individual data points are shown with blue (NS) and orange (DS) circles. Source data are provided as a Source Data file and at https://osf.io/kyu3f/.

We measured ToM reasoning using linguistic (ToM_L_) and minimally linguistic (ToM_ML_) tasks. Across all children, performance on both tasks positively correlated with age (Kendall tau correlation, given non-normal distribution of ToM scores: ToM_L_: *r*(26) = 0.40, 95% CI = [0.03, 0.67], *P* = 0.04; ToM_ML_: *r*(31) = 0.63, 95% CI = [0.36, 0.80], *P* = 9.3 × 10^−5^; Fig. 1a top row). Due to the small number of child participants, we include a large sample of age-matched neurotypical hearing children in figures. These participants are described within the legends of the figures that include them; statistical tests include delayed and native signers only.

Performance on the ToM_L_ and ToM_ML_ tasks was positively correlated (Kendall tau correlation: *r*(26) = 0.63, 95% CI = [0.34, 0.81], *P* = 0.0003), even when controlling for age and ASL proficiency (ToM_ML_: *b* = 0.71, *t* = 3.0, 95% CI = [0.21, 1.2], *P* = 0.007). ASL proficiency was marginally positively correlated with ToM_L_ task performance (*r*(26) = 0.32, 95% CI = [−0.06, 0.62], *P* = 0.10), and significantly positively correlated with ToM_ML_ task performance (*r*(29) = 0.63, 95% CI = [0.36, 0.81], *P* = 0.0001; Kendall tau correlations).

Critically, we tested if performance on either ToM task varied as a function of age of ASL onset. Children who experienced a longer delay before exposure to ASL performed worse on the ToM_L_ task (M(SE) proportion correct in delayed signers: 0.75(0.04); native signers: 0.83(0.03); effect of age of ASL onset: *b* = −0.54, *t* = −3.4, 95% CI = [−0.86, −0.21], *P* = 0.002; age: *b* = 0.56, *t* = 3.5, 95% CI = [0.23, 0.88], *P* = 0.002; Fig. 1a). This effect remained significant when controlling for ASL proficiency (age of ASL onset: *b* = −0.52, *t* = −3.2, 95% CI = [−0.86, −0.19], *P* = 0.004; age: *b* = 0.51, *t* = 2.8, 95% CI = [0.14,.88], *P* = 0.009; ASL proficiency: *b* = 0.10, *t* = 0.59, 95% CI = [−0.25, 0.45], *P* = 0.56) and when including only deaf children in the analysis (i.e., excluding hearing children of deaf adults; age of ASL onset: *b* = −0.54, *t* = −3.2, 95% CI = [−0.90, −0.19], *P* = 0.004). There was no effect of age of ASL onset on control items, suggesting that all children understood and could answer simple questions about the narrative (effect of age of ASL onset: *b* = −0.12, *t* = −0.66, 95% CI = [−.50, 0.26], *P* = 0.52; age: *b* = 0.48, *t* = 2.6, 95% CI = [0.09, 0.86], *P* = 0.02).

There was no effect of age of ASL onset on ToM_ML_ task performance (M(SE) proportion correct in delayed signers: 0.78(0.04); native signers: 0.75(0.03); effect of age of ASL onset: *b* = −0.21, *t* = −1.5, 95% CI = [−0.51, 0.08], *P* = 0.15; age: *b* = 0.70, *t* = 4.8, 95% CI = [0.40, 0.99], *P* = 4.1 × 10^−5^; Fig. 1a). In a regression testing for effects of task format, age of ASL onset, age, and all interactions, children performed worse overall on the ToM_ML_ task (*b* = −0.35, *t* = −2.7, 95% CI = [−0.60, −0.10], *P* = 0.007), performance was reduced in children with longer language delays (*b* = −0.34, *t* = −4.1, 95% CI = [−0.51, 0.18], P = 6.4 × 10^−5^), performance increased with age (*b* = 0.35, *t* = 5.1, 95% CI = [0.22, 0.49], *P* = 6.7 × 10^−7^), and there was a significant age of ASL onset-by-task interaction such that delayed ASL onset had a larger effect on ToM_L_ task performance (*b* = 0.25, *t* = 2.0, 95% CI = [0.006, 0.50], *P* = 0.04).

We additionally tested for differences in non-verbal IQ, spatial working memory, and executive functions as a function of age of ASL onset, controlling for age. Age of ASL onset did not affect spatial working memory (M(SE) span in delayed signers: 4.9(0.23); native signers: 4.7(0.18); effect of age of ASL onset: *b* = −0.27, *t* = −1.4, 95% CI = [−0.66, 0.13], *P* = 0.18) or executive functions (M(SE) reaction time cost (s) for incongruent vs. congruent trials in delayed signers: 0.05(0.02); native signers: 0.03(0.02); effect of age of ASL onset: *b* = −0.08, *t* = −0.36, 95% CI = [−0.53, 0.38], *P* = 0.72). Longer delays of language exposure correlated with lower standardized non-verbal IQ (M(SE) IQ in delayed signers: 111.3(6.8); native signers: 115.7(3.2); effect of age of ASL onset: *b* = −0.38, *t* = −2.3, 95% CI = [−0.73, −0.04], *P* = 0.03). The effect of age of ASL onset on the linguistic ToM task was significant when controlling for standardized non-verbal IQ (age of ASL onset: *b* = −0.33, *t* = −2.2, 95% CI = [−0.64, −0.02], *P* = 0.04; age: *b* = 0.38, *t* = 2.7, 95% CI = [0.09, 0.67], *P* = 0.01; non-verbal IQ: *b* = 0.42, *t* = 2.8, 95% CI = [0.11, 0.73], *P* = 0.009).

### Exploratory analyses of ToM performance by item category

Given reduced performance in delayed signers on the linguistic ToM task, we divided ToM items into four sets of tested concepts: (1) easy (desires, emotions, true beliefs), (2) false beliefs, (3) moral judgments, and, for the linguistic task only, (4) hard (lies, second-order false beliefs, mistaken referents, sarcasm). We plotted proportion correct per category and signing group to visualize which categories contributed to the performance difference by age of ASL onset in the linguistic task (Fig. 1b). In a post hoc linear regression testing for effects of age of ASL onset, age, and item category, age of ASL onset had a negative effect on ToM_L_ performance (*b* = −0.38, *t* = −4.7, 95% CI = [−0.54, −0.22], *P* = 8.0 × 10^−6^), age had a positive effect on performance (*b* = 0.38, *t* = 4.2, 95% CI = [0.20, 0.55], *P* = 4.7 × 10^−5^), and all children performed worse on hard items (*b* = −0.84, *t* = −3.6, 95% CI = [−1.3, −0.38], *P* = 0.0004); there were no significant item category by age of ASL onset interactions (all bs < |.27 | , ts < |1.3 | , 95% CIs = [(−0.70, −0.28), (0.17, 0.58)], ps > 0.2).

### False belief and moral items

Most studies on the relationship between language and ToM use only the false-belief task. Age of ASL onset did not affect performance on false-belief items in the linguistic task (M(SE) proportion correct in delayed signers: 0.80(0.07); native signers: 0.82(0.05); age of ASL onset: *b* = 0.20, *t* = 0.77, 95% CI = [−0.33, 0.72], *P* = 0.45) or minimally linguistic task (M(SE) delayed signers: 0.52(0.09); native signers: 0.61(0.06); age of ASL onset: *b* = −0.53, *t* = −1.7, 95% CI = [−1.2, 0.1], *P* = 0.09). Nonetheless, all children performed better on the linguistic FB items (mixed effect linear regression: task modality: *b* = −1.2, *t* = −5.0, 95% CI = [−1.6, −0.73], *P* = 4.2 × 10^−5^). See Supplementary Note 1 for correlation analyses with receptive ASL.

Moral reasoning is a later-developing component of ToM reasoning. For moral items, age of ASL onset negatively correlated with performance in the ToM_L_ task, controlling for age (M(SE) proportion correct in delayed signers: 0.70(0.07); native signers: 0.91(0.02); age of ASL onset: *b* = −0.50, *t* = −3.0, 95% CI = [−0.84, −0.16], *P* = 0.006); but less so in the ToM_ML_ task (M(SE) delayed signers: 0.74(0.05); native signers: 0.73(0.03); age of ASL onset: *b* = −0.28, *t* = −1.7, 95% CI = [−.61, 0.06], *P* = 0.10). There was a significant age of ASL onset-by-task modality interaction (*b* = 0.33, *t* = 2.1, 95% CI = [0.03, 0.63], *P* = 0.046; Fig. 1b).

### ASL story FMRI task results

**Fig. 2:**
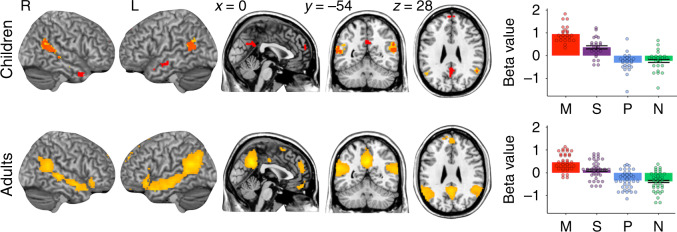
Theory of mind brain regions preferentially respond to ASL stories that describe mental states. Brain images show results of whole-brain random effects analyses for all children (*n* = 24, top row) and adults (*n* = 36, bottom row) for the Mental > Physical stories contrast (one-sample *t* tests). Clusters that survive correction for multiple comparisons using permutation analyses (SnPM; *P* < 0.05) are shown in orange; red clusters show activation where *P* < 0.001, *k* = 10, uncorrected for multiple comparisons (children only); see Table 1 for further information about significant clusters. Bar plots show average beta-value estimates per condition + /− SEM (Mental (red), Social (purple), Physical (blue) stories, and Non-Signs (green) in individually defined functional ToM regions of interest (ROIs; averaged across bilateral temporoparietal junction, precuneus, and dorso-, middle-, and ventromedial prefrontal cortex); dots show individual data points per participant. The adult non-sign condition bar refers to the 8-Non-Sign condition (see Methods). Error bars are omitted for Mental and Physical conditions because these conditions were used for individual ROI definition. See Supplementary Fig. 2 for similar plots in group regions of interest. Source data are provided as a Source Data file and at https://osf.io/kyu3f/.

Whole-brain random effects analyses confirmed that ToM brain regions responded preferentially to stories that described mental states relative to non-mentalistic control stories (Mental > Physical) in children and adults (Fig. 2 and Table 1). There were no voxels that showed significant differences in activation between native-signing and delayed signing participants (Supplementary Fig. 1).

**Table 1:** Theory of mind brain regions preferentially respond to mental-state stories.

Region	Peak coord	Peak *t*	N voxels	*p*_combo_	*w*_combo_
L temporoparietal junction	**[−58, −56, 20]**	**9.01**	**4229**	**0.0004**	**8.82**
	[**−**50, **−**60, 16]	8.50			
	[**−**56, **−**22, **−**8]	7.73			
Precuneus	**[0 −54 34]**	**8.88**	**1535**	**0.0004**	**8.82**
	[−10, **−**50, 34]	7.69			
R temporoparietal junction	**[56, −50, 18]**	**7.98**	**3656**	**0.0004**	**8.82**
	[48, **−**30, −4]	7.66			
	[50, **−**16, **−**14]	7.28			
Medial prefrontal cortex	**[−2, 58, 26]**	**6.03**	**893**	**0.0016**	**7.73**
	[4, 52, 18]	5.81			
	[**−**16, 58, 30]	4.95			

A whole-brain random effects analysis conducted on all participants (*n* = 60 children and adults) confirmed that bilateral temporoparietal junction, medial prefrontal cortex, and precuneus were recruited for the Mental >Physical ASL stories contrast (one-sample *t* test, *P* < 0.05, corrected for multiple comparisons using permutation analyses in SnPM5). Peak coord provides MNI coordinates [*x, y, z*] for the peak *t* statistic(s) of contiguous clusters. Peak t provides the *t*-value per peak t statistic per contiguous cluster. N voxels provide the number of significant voxels per contiguous cluster. *p*_combo_ provides the corrected *P*-value using a combination of cluster size and voxel-wise *t*-value; *w*_combo_ provides the combined cluster and voxel test statistic. Bold rows indicate peak clusters per region.

We tested whether delayed access to linguistic input results in delayed or disrupted functional specialization of ToM brain regions, with a focus on the RTPJ and the dorsomedial prefrontal cortex (DMPFC), given prior evidence that development of these regions relates to behavioral ToM^48,60,61^. In the same session, participants completed the primary story task and a passive movie-viewing task. Not all participants completed both tasks, and some were subsequently excluded due to excessive motion, leaving *n* = 8 delayed signing and *n* = 16 native-signing children in story task analyses, and *n* = 9 delayed signing and *n* = 19 native-signing children in movie-viewing task analyses.

**Fig. 3:**
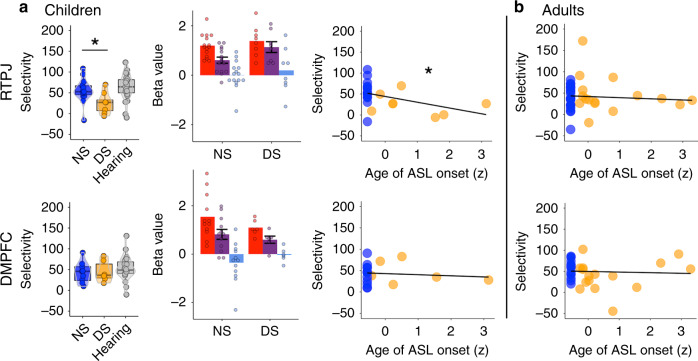
RTPJ & DMPFC responses to ASL story task. **a** Standard boxplots (left) show the distribution of selectivity indices in RTPJ (top row) and DMPFC (bottom row) in native signing (NS; blue; RTPJ: *n* = 15; DMPFC: *n* = 12 (out of 16)), delayed signing (DS; orange; RTPJ: *n* = 8; DMPFC: *n* = 6 (out of 8)), and age-matched neurotypical hearing (gray; *n* = 28 6–10-year old) children, who completed an analogous fMRI story task in English. Center line reflects median, box reflects interquartile range (IQR), whiskers show first quartile/third quartile −/+ 1.5*IQR; dots show individual data points per participant; violin outline shows the distribution of data. Selectivity index was calculated as the average beta estimate to (Mental–Social/Mental–Physical)*100. Asterisk indicates that delayed signers had less selective RTPJ responses than native signers (two-sided non-parametric Mann–Whitney *U* test; W = 97, CI = [4.3, 53.6], *P* = 0.02). Bar plots (middle) show mean beta estimates by condition + /− SEM (Mental (red), Social (purple), Physical (blue)) in individually defined RTPJ (top) and DMPFC (bottom) ROIs in children; dots show individual data points per participant. The mental and physical conditions were used for individual ROI definition and are shown for visualization purposes only (and therefore do not have error bars). Scatterplots (right) show the selectivity index for RTPJ and DMPFC in NS (RTPJ: *n* = 15; DMPFC: *n* = 12) and DS children (RTPJ: *n* = 8; DMPFC: *n* = 6) by *Z*-scored age of ASL onset (*x*-axis). Asterisk indicates a significant effect of age of ASL onset on RTPJ selectivity among children (*b* = −0.47, *t* = −2.3, 95% CI = [−0.90, −0.05], *P* = 0.03; linear regression controlling for age and motion). **b** Scatterplots show the selectivity index for RTPJ and DMPFC in adults (RTPJ (top): *n* = 20 NS (blue), *n* = 16 DS (orange); DMPFC (bottom): *n* = 19 NS, *n* = 16 DS), by *Z*-scored age of ASL onset (*x*-axis). *Z* scores are used on *x*-axes for de-identification of data. Source data are provided as a Source Data file and at https://osf.io/kyu3f/.

Among child participants, there was a significant negative effect of age of ASL onset on response selectivity in the a priori ToM regions of interest (ROIs). Children who had a longer delay before exposure to ASL showed reduced functional selectivity (Table 2, Fig. 3; Supplementary Fig. 2). This effect remained significant when restricting the native-signing group to deaf children (i.e., excluding hearing children of deaf adults; *b* = −0.36, *t* = −2.1, 95% CI = [−0.70, −0.03], *P* = 0.0499). There was no effect of delayed access to language on response selectivity among adults (Table 2). Age-by-ASL onset interactions were non-significant and omitted from regressions. Individual ROIs did not differ in position or size as a function of age of ASL onset (Supplementary Table 2 and Supplementary Note 4), and analyses in group ROIs showed a similar pattern of results (Supplementary Note 5, Supplementary Table 3, and Supplementary Fig. 2).

**Table 2:** Predictors of response selectivity in ToM and language brain regions.

Brain regions	Predictor	Children (*n* = 24)	Adults (*n* = 36)	Full sample (*n* = 60)
ToM (RTPJ, DMPFC)	ASL onset	***b*** = **−0.34,** ***t*** = **−2.1, CI** = **[−0.65, −0.02],** ***P*** = **0.049**	*b* = −0.24, *t* = **−**1.2, CI = [−0.62, 0.14], *P* = 0.23	***b*** = **−0.30,** ***t*** = **−2.2, CI** = **[−0.57, −0.03],** ***P*** = **0.03**
	Age	*b* = 0.31, *t* = 2.0, CI = [0.01, 0.62], *P* = 0.06	NA	*b* = 0.18, *t* = 0.85, CI = [**−**0.23, 0.59], *P* = 0.40
	ROI	*b* = 0.02, *t* = 0.08, CI = [−0.52, 0.57], *P* = 0.94	*b* = −0.24, *t* = **−**1.0, CI = [−0.71, 0.23], *P* = 0.32	*b* = −0.15, *t* = −0.84, CI = [**−**0.51, 0.21], *P* = 0.40
	Motion	*b* = 0.20, *t* = 1.3, CI = [−0.10, 0.51], *P* = 0.21	*b* = 0.22, *t* = 1.2, CI = [−0.16, 0.60], *p* = 0.26	***b*** = **0.29,** ***t*** = **2.1, CI** = **[0.02, 0.55],** ***P*** = **0.04**
Language (11 regions)	ASL onset	b = **−**0.08, *t* = −0.61, CI = [**−**0.33, 0.18], *P* = 0.55	***b*** = **−0.36,** ***t*** = **−2.7, CI** = **[−0.63, −0.10],** ***P*** = **0.01** w/o LS: *b* = **−**0.16, *t* = **−**1.5, CI = [−0.40, 0.06], *P* = 0.15	***b*** = **−0.23,** ***t*** = **−2.4, CI** = **[−0.43, −0.04],** ***P*** = **0.02** w/o LS: *b* = −0.11, *t* = **−**1.4, CI = [−0.26, 0.05], *P* = 0.17
	Age	*b* = −0.09, *t* = −0.75, CI = [−0.34, 0.16], *P* = 0.46	NA	*b* = 0.10, *t* = 0.66, CI = [−0.20, 0.40], *P* = 0.51
	ROIs	**bs** = **[−1.3, 0.09], ts** = **[−6.3, 0.45], CI** = **[(−1.7, −0.3), (−0.90, 0.49)], ps** = **[1.4** **×** **10**^**−9**^**, 0.65]**	**bs** = **[−1, 0.72], ts** = **[−6, 4.1], CI** = **[(−1.4, 0.38),(−.71, 1.1)], ps** = **[5.4** **×** **10**^**−9**^**, 0.04]**	**bs** = **[−1.1, 0.44], ts** = **[−8.3, 3.3], CI** = **[(−1.4, 0.19), (−0.86, 0.71)], ps** = **[8.4** **×** **10**^**−16**^**, 0.002]**
	Motion	*b* = −0.04, *t* = **−**0.37, CI = [−0.29, 0.20], *P* = 0.72	*b* = 0.17, *t* = 1.2, CI = [−0.10, 0.43], *P* = 0.22	*b* = 0.07, *t* = 0.70, CI = [−0.12, 0.26], *P* = 0.48

Statistical results for mixed effects regressions testing for fixed effects of ASL onset, age, region, and motion on response selectivity; subject identifier was included as a random effect given the inclusion of data from multiple regions in each regression. Left column indicates brain regions tested. The beta values, t-values, 95% confidence intervals (CI), and p-values of each tested predictor are given per regression, tested in children (*n* = 24), adults (*n* = 36), and in the full sample (*n* = 60); note that the full sample results are not independent from the child-only and adult-only results. ASL onset is a continuous variable for the age of first exposure to ASL (0 for native signers). Age is a continuous variable in regressions within the child sample, and a factor (child vs. adult) within the full sample. ROI levels include RTPJ and DMPFC (ToM selectivity analysis) or eleven individual language ROIs (language selectivity analysis; results are summarized by indicating a range of values across the eleven ROIs). Motion refers to mean translation. Significant predictors are indicated in bold; no adjustments for multiple comparisons were necessary. Source data are provided (https://osf.io/kyu3f/).

While there were no significant age of ASL onset-by-ROI interactions (Table 2), suggesting that the size of the effect did not vary significantly across RTPJ and DMPFC ROIs, post hoc analyses within each ROI found a significant effect of age of ASL onset on selectivity in children’s RTPJ only (effect in RTPJ: *b* = −0.47, *t* = −2.3, 95% CI = [−0.90, −0.05], *P* = 0.03; effect in DMPFC: *b* = −0.09, *t* = −0.39, 95% CI = [−0.58, 0.40], *P* = 0.7). A two-sided non-parametric Mann–Whitney *U* test confirmed that delayed signing children had less selective RTPJ responses than native-signing children (M(SE) selectivity in delayed signers: 25.5(8.9), native signers: 55.4(7.1), W = 97, 95% CI = [4.3, 53.6], *P* = 0.02). Reduced selectivity in children with longer language delays manifested as a higher response to the Social condition (Fig. 3). Response selectivity did not correlate with ToM behavior (Supplementary Note 11). Exploratory analyses of other ToM regions (left TPJ, precuneus, mid- and ventromedial prefrontal cortex) did not find evidence for delayed development as a function of age of ASL onset (all bs < |0.27 | , ts < |1.2 | , 95% CIs = [(−0.82, −0.46), (0.22, 0.39)], ps > 0.25; Supplementary Fig. 2, Supplementary Table 3). Age of ASL onset did not predict selectivity in any ROI in adults (bs < |0.30 | , ts < |1.1 | , 95% CIs = [(−0.85, −0.36), (0.25, 0.74)], ps > 0.28; Fig. 3, Supplementary Fig. 2, Supplementary Table 3).

While our primary measure of interest was response selectivity in ToM brain regions, we additionally measured response selectivity in language brain regions, as well as the lateralization of the response to the ASL story task in ToM and language brain regions, and inter-region correlations within and between ToM and language brain regions. The story task included Non-Sign stimuli, which served as a control condition:  non-signs were visually similar to the ASL stories but lacked higher-level linguistic features (semantic meaning and syntax). Whole-brain random effects analyses confirmed that canonical language regions were recruited to process the ASL stories (Physical Stories > Non-Signs contrast), and did not find any voxels that were differentially activated as a function of age of ASL onset (Supplementary Fig. 3). In children, age of ASL onset was not correlated with the selectivity of the response in language ROIs (*b* = −0.08, *t* = −0.61, 95% CI = [−0.33, 0.18], *P* = 0.55, Table 2; see Supplementary Fig. 3 and Supplementary Note 7 for details and similar results in group ROIs). In adults, there was an effect of age of ASL onset on the selectivity of the response in individual language ROIs (*b* = −0.36, *t* = −2.7, 95% CI = [−0.63, −0.10], *P* = 0.01). This effect was not significant upon exclusion of the four late-signing adults (Table 2), and was not observed in group ROIs (Supplementary Note 7). Age of ASL onset was not correlated with the lateralization of the language or ToM response among children or adults (Supplementary Fig. 4, Supplementary Table 4, Supplementary Note 8). Finally, there was no effect of age of ASL onset on inter-region correlations within ToM brain regions, within language brain regions, or across the two networks among children or adults (Supplementary Fig. 4, Supplementary Table 4, Supplementary Note 9).

### Nonlinguistic movie-viewing FMRI results

We measured neural responses during a nonlinguistic movie-viewing experiment. We did not observe any differences in the neural response of ToM regions or in the RTPJ specifically to the movie as a function of age of ASL onset (among children, adults, or in the full sample); see Supplementary Note 10.

## Discussion

The central aim of this study was to investigate how early linguistic experiences influence the development of theory of mind (ToM). Consistent with prior studies, we found that children with delayed access to their first language, ASL, showed subsequent impairments on behavioral tests of ToM^30–36^. However, the behavioral data leave open a key question: how does early linguistic experience influence ToM? Does linguistic experience promote children’s language abilities, which then facilitate ToM task performance, or does linguistic experience promote conceptual sophistication within ToM reasoning itself? Our fMRI data provide complementary insight and suggest that linguistic experience shapes ToM-specific development.

Consistent with the hypothesis that language abilities can limit or facilitate ToM task performance, delayed access to ASL led to deficits specifically on a linguistic ToM task. When ToM concepts were tested in a minimally linguistic format, native and delayed signers’ performance was matched. The interaction of ASL delay and task format was also observed in a subset of conceptually analogous items testing moral reasoning, and is consistent with prior observations that delayed language affects performance on linguistic more than on minimally linguistic ToM tasks^30,31,34,37^.

A straightforward interpretation might be that the complex language of ToM tasks can mask true conceptual abilities, especially in children with delayed access to language. However, we do not favor this interpretation. In this study, all children, including those with delayed access to language, actually showed an overall benefit of the linguistic format on ToM performance. That is, all children were better able to reason about false beliefs when the scenario was presented with a linguistic narrative, than when presented with pictures alone (see also ref. ^36^). Thus linguistic narratives may actually facilitate children’s ability to encode, mentally manipulate, and retrieve complex mental-state concepts^62^. Relatedly, children’s receptive ASL proficiency highly correlated with performance on the minimally linguistic ToM task, consistent with prior studies^32,34,63,64^ (though see refs. ^65,66^). Children may spontaneously create linguistic narratives to help encode nonlinguistic sequences of pictures. Overall, we do not view performance on the linguistic ToM task as an underestimate of children’s ToM competence, at least for children who are highly proficient signers. Instead, we hypothesize that the effect of delayed exposure to language was stronger on the linguistic task at least in part because it included more tests of advanced ToM. The most sophisticated ToM concepts (lies, sarcasm, and second-order false beliefs) were not tested in the minimally linguistic task. In our relatively old and linguistically proficient sample, residual ToM delays may only affect these late-acquired concepts.

In sum, our behavioral results replicate prior findings that delayed access to language can delay children’s performance on ToM tasks^29–34,36,67,68^, even in children who are fluent in ASL. However, the behavioral results cannot resolve whether linguistic experience mainly facilitates task performance or promotes development in ToM reasoning per se.

Our neuroimaging evidence offers independent and complementary insight into this question. We hypothesized that if linguistic experience directly impacts ToM development, then the consequences of delayed access to language on ToM should be observable in neural measures that capture developmental change in brain regions selective for ToM processes. Specifically, we developed an ASL story task to test whether delayed access to language correlated with delayed development of selective ToM responses in RTPJ.

When ASL signers watched a narrator tell a story about a character’s mental states, compared with stories about physical events, BOLD activity was increased in bilateral TPJ, medial prefrontal cortex, and precuneus (the “ToM network”). This pattern replicates prior studies using similar stimulus categories in English^41^, German^69^, Dutch^70^, French^71^, Chinese^72^, and Japanese^73^, presented in writing^41,69,72,73^ or aurally^70,71,74^. The ASL task is a compelling replication of selective ToM responses because the stimuli (movies of an engaging and emotive signer) are highly social in all conditions; nevertheless, activity in the ToM network was greatest when the content of the story concerned the characters’ mental states.

In hearing adults, the response in the RTPJ is particularly selective for mental-state content^41,51,53,75,76^. This brain region becomes more selective for processing mental states during childhood and, similar to the development of specialized brain regions for face perception^54^ and reading^57,58^, development of selective responses in RTPJ occurs via suppression of responses to non-preferred stimuli^48,49^. Here, in native-signing children and all adults, the RTPJ showed selective responses to stories that described mental states (Mental condition: “The pirate thought that a pile of gold was buried behind Jimmy’s house”), with low responses to Social stories that described people’s physical appearance or enduring relationships, but not their mental states (Social condition: “Sarah and Lori play together on the soccer team”).

The main aim of this study was to test whether delayed access to language affects the development of this selective response profile. If linguistic experience directly influences development of domain-specific ToM concepts, we predicted that children with delayed access to language might have less selective neural responses for ToM, even after gaining proficiency in ASL.

Consistent with this prediction, the RTPJ of delayed signing children showed high responses to both Mental and Social stories. Moreover, the length of the delay prior to access to ASL correlated with reduced selectivity of RTPJ (despite current ASL proficiency and controlling for age). There was no effect of language delay on other ToM brain regions (LTPJ, precuneus, MPFC), which are recruited during ToM reasoning but are not as selective for mental states. Overall, our results suggest that linguistic experience has a direct impact on selective responses in RTPJ. Rather than resembling the response in age-matched children, who have a similar amount of biological maturation, the response profile in RTPJ in delayed signing children resembles that previously observed in young children^48,49^, who have a similar amount of linguistic experience.

In our view, reduced selectivity of RTPJ reflects delayed development of ToM. An alternative hypothesis that we do not favor is that delayed access to language impeded children’s comprehension of the mental-state stories. The pattern of results is not consistent with this alternative. First, all children showed high RTPJ responses to the Mental stories, suggesting that the semantic content of these stories was successfully extracted. Reduced selectivity following delayed access to sign language reflects a high response to Social stories, not a low response to Mental stories (Supplementary Note 5). Second, we neither observe an effect of delayed access to ASL on development in language-related regions nor additional recruitment of brain regions that would indicate greater difficulty processing the linguistic stimuli in delayed signers (e.g., language brain regions or the multiple demand network). However, these are null results and so should be interpreted with caution. Finally, children in both groups performed well on first-order false-belief tasks presented in ASL. All in all, we suggest that all children could comprehend the Mental stories, and differences in response to the Social stories reflects a delayed developmental process of increasing response selectivity by suppressing neural responses to dis-preferred stimulus categories.

In contrast to our results, though, prior studies find that delayed access to language has broad cognitive effects^77,78^, including delayed development of language comprehension and production^79–82^, literacy^83,84^, and executive functions^85^, differences in language-related brain development^86–88^, and consequences for mental health^89,90^. This discrepancy is likely related to characteristics of our sample. All of our child participants, and a vast majority of our adult participants, were exposed to ASL after a relatively short delay (0.25–7 years), and all participants were proficient signers. Broader cognitive and emotional effects of language delay may be strongest following longer delays that more substantially impede ASL acquisition.

While our results suggest that even relatively short delays can delay the development of ToM-selective responses in RTPJ, from our results alone, it is unclear how much linguistic experience is sufficient to overcome this delay or if there is a sensitive period for the impact of linguistic experience on ToM. Prior behavioral studies have found evidence for enduring ToM delays in adults who received access to language after longer delays (i.e., after age 10 years)^91–93^, but there is also evidence that ToM delays can resolve with subsequent linguistic experience^33,68,91,94^. Here, we did not observe an enduring effect of delayed access to language on RTPJ selectivity in adults. Additional work is needed to determine whether there are prolonged effects of delayed access to language on more sophisticated aspects of ToM reasoning^92^, and/or more fine-grained aspects of the neural response (e.g., the organization of spatial response patterns within RTPJ).

A classic theoretical debate posits that ToM is either instantiated in a distinct domain-specific biological mechanism, or is constructed through conversational interactions and social relationships^95,96^. By contrast, these results suggest that ToM may be all of the above: RTPJ appears to be a domain-specific biological mechanism in which selectivity is constructed in part through language exposure. One recent version of this debate concerns the nature of early success on nonlinguistic (implicit) false-belief tasks in toddlers and infants^14^ (though see ref. ^15^). A recent functional near-infrared spectroscopy (fNIRS) study found RTPJ activation to false-belief scenarios in 7-month-old infants^97^. How can RTPJ support performance on such tasks, given our evidence that RTPJ development depends on rich linguistic experience? One possible explanation is that RTPJ is already selective for early-developing ToM concepts (e.g., reasoning about goals, perceptions, and knowledge access), and that linguistic experience shapes later-developing ToM concepts (e.g., beliefs, pragmatics) within a single continuous neural system. This account may explain the lack of differences between native and delayed signers’ RTPJ response during the movie-viewing experiment (see Supplementary Note 10 for further discussion)—but additional work is necessary to test this suggestion.

Future research is also necessary to determine whether other factors plausibly related to linguistic experience and ToM development (e.g., sibling^98^ and peer^99^ relationships, executive functions^85^) mediate the correlation between these two variables, to test which aspects of linguistic experience (e.g., mental-state vocabulary^23^, syntactic complement structures^9,23,24,100^, conversational turns^101^) promote RTPJ selectivity, and to characterize whether and which other aspects of experience influence RTPJ development^6,7^. FNIRS may be particularly suited for future studies with deaf children, given the importance of replicating these results in a larger sample and the ability to collect fNIRS data in individuals who have metallic cochlear implants.

In sum, our results point to an important role for early linguistic experience in the development of ToM reasoning in childhood. In addition to contributing to long-standing theoretical debates about ToM development, these results illustrate the importance of early exposure to and proficiency in language, as language experience facilitates social development in childhood.

## Methods

### Participants

Child participants were 21 native signers (4–12.7 years old, M(SD) = 8.19(2.2) years, 10 female), who received exposure to ASL from birth from deaf parents (15 deaf children and 6 hearing children), and 12 delayed signers (6.2–12.1 years old, M(SD) = 9.29(1.9) years, 5 female), who were born deaf to hearing parents and received exposure to ASL after an initial delay of 0.25–7 years (M(SD) = 2.9 (2.2) years).

Among adults, the native-signing participants (*n* = 20, 20–53 years old, M(SD) = 30.1(9.4)) included deaf people who had deaf parents (*n* = 10), hearing people who had deaf parents (*n* = 7), and deaf people who had hearing parents and deaf older siblings (*n* = 3). All delayed signing adults were born deaf to hearing parents (*n* = 16, 21–64 years old, M(SD) = 37.9(12.8) years, 4 female, delay before ASL exposure M(SD) = 6.5 (6.2) years). Delayed signing adults included 12 “early signers” (1.5–7 year delay, M(SD) = 3.3 (1.9) years) and 4 “late signers” (exposure to ASL at ages 11, 15, 18, and 20 years). All adult participants contributed fMRI story task data (*n* = 16 DS, *n* = 20 NS); *n* = 11 delayed signing and *n* = 18 contributed fMRI movie task data. See Supplementary Table 1 for additional information about participants.

Participants were recruited via the researchers’ social networks, by snowball sampling, and, in the case of child participants, with help from several schools for the deaf over the course of four years. All participants were screened by a native ASL signer; only fluent signers were recruited to participate.

Child participants signed an assent form; adult participants and parents of child participants signed a consent form. All assent and consent forms and experimental protocols were approved by the Committee on the Use of Humans as Experimental Subjects at MIT. All protocols comply with all relevant ethical regulations.

### Behavioral battery

All of the experimenters were highly proficient, native or near native, ASL users. While there was often a non-signing experimenter present during testing days, this experimenter generally avoided interacting with the child participants to create an ASL-only testing environment.

We measured ToM reasoning using linguistic and minimally linguistic behavioral tasks, which are both publicly available (https://osf.io/kyu3f/). The custom-made linguistic ToM task was an ASL-adapted version of a battery previously used to measure ToM in hearing children (https://osf.io/g5zpv/)^45^. The task involved watching an experimenter tell a story in ASL, and answering 25 prediction and 23 explanation questions about the mental states of the characters in the context of helping to find their snacks. Fourteen additional control questions were used to ensure task comprehension; these items were not included in the summary score. The summary score was calculated as the proportion of questions answered correctly (ToM_L_); for follow-up analyses, we calculated the proportion of control items answered correctly.

The custom-made minimally linguistic ToM task involved watching an experimenter place a series of three to five pictures on a whiteboard (using magnets) or on the floor, which presented characters undergoing a sequence of events. In the first part of the task, the experimenter would then place two pictures side by side and ask, in ASL, “What comes next?” Children responded by pointing to the picture that best completed the series (19 items). The second part of the task focused on moral reasoning (11 items). Prior to the moral reasoning items, the experimenter said, “You decide—is this good (pointing to thumb up), bad (pointing to thumb down), or okay (point to neutral thumb)?” They then showed a series of pictures, ending with a picture of a character that inflicted harm either accidentally or intentionally. Children responded by pointing to the thumbs up (good), thumbs down (bad), or neutral thumb (okay) picture, or, more frequently, by using a thumbs up, down, or sideways gesture. Children completed six practice trials before the initial sequence-completion items and an additional three practice trials before the moral reasoning items. Practice trials ensured that children understood the task but were otherwise not analyzed. The summary score was calculated as the proportion of questions answered correctly (ToM_ML_).

Linguistic and minimally linguistic ToM tasks both included (1) easy items, which involved reasoning about desires, emotions, and true beliefs, (both tasks: *n* = 14), (2) false-belief items, (ToM_L_: *n* = 9, ToM_ML_: *n* = 5), and (3) moral judgment items (ToM_L_: *n* = 10, ToM_ML_: *n* = 11). The linguistic task additionally included (4) hard items that involved reasoning about lies and second-order false beliefs (*n* = 7), mistaken referents (*n* = 4), and non-literal speech (e.g., sarcasm; *n* = 4). These four categories were created post hoc to sensitively capture differences in conceptual content while minimizing the total number of categories (maximizing items per category); performance on each item category was calculated as the proportion of items answered correctly.

Children additionally completed the American Sign Language Receptive Skills Test (ASL-RST)^59^. After completing a vocabulary check (*n* = 20 trials), children watched an adult signing in a movie and responded by pointing to the picture (out of a 4-picture array) that corresponded to the signed utterance. Children completed three practice trials after the vocabulary check and prior to the receptive skills test. Two items were ultimately excluded from analyses (item 37: BOX DOG-IN-FRONT, item 42: INTERSECTION HOUSE-TOP-RIGHT) because more than 75% of participants (and more than 70% of the native signers) answered these items incorrectly. The summary score reflects proportion correct on the remaining items. The ASL-RST neither measures understanding of syntactic complements nor includes mental-state vocabulary in any of the test items.

Children also completed a standardized task of non-verbal IQ (KBIT-II^102^) and, when possible, a spatial working memory task (computerized CORSI^103^; *n* = 24) and an executive functions task (custom-made computerized flanker task modeled after a prior study^104^; *n* = 24).

### ASL story FMRI task

Prior to the fMRI scan, children watched a movie of their choice in a mock scanner while lying still on their back for 10–15 min. Hearing children (native-signing children of deaf adults) listened to a recording of scanner sounds during the mock scan. If participants moved during the mock scan, their movie paused for three seconds, reminding and training them to stay still.

During the fMRI scan, participants watched movies of a woman telling stories in ASL, which involved characters and their mental states (Mental condition), characters and their physical appearance or enduring social relationships (Social condition), or descriptions of physical objects and events in the world (Physical condition). Mental stories included relatively simple mental-state verbs (e.g., know, want, surprised) and did not evoke reasoning about humor, sarcasm, or pragmatics. A subset of the stimuli (24/42 stories) was directly translated from English stories previously used to measure neural responses in hearing children and adults^48,49^; the remaining stimuli were similar in style (see https://osf.io/kyu3f/ for all stimuli). All 42 stories were rated for linguistic features (syntactic complexity, the number of signs, number of verbs, and, for Mental and Social stories, number of role shifts), psychological features (how easy to understand, how interesting), and imageability by 10 Deaf native-signing adults who were naive to the experimental hypotheses. These ratings were used to ensure that stimuli were matched across conditions. Stories were told using simple language, in an enthusiastic, narrative way.

During the scan, participants answered an orthogonal question about the stimuli in order to encourage engagement (Supplementary Note 2). Child participants viewed 24 stories (8 per condition) across four 8.3-min runs. Adult participants viewed 30 stories (10 per condition) across five 10.3-min runs. All children saw the same 8 stories per condition; each adult participant saw 10 of 14 stories per condition.

Participants also saw 8 (child) or 20 (adult) clips of non-signs; the non-sign stimuli were used in control analyses of language processing. Processing pronounceable non-words engages many of the low-level processes required for language processing, such as visual/auditory processing, phonological recognition and composition, and working memory, without recruiting higher-level processes, such as lexical access, word and sentence level composition, syntactic structure building, or semantic computation. Non-sign stimuli consisted of a Deaf native signer (the same person who signed the story stimuli) signing strings of nonsense signs. Non-signs were phonologically possible, but meaningless, signs, paired with grammatically possible, but meaningless, facial expressions. The adult paradigm included two Non-sign conditions consisting of strings of three or eight non-signs (ten stimuli each); the child paradigm included a single Non-sign condition with a series of five non-signs. Native ASL signers screened the non-sign stimuli to identify and exclude signs similar to known signs or regional slang. Non-signs and stories were matched for low-level visual properties (e.g., motion energy) and duration. As with the story stimuli, participants answered an orthogonal question about the stimuli after the initial non-sign segment, and only the initial segment was used for subsequent analyses. We used the Physical story > 8-Non-Sign (adult) and Physical story > 5-Non-Sign (child) contrasts to study the neural responses of regions recruited for language processing at the word and sentence level^105–107^.

Stimuli were presented in Matlab 2010a running on an Apple MacBook Pro. Stimuli were counterbalanced across runs and participants. Each run included six 60-s blocks (two per condition), as well as 10 s of rest at the beginning and end of each run. The order of conditions in each run was palindromic (e.g., A B C D D C B A) and counterbalanced across runs.

An experimenter in the control room monitored participants during the scan. For child participants, a second experimenter stood in the MRI room near the participant’s feet. If the participant moved noticeably during the scan, this experimenter would place her hand on the child’s leg as a reminder to stay still. The experimenter in the control room communicated with participants in between runs by signing via live video. Participants used the button box to answer questions like “Are you okay?” and “Are you ready to continue?” Participants were also given a squeeze ball that would alert the experimenters in the control room if they wanted to stop the scan. Six children did not complete more than one run of the story task and were excluded from analyses (Supplementary Table 1).

### Nonlinguistic movie-viewing FMRI task

After the story task, participants watched a silent version of Partly Cloudy, a 5.6-min nonlinguistic animated movie^108^. A short description of the plot can be found online (https://www.pixar.com/partly-cloudy#partly-cloudy-1). The stimulus was preceded by 10 s of rest. Participants were instructed to watch the movie and remain still. Previous work has used this movie to localize^44^ and study developmental change in ToM brain regions^45^. Seven adult and two child participants did not complete the movie-viewing scan (Supplementary Table 1).

### FMRI data acquisition

Whole-brain structural and functional MRI data were acquired on a 3-Tesla Siemens Tim Trio scanner located at the Athinoula A. Martinos Imaging Center at MIT, using custom 32-channel phased-array head coils made for children^109^ or the standard Siemens 32-channel head coil. T1-weighted structural images were collected in 176 interleaved sagittal slices with 1-mm isotropic voxels (GRAPPA parallel imaging, acceleration factor of 3; adult coil: FOV: 256 mm; pediatric coils: FOV: 192 mm). Functional data were collected with a gradient-echo EPI sequence sensitive to Blood Oxygen Level Dependent (BOLD) contrast in 3 mm isotropic voxels with a 20% slice gap (*n* = 7 adults, *n* = 28 children) or 3.13-mm isotropic voxels with no slice gap (*n* = 29 adults, *n* = 1 child) in 32 interleaved near-axial slices aligned with the anterior/posterior commissure, and covering the whole brain (EPI factor: 64; TR: 2 s, TE: 30 ms, flip angle: 90**°**); all functional data were subsequently upsampled in normalized space to 2-mm isotropic voxels. Prospective acquisition correction was used to adjust the positions of the gradients based on the participant’s head motion one TR back^110^. 310 (adults) or 250 (children) volumes were acquired in each run of the story task. 155 volumes were acquired during the single run of the movie-viewing task. Four dummy scans were collected to allow for steady-state magnetization in each run.

### FMRI data analysis

All analysis decisions (including preprocessing, region of interest selection and definition, motion exclusion and treatment, calculation of selectivity indices) and planned analyses for the story task were pre-registered via the Open Science Framework (OSF) after data collection was completed (ToM: https://osf.io/mhgp8; Language: https://osf.io/7y263)^111^. Story and movie task analyses were constrained by methods used in prior studies in order to facilitate comparisons across studies^45^ (https://osf.io/wzd8a/). Post hoc and exploratory analyses are specifically noted as such.

FMRI data were analyzed using SPM8 (version R4010; http://www.fil.ion.ucl.ac.uk/spm) and custom software written in Matlab (MathWorks, Natick, MA). Functional images were registered to the first image of each run; that image was registered to each child’s anatomical scan, and each child’s anatomical scan was normalized to a common brain space (Montreal Neurological Institute (MNI) template). Previous research suggests that anatomical differences between children as young as 7 years are small relative to the resolution of fMRI data, which supports usage of a common space between adults and children of this age (for similar procedures with children under age seven years, see refs. ^45,112^; for methodological considerations, see ref. ^113^). All data were smoothed using a Gaussian filter (5-mm kernel).

Motion artifact timepoints were identified using the ART toolbox (https://www.nitrc.org/projects/artifact_detect/)^114^ as timepoints when there was (1) more than 2 mm of motion or (2) a fluctuation in global signal >3 SD from the mean. Runs were excluded if one-third or more of the timepoints were identified as motion artifacts. Participants were excluded from analyses of the story task if they had fewer than two runs of usable data (*n* = 3 children). The movie task consisted of one run; three children were excluded for excessive motion during this task. See Supplementary Note 3 for additional details about motion and Supplementary Table 1 and Supplementary Fig. 5 for amount of motion per participant. Data were high-pass filtered with a cutoff of 500 (story task) or 100 s (inter-region correlation analyses for both tasks, see below), in order to remove low-frequency noise, after interpolating over artifact timepoints^115,116^. We additionally implemented SPM’s image scaling.

### Analyses of the ASL story FMRI task

We used a general-linear model to analyze BOLD activity of each participant as a function of condition. Data were modeled in SPM8 using a standard hemodynamic response function (HRF). Boxcar regressors for each condition and the response period were convolved with the standard HRF, and nuisance covariates were included for run effects, motion artifact timepoints, and signal of no interest (five PCA-based regressors generated with aCompCor^117^ from individually tailored white matter masks eroded by two voxels in each direction).

We conducted whole-brain random effects analyses on the Mental > Physical and Physical > Non-Signs contrasts in order to visualize regions active for ToM and language processing, respectively, and to test for differences in activation by age of ASL onset. Analyses were corrected for multiple comparisons by estimating the false-positive rate via 5000 Monte Carlo permutations using the SnPM5b toolbox for SPM5 (version 1111; http://www.fil.ion.ucl.ac.uk/spm/software/spm5/), at *P* < 0.05. For whole-brain analyses of delayed signing children only (Story Task: *n* = 8; Movie Task: *n* = 9), the maximum number of Monte Carlo permutations (given the small sample size) were used (256 and 512 permutations, respectively).

We conducted Region of Interest (ROI) analyses on responses from the right temporoparietal junction (RTPJ) and dorsal medial prefrontal cortex (DMPFC). We focused on these ROIs because previous work suggests that development of these regions is related to behavioral ToM in childhood^48,60^. We additionally conducted exploratory analyses of other ToM ROIs, including left temporoparietal junction (LTPJ), middle and ventral medial prefrontal cortex (M/VMPFC), and precuneus (PC). Individual ROIs were defined as contiguous (minimum *k* = 10) suprathreshold (*P* < 0.001) voxels within a 9-mm radius sphere of the peak voxel to the Mental > Physical contrast within previously defined region search spaces. Region search spaces were defined in a random effects analysis of a False-Belief > False-Photograph contrast in a independent group of 462 neurotypical adults^118^. The use of individually defined ROIs limits the potential that different fits (and therefore normalization) to the MNI template lead to different results across ages, and helps ensure that response selectivity is not underestimated in individual participants. All individual ToM ROIs were similarly prevalent across delayed and native signers, and did not differ in size or position as a function of ASL onset (Supplementary Note 4 and Supplementary Table 2). We extracted the mean beta value per condition per region, and calculated selectivity as (Mental–Social)/(Mental–Physical)*100. Because the selectivity measure captures the relative difference between Mental and Social, it is independent from individual ROI definition (which used the Mental > Physical contrast). This calculation has been used in previous studies of ToM brain region development^48^ (https://osf.io/wzd8a/). Based on previous analyses, we expected the selectivity measure to be between −50 and 200 in individual ROIs and excluded values outside of this range (https://osf.io/mhgp8; *n* = 1 NS adult DMPFC value was excluded, selectivity = −64.2). Analyses of a small pilot dataset suggest that RTPJ response selectivity is a reliable measure within individual child participants (Supplementary Note 6 and Supplementary Fig. 6). We additionally conducted analyses in group ToM ROIs, which were defined in an independent sample; see Supplementary Note 5, Supplementary Fig. 1, Supplementary Table 2, and Supplementary Table 3 for details and results.

To test whether language delay affected responses in cortical language regions, we also defined group language ROIs as 10-mm spheres drawn around peak coordinates from a prior study^105^: including left inferior frontal gyrus, orbital inferior frontal gyrus, medial frontal gyrus, superior frontal gyrus, anterior temporal lobe, middle anterior temporal lobe, middle posterior temporal lobe, posterior temporal lobe, angular gyrus, and right middle anterior temporal lobe and middle posterior temporal lobe; cerebellar ROIs were excluded due to lack of coverage. See Supplementary Table 2 for peak coordinates. Selectivity in these regions was calculated as the mean beta value to Physical–Non-Sign conditions, multiplied by 100. We also conducted a parallel analysis in ROIs spatially tailored to each individual by extracting responses from the 50 voxels with the highest *T*-values to the Physical > Non-Sign contrast within these 11 regions (Supplementary Note 7).

We additionally measured the lateralization of ToM and language neural responses^119^, and conducted inter-region correlation (IRC) analyses on the responses within and across group ToM and language brain regions^45^. Because of paradigm differences (in the non-sign stimuli) between children and adults, IRC analyses were conducted in each age group separately. See Supplementary Notes 8 and 9 for detailed methods for lateralization and IRC analyses.

### Analyses of the movie-viewing FMRI task

Analyses of the movie-viewing task followed methods developed in a prior study^45^. See Supplementary Note 10 for methods and results.

### Statistical regressions

We used linear mixed effects regressions to test if each of these neural response properties in ToM and language brain regions differed as a function of age of first exposure to ASL, a continuous variable ranging between 0.25–7 years in delayed signing children, and 1.5–20 years in delayed signing adults. Age of ASL onset for native signers was zero. Statistical analyses were conducted in Matlab 2017a (MathWorks, Natick, MA) and R 3.3.3 (https://www.r-project.org/; package nlme for mixed effects regressions). We conducted regressions within children, within adults, and, when possible, across the full sample. We included age group (regressions across the full sample; child vs. adult) or age (regressions within children only; continuous variable) as a covariate. All planned regressions on selectivity during the story task included the data from both ToM ROIs (RTPJ, DMPFC), and tested for a significant effect (and interaction) of ROI; regressions on selectivity in language ROIs similarly included ROI as a covariate. As specified in the analysis plan, we first tested for significant age (or age-group)-by-ASL onset interactions, and if the interaction term was not significant, removed it from the regression. Regressions included motion (mean translation) as a between-subject predictor and a subject identifier as a random effect in those that included non-independent measurements (e.g., data from two ROIs per subject). Continuous regression variables were standardized such that the units of the regression beta coefficients are the same.

### Statistics and reproducibility

All statistical tests were conducted on the sample of delayed and native signers recruited for this experiment. This experiment has not yet been repeated or reproduced.

### Reporting summary

Further information on research design is available in the Nature Research Reporting Summary linked to this article.

## Supplementary information

Supplementary Information

Peer Review File

Reporting Summary

## Data Availability

Because these data were collected up to eight years ago, and prior to the normalization of data sharing, the conditions of our ethics approval did not include public archiving of individual raw MRI or behavioral data. Processed data, which enable reproducing all statistical results and figures, fMRI story stimuli, and behavioral ToM tasks are publicly available on OSF (https://osf.io/kyu3f/). To ensure anonymity of participants, participant ages and ages of ASL onset have been z-scored. A reporting summary for this Article is available as a Supplementary Information file. The corresponding author welcomes any additional requests for materials. Source data are provided with this paper.

## Source data

Source Data

## Code availability

Analysis code for reproducing all statistical results and figures is available on OSF (https://osf.io/kyu3f/). 
