# Supplementary material for: Reduced neural selectivity for mental states in deaf children with delayed exposure to sign language
Source: Nat Commun. 2020 Jun 26;11:3246. doi: 10.1038/s41467-020-17004-y (PMC7319957; doi:10.1038/s41467-020-17004-y)
Supplement: Supplementary file 1 — Supplementary Information [file 41467_2020_17004_MOESM1_ESM.pdf]

Supplementary Information

Reduced Neural Selectivity for Mental States in Deaf Children with Delayed Exposure to Sign Language

Richardson, H., Koster-Hale, J., Caselli, N., Magid, R., Benedict, R., Olson, H., Pyers, J., Saxe, R.

Supplementary Note 1
We tested for a correlation between receptive ASL and false belief item performance in the two tasks, given almost all studies on the relationship between language and ToM use the false belief task. Receptive ASL correlated with false belief performance when measured via the linguistic task (b=.55, t=2.7, 95% CI=[.14,.96], p=.01) but not when measured via the minimally linguistic task (b=.06, t=.29, 95% CI=[-.35,.46], p=.78). When controlling for age, this correlation was not significant in either task (ToM_L: effect of receptive ASL: b=.34, t=1.7, 95% CI=[-.08,.76], p=.11, effect of age: b=.44, t=2.5, 95% CI=[.07,.80], p=.02; ToM_{ML}: effect of receptive ASL: b=.06, t=.27, 95% CI=[-.37,.48], p=.79, effect of age: b=.33, t=1.7, 95% CI=[-.06,.73], p=.09, receptive ASL-by-age interaction: b=.52, t=2.9, 95% CI=[.15,.89], p=.008).

Supplementary Note 2
To encourage engagement during the story task, stories were presented in two consecutive segments: the main story (29-41s) and a final sentence containing the story ending or the ending of an unrelated story (4-8s). Only the main story, and not the ending, was included in the fMRI data analysis. Half of the presented stories/non-signs were followed by the correct ending ("Yes" response). Incorrect endings were drawn randomly from another story. After each non-sign stimulus, participants saw an identical or novel sequence of non-signs and had to judge whether the final ending sequence matched the signs in the initial sequence. After the stimuli completed there was a 3s pause during which participants responded to indicate whether the ending fit the main story by pushing one of two buttons ("Yes" or "No"). An image of a check (left) and an "X" (right) was displayed to remind participants which button corresponded to "Yes" and "No" responses. After the participant responded, there was a rest period (8-24s, such that each block lasted 60s). All participants were introduced to the task and completed four practice trials prior to the scan.

Behavioral performance on the ASL story task was measured via accuracy (proportion of questions answered correctly) on trials from included functional runs only; trials from runs that were excluded due to excessive motion were not analyzed. Participants generally performed well above chance on this task (M(SD) per condition: Children: Mental: .80(.23), Social: .79(.19), Physical: .68(.23), 5-Non-Signs: .83(.22); Adults: Mental: .87(.15), Social: .87(.17), Physical: .83(.17), 3-Non-Signs: .92(.11), 8-Non-Signs: .86(.16)). The linguistic conditions (Mental, Social, and Physical) were used to test for behavioral performance differences by age of ASL onset and age. Among children, there was no effect of age of ASL onset on accuracy (b=-.21, t=-1.4, 95% CI=[-.51,.09], p=.18), but older children were more accurate (effect of age: b=.37, t=2.5, 95% CI=[.07,.68], p=.02), and children performed worse on Physical condition trials (effect of Physical condition, compared to Mental: b=-.54, t=-2.7, 95% CI=[-.93,-.16], p=.009; effect of Social condition: b=-.03, t=-.13, 95% CI=[-.41,.35], p=.89; no significant age-by-ASL-onset or age of ASL onset-by-condition interactions). In a second regression that additionally included receptive ASL score, the results were unchanged and there was no effect of ASL proficiency (b=.15, t=.91, 95% CI=[-.50,.11], p=.37). Among adults, there was an effect of age of ASL onset such that adults who experienced a longer delay before exposure to language performed worse overall on the task (b=-.53, t=-4.5, 95% CI=[-.76,-.29], p=.0001), and an effect of condition such that adults performed marginally worse on Physical condition trials (effect of Physical condition, compared to Mental: b=-.25, t=-1.8, 95% CI=[-.52,.02], p=.08; effect of Social condition: b=.002, t=.01, 95% CI=[-.27,.27], p=.99; no significant age of ASL onset-by-

condition interaction). This pattern of results remained the same when excluding the four (LS) adults who received exposure to ASL after age ten years (effect of age of ASL onset: b=-.34, t=-2.7, 95% CI=[-.60,-.09], p=.01, effect of Physical condition (compared to Mental): b=-.32, t=-1.8, 95% CI=[-.68,.04], p=.08, effect of Social condition: b=-.04, t=-.22, 95% CI=[-.40,.32], p=.83; no significant ASL-onset*condition interactions).

Supplementary Note 3

The mean translation of the brain between fMRI images is a direct measure of the amount of motion contaminating the data, and therefore a good proxy for overall data quality. We tested for differences in mean translation based on age of ASL onset or age, and included this measure in all linear regressions including neural measures (as pre-specified in the analysis plan: https://osf.io/kyu3f/). In prior pediatric studies in our research group, mean translation has been highly correlated with another measure of data quality: the number of artifact data points identified per participant (e.g., r>.5). In the current dataset, these two measures of data quality were not correlated in either task, in children or adults (rs<.31, ts<1.5, ps>.15).

Among children, mean translation during the story task was not significantly correlated with age or ToM behavior (Age: $r_s(22)$=-.05, t=-.23, 95% CI=[-.44,.36], p=.82; ToM_L: $r_k(19)$=.05, t=.23, 95% CI=[-.39,.47], p=.82; ToM_{ML}: $r_k(22)$=-.06, t=-.28, 95% CI=[-.45,.35], p=.79), or with age of ASL onset ($r_s(22)$=-.20, t=-.97, 95% CI=[-.56,.22], p=.34). Among adults, age of ASL onset was positively correlated with mean translation during the story task ($r_s(34)$=.78, t=7.2, 95% CI=[.60,.88], $p=2.3x10^{-8}$). This correlation remained significant when excluding the four LS adults who received exposure to ASL after age ten years ($r_s(30)$=.59, t=4.0, 95% CI=[.30,.78], p=.0004).

Among children, mean translation during movie-viewing was not significantly correlated with age ($r_s(26)$=-.15, t=-.78, 95% CI=[-.50,.23], p=.44) or min-linguistic ToM score ($r_k(26)$=.08, t=.39, 95% CI=[-.31,.44], p=.70), but was significantly negatively correlated with performance on the linguistic ToM task ($r_k(21)$=-.49, t=-2.6, 95% CI=[-.75,-.10], p=.02). Mean translation during the movie task was not correlated with age of ASL onset in children ($r_s(26)$=.03, t=.14, 95% CI=[-.35,.40], p=.89), but there was a significant positive correlation between motion during the movie task and age of ASL onset among adults ($r_s(27)$=.74, t=5.6, 95% CI=[.51,.87], $p=5.5x10^{-6}$), even when excluding LS adults who received access to ASL after age ten years ($r_s(24)$=.63, t=4.0, 95% CI=[.33,.82], p=.0005).

Supplementary Note 4

In order to confirm that differences in response selectivity in individual (RTPJ) ROIs were not driven by failure to capture individual regions of interest in delayed signers, or by differences in fit to group ROI spaces across groups or by age, we tested whether prevalence, position of peak voxel, or size of individually defined ROIs varied by age of ASL onset and age.

Individual RTPJ ROIs were identified in 8/8 delayed signing and 15/16 native signing children (adults: 16/16 DS, 20/20 NS; see Supplementary Table 2 for all ToM ROIs). Prevalence of

individual ROI identification did not differ across native and delayed signers, in children or in adults (Fisher's exact tests; all ps>.11).

Replicating prior work (e.g., Gweon et al., 2012), the position of individually defined ROIs did not differ between child and adult participants (**RTPJ:** ts<.72, ps>.4; **all other ToM ROIs:** ts<|1.98|, ps>.05). The position of individually defined ROI peaks was also similar across native and delayed signers: peak X, Y, and Z coordinates did not differ by age or age of ASL onset in any ToM ROI among children (**RTPJ:** effects of age: ts<|1.4|, ps>.2, effects of age of ASL onset: ts<|.32|, ps>.2; **all other ToM ROIs:** all effects ts<|1.8|, ps>.10). Among adults, RTPJ X, Y, and Z coordinates did not differ by age of ASL onset (ts<.62, ps>.54), but the X coordinate of MMPFC was larger (more right-lateralized) in adults who experienced a longer language delay (t=2.3, p=.03; all other ToM ROIs/coordinates: ts<|1.9|, ps>.06). This effect would not survive correcting for multiple comparisons (Bonferroni correction for 18 tests (3 coordinate values for 6 ToM ROIs): α =.003). In ROI analyses, there were no differences in response selectivity in MMPFC among adults by age of ASL onset (individual ROI: b=.05, t=.17, 95% CI=[-.53,.62], p=.87; group ROI: b=-.10, t=-.35, 95% CI=[-.66,.47], p=.73). The overall similarity of the location of activation to Mental > Physical by age and age of ASL onset is illustrated in Supplementary Figure 1 and detailed in Supplementary Table 2.

The size of individually defined RTPJ ROIs did not differ between children and adults (**RTPJ:** t=-1, p=.30; **all other ToM ROIs:** ts<|1.8|, ps>.08), with the exception of LTPJ, which was significantly smaller in children (t=-3.4, p=.001). Among children, the size of individually defined ROIs did not vary by age (**RTPJ:** t=1.2, p=.25; **all other ToM ROIs:** ts<1.8, ps>.09). Age of ASL onset did not predict RTPJ size among children (t=-.44, p=.66), but did predict size of precuneus ROIs (t=-2.1, p=.0497; all other ToM ROIs: ts<1.1, ps>.3). This result would not survive correcting for multiple comparisons (6 ROIs, α =.008), and there were no differences by age of ASL onset in selectivity of precuneus among children (individual ROI: b=-.03, t=-.17, p=.86; group ROI: b=-.29, t=-1.3, p=.20). ROI size was not predicted by age of ASL onset among adults (**RTPJ:** t=-.20, p=.84; **all other ToM ROIs:** ts<|1.5|, ps>.14).

Supplementary Note 5
We tested if delayed access to linguistic input results in delayed or disrupted functional specialization of ToM brain regions using pre-specified group regions of interest. Group ROIs enable measuring a response in every individual participant, but each individual's measure is less tailored to their functional response profile and anatomy. Group ROIs were 10mm spheres drawn around the peak coordinates from the independent dataset used to create search spaces (Dufour et al., 2013), excluding voxels that overlapped with language group ROIs (see below and Supplementary Table 2 for details about language group ROIs). We used these group ROIs for easy comparison of results to other studies (https://osf.io/wzd8a/). Unlike individual ROIs, the voxels analyzed in group ROIs did not necessarily respond more to the Mental condition compared to the Physical condition (voxels in group ROIs were not selected based on their functional response profile). Thus, we extracted average beta values and calculated selectivity as (Mental – Social) * 100. As with individual ROIs, we pre-registered an expected range of selectivity values for group ROIs (-50 – 100). However, this pre-registered range appeared to be too conservative in the current sample: using this range would result in the exclusion of several

data points, especially from ROIs in the prefrontal cortex (1 RTPJ, 3 LTPJ, 7 DMPFC, 12 MMPFC, 4 VMPFC). Given the small sample size of the current dataset, the small sample size of the dataset used to pre-register the range of selectivity values, and the possibility that selectivity values may vary meaningfully in the current sample, we examined the histogram of selectivity values in the current sample (all participants, all ToM group ROIs), and included all selectivity values between -100 and 126 (the maximum) in group ROI analyses. This resulted in excluding 3 DMPFC, 3 MMPFC, and 2 VMPFC selectivity values. See Supplementary Table 2 for additional information about group regions of interest.

As with individual ROI analyses, we initially conducted planned analyses in RTPJ and DMPFC group ROIs. Among child participants, functional selectivity decreased as a function of age of ASL onset (age of ASL onset: b=-.40, t=-2.2, 95% CI=[-.75,-.04], p=.04; age: b=.22, t=1.3, 95% CI=[-.13,.57], p=.22; ROI: b=.29, t=1.6, 95% CI=[-.07,.65], p=.13; motion: b=.03, t=.20, 95% CI=[-.30,.37], p=.85). The effect of age of ASL onset remained significant when additionally including non-verbal IQ as a covariate (age of ASL onset: b=-.49, t=-2.3, 95% CI=[-.91,-.08], p=.03; non-verbal IQ: b=-.18, t=-.89, 95% CI=[-.57,.21], p=.38). As with individual ROIs, the effect of age of ASL onset was only significant in the RTPJ (Supplementary Table 3), and appeared to manifest as delayed suppression of the RTPJ response to the Social stories. In a post-hoc exploratory analysis, age of ASL onset correlated with the magnitude of response (beta estimate) for the Social condition (r_S(22)=.57, 95% CI=[.22,.90], p=.004), but not for the Mental condition (r_S(22)=.09, 95% CI=[.36,.55], p=.66). That is, children who experienced longer delays had higher RTPJ responses to the Social stories.

The negative effect of age of ASL onset was not significant in the full sample (children and adults; age of ASL onset: b=-.22, t=-1.4, 95% CI=[-.52,.09], p=.16; age group: b=-.03, t=-.14, 95% CI=[-.43,.50], p=.89; ROI: b=.03, t=.23, 95% CI=[-.25,.31], p=.82; motion: b=.16, t=1.0, 95% CI=[-.14,.45], p=.30), and there was no effect of delayed access to language on response selectivity among adults (age of ASL: b=-.10, t=-.42, 95% CI=[-.52,.33], p=.68; ROI: b=-.17, t=-.83, 95% CI=[-.59,.24], p=.41; motion: b=.05, t=.22, 95% CI=[-.38,.47], p=.82).

We did not find evidence for a relationship between response selectivity and performance on either ToM behavioral task (ToM_L: b=.14, t=.66, 95% CI=[-.27,.55], p=.52, ROI: b=.33, t=1.8, 95% CI=[-.03,.68], p=.08, motion: b=.02, t=.11, 95% CI=[-.39,.43], p=.91; ToM_{ML}: b=-.04, t=-.19, 95% CI=[-.42,.35], p=.85, ROI: b=.29, t=1.6, 95% CI=[-.07,.65], p=.12, motion: b=.10, t=.52, 95% CI=[-.27,.47], p=.61).

Supplementary Note 6

We collected a small pilot fMRI dataset consisting of eight neurotypical 9-12 year old hearing children (M(SD) age: 10.5(1.3) years; 4 females; 1 LH, 1 Ambidextrous) in order to determine if response selectivity could be reliably measured within individual participants. Two additional children were scanned and excluded from analyses due to failure to complete the scan (n=1) and excessive motion (n=1). Participants were recruited from the local community (Boston, MA, USA), were native speakers of English, had no known neurological or cognitive disabilities, and had normal or corrected-to-normal vision. Participants signed an assent form and parents of participants signed a consent form approved by the Committee on the Use of Humans as

Experimental Subjects (COUHES) at MIT. Consent, assents, and experimental protocols were approved by COUHES.

Participants listened to two matched versions of the fMRI story task (T1, T2) in interleaved runs, during a single visit. The two tasks contained distinct sets of stories matched for linguistic and social content. FMRI data acquisition and data analysis procedures were identical to the procedures used in the current study. All pilot participants were scanned with the larger pediatric head coil. Participant motion did not differ across the two task versions (M(SD) number of artifact timepoints: T1: 56.3 (30.5); T2: 61.4 (37.1); effect of task version: b=.16, t=.74, 95% CI=[-.31,.62], p=.48). Both tasks evoked equally selective responses to Mental stories in group RTPJ and DMPFC ROIs (effect of task version: b=.12, t=.44, 95% CI=[-.42,.66], p=.67, effect of ROI: b=.08, t=.27, 95% CI=[-.46,.61], p=.79, effect of motion: b=.48, t=2.2, 95% CI=[-.03,.99], p=.07). We tested whether response selectivity was reliable across the two task versions in group ROIs in order to maximize number of data points. Response selectivity was reliable in RTPJ, such that selectivity was correlated within individuals across the two tasks (**group RTPJ ROI:** *r*(6)=.75, 95% CI=[.08,.95], p=.03; **group DMPFC ROI:** *r*(6)=.20, 95% CI=[-.58,.79], p=.63). In a mixed effects linear regression testing for an effect of ROI (and including subject ID as a random effect), selectivity was not significantly more reliable in RTPJ than in DMPFC (interaction term: b=1.02, t=1.9, p=.12). See Supplementary Figure 6.

Supplementary Note 7

The Physical > Non-Sign contrast value was generally positive in individual language regions of interest (M(SE) contrast value: adults: 3.81(.18), children: 3.83(.26); t-test (mu=0): adults: t(35) = 21.46, 95% CI=[3.4,4.2], p<2.2x10^{-16}; children: t(23)=14.84, 95% CI=[3.3,4.4], p=2.9x10^{-13}). Responses in children were not significantly different from adults (age group: b=-.02, 95% CI=[-.31,.28], t=-.12, p=.91, motion: b=-.09, t=-1.2, p=.24). Among children, there was no effect of age on the mean contrast value (age: b=-.10, t=-.87, 95% CI=[-.35,.14], p=.39, motion: b=-.03, t=-.25, 95% CI=[-.27,.21], p=.80). Effects of age of ASL onset are reported in the main text and in Table 2.

The language response to the Physical > Non-Signs contrast was also generally positive in group regions of interest (M(SE) selectivity (beta difference*100): adults: 16.84(2.2), children: 17.93(4.7); t-test (mu=0): adults: t(35)=7.62, 95% CI=[12.4,21.3], p=6.12x10^{-9}; children: t(23)=4.10, 95% CI=[8.9,27.0], p=.0004). There was no effect of age of ASL onset on the language response in group ROIs in children (ASL-onset: b=-.09, t=-.87, 95% CI=[-.31,.12], p=.39; age: b=.08, t=.75, 95% CI=[-.13,.29], p=.46; ROIs: -1.19<bs<-.48, -4.8<ts<-1.9, 95% CIs = [-1.65--.95, -.71--.005], 2.9x10^{-6}<ps<.053; motion: b=.04, t=.42, 95% CI=[-.17,.26], p=.68), in adults (ASL-onset: b=-.03, t=-.22, 95% CI=[-.28,.22], p=.83; ROIs: -1.3<bs<-.10, -6.6<ts<-.52, 95% CI=[-1.7--.49, -.93--.09], 1.6x10^{-10}<ps<.60; motion: b=.06, t=.47, 95% CI=[-.19,.31], p=.64), or in the full sample (ASL-onset: b=-.06, t=-.71, 95% CI=[-.24,.11], p=.48; age group: b=.002, t=.01, 95% CI=[-.27,.27], p=.99, ROIs: -1.2<bs<-.35, -7.5<ts<-2.2, 95% CI=[-1.5--.64, -.87--.04], 2.2x10^{-13}<ps<2.6x10^{-2}; motion: b=.08, t=.93, 95% CI=[-.09,.25], p=.35). See Supplementary Figure 3.

Supplementary Note 8

We measured the lateralization of the neural response to the Mental > Physical (ToM) contrast in ToM regions, and to the Physical > Non-Sign (Language) contrast in language regions. For the ToM lateralization analysis, we created a large ROI encompassing the bilateral temporal lobe from publicly available right hemisphere search spaces (http://saxelab.mit.edu/ToMgroupMaps.php; Dufour et al., 2013); the right hemisphere was flipped to create the left hemisphere ROI. For the language lateralization analysis, we created a composite language ROI in the left hemisphere using the nine left hemisphere ROIs described in the main text (Fedorenko et al., 2010), and the mirror image of these regions in the right hemisphere. The lateralization index (LI) was calculated as the number of suprathreshold voxels in the left hemisphere minus the number of suprathreshold voxels in the right hemisphere, divided by the sum of the number of suprathreshold voxels in the left and right hemispheres ($(NumVox_L - NumVox_R)/(NumVox_L + NumVox_R)$) (Desmond et al., 1995). We used a threshold of $p<.001$, uncorrected, and confirmed that results were not threshold dependent by repeating analyses at $p<.01$. We planned to exclude participants if the denominator was smaller than 20, indicating fewer than 20 suprathreshold voxels, bilaterally; zero participants were excluded based on this criterion. Large positive LI values indicate strong left lateralization, whereas an LI of zero indicates no response lateralization.

In children and adults, the response to Mental > Physical was not lateralized to either hemisphere (M(SE) laterality index: adults: .10(.05), children: -.08(.09); t-tests (mu=0): all ts<|1.9|, ps>.06). Responses in children were marginally more right-lateralized than adults (age group: b=.48, t=1.9, 95% CI=[-.04,1.0], p=.07; motion: b=.05, t=.42, 95% CI=[-.20,.31], p=.68). Among children, there was no effect of age on response lateralization (age: b=.02, t=.09, 95% CI=[-.43,.47], p=.93; motion: b=-.02, t=-.78, 95% CI=[-.62,.28], p=.44). Analyses conducted at a more lenient threshold (p=.01) yielded the same pattern of results.

The language response to Physical > Non-Signs was significantly left-lateralized among children and adults (M(SE) laterality index: adults: .12(.05), children: .20(.07); t-test (mu=0): adults: $t(35)=2.4$, 95% CI=[.02,.23], p=.02, children: $t(23)=2.8$, 95% CI=[.05,.35], p=.01). The laterality index did not differ significantly by age group (effect of age group: b=-.22, t=-.85, 95% CI=[-.74,.30], p=.40, effect of motion: b=.21, t=1.6, 95% CI=[-.05,.46], p=.11). Among children, there was no effect of age on lateralization of the language response (effect of age: b=.11, t=.52, 95% CI=[-.32,.53], p=.61, effect of motion: b=.26, t=1.3, 95% CI=[-.17,.69], p=.22). Analyses conducted at a more lenient threshold (p=.01) yielded the same pattern of results.

The lateralization of the response to language is sometimes affected by handedness (Knecht et al., 2000; Szaflarski et al., 2002). We visually inspected the contrast maps and LI values of left-handed participants (n=9; 4 children and 5 adults) and participants without handedness data (n=2 adults). Of the nine left-handed individuals, two individuals appeared to have right-lateralized responses, and three individuals appeared to have responses that were not lateralized. The two individuals without handedness information appeared to have left-lateralized responses. Analyses of the lateralization of the language response that excluded left-handed participants yielded the same pattern of results as those reported in the main text (with left-handed participants included).

There was no effect of age of ASL onset on the lateralization of ToM and language responses; see Supplementary Table 4 for statistics and Supplementary Figures 3 and 4 for visualizations.

Supplementary Note 9

We conducted inter-region correlation (IRC) analyses on the responses within and across group ToM and language brain regions (see Supplementary Table 2 for information about ROIs), using the procedure of a prior study (Richardson, Lisandrelli, Riobueno-Naylor, & Saxe, 2018). Correlated response timecourses across brain regions could reflect similar functional selectivity profiles (two regions activate and deactivate to the same content within the stimulus), information transfer/division of labor between regions (two regions work concurrently to process different aspects of the stimulus), and/or intrinsic network properties (two regions activate and deactivate together regardless of stimulus). For IRC analyses, preprocessed, scaled timecourses were extracted from each voxel per ROI. The five PCA-based noise regressors and motion artifact timepoint regressors (included as nuisance regressors in the story task) were regressed from these timecourses, and the residual timecourses were high-pass filtered with a cut-off of 100 seconds. Timecourses from voxels within an ROI were averaged, creating one timecourse per ROI, and artifact timepoints were subsequently NaNed. Each ROI timecourse was correlated with every other ROI timecourse, per subject, and these correlation values were Fisher z-transformed. Within-ToM and within-Lang network correlations were calculated as the average correlation value between brain regions within each network. Similarly, across-ToM-Lang correlations were calculated as the average correlation value between ToM and Language brain regions. In order to test if different brain networks (ToM-Lang) were functionally dissociated, we used t-tests to compare within- versus across-network correlations. Because of paradigm differences (in the non-sign stimuli) between children and adults, IRC analyses were conducted in each age group separately.

Among children and adults, responses of brain regions within both ToM and Language networks were positively correlated (M(SE) within-ToM correlations: children: .19(.03), adults: .36(.03); M(SE) within-Language correlations: children: .25(.03), adults: .31(.02)). Response timecourses in language and ToM brain regions were also significantly positively correlated (across-network) during the story task, in both age groups (M(SE) across-ToM-Lang correlations: children: .15(.02); adults: .17(.02); t-test against zero (mu=0): children: $t(23)=7.2$, 95% CI=[.11,.19], $p=2.6\times10^{-7}$; adults: $t(35)=10.5$, 95% CI=[.14,21], $p=2.1\times10^{-12}$). Language brain regions were significantly more correlated with other regions within the language network, relative to regions in the ToM network, in both age groups (within vs. across-network correlations: adults: $t(35)=8.7$, 95% CI=[.11,.17], $p=2.7\times10^{-10}$; children: $t(23)=4.4$, 95% CI=[.05,.15], $p=.0002$). Responses in ToM brain regions were similarly significantly more correlated with other regions within their own network, relative to regions in the language network in adults (within vs. across-network correlations: adults: $t(35)=7.1$, 95% CI=[.14,.24], $p=3.2\times10^{-8}$), but this effect was not significant among children ($t(23)=1.7$, 95% CI=[-.01,.09], $p=.11$). Among children, there was no significant correlation between inter-region correlations and age (all bs <.19, ts<.9, ps>.3).

There was no effect of age of ASL onset on the inter-region correlations of ToM and language responses; see Supplementary Table 4 for statistics and Supplementary Figure 4 for visualizations in children.

Supplementary Note 10
We measured neural responses during a non-linguistic movie-viewing task. The movie includes scenes ("events") that highlight the mental states and physical sensations of the characters, which drive responses in ToM brain regions and the "extended Pain Matrix"[59,80], respectively. In a prior study, the "functional maturity" (i.e., similarity to an average adult timecourse) of ToM brain regions increased with age from 3 – 12 years[80]. We tested whether the functional maturity of each participant's response timecourse varied as a function of the age at which they were first exposed to ASL. We calculated the Pearson correlation between each participant's ToM timecourse (averaged across ToM group ROIs, TRs 11:155) and an average adult timecourse derived from the prior study[80]. Functional maturity as measured in this movie is not correlated with selectivity (in RTPJ or in the overall ToM network; Supplementary Figure 7). We therefore investigated functional maturity during movie-viewing as a complementary measure of ToM brain region development.

We additionally tested for differences in response magnitude in ToM regions to three events in the movie. In the previous study, response magnitude to two of these events increased with age in three to twelve year old children (events T01 and T02). In addition, response magnitude to the third event (event T04) positively correlated with performance on a linguistic ToM behavioral battery, controlling for age and motion (and correcting for multiple comparisons)[80]. Regressions on the response magnitude to the three ToM events during the movie task included data from three events (T01, T02, T04) and tested for a significant effect (and interaction) of event.

Finally, we conducted Inter-Region Correlation (IRC) analyses on response timecourses from ToM brain regions and the extended "Pain Matrix" (see Supplementary Table 2 for group ROI information). A prior study found that responses within the ToM and Pain networks become increasingly correlated within-network, and increasingly anti-correlated across-network, during childhood (Richardson et al., 2018). We conducted IRC analyses on the same ToM and pain group regions of interest used in the prior study (using TRs 11:155), using an identical analysis procedure. This procedure was also used to measure IRCs during the ASL story task (see SI Section 8).

First, A whole-brain random effects analysis confirmed that ToM brain regions were recruited for the Mental > Pain event contrast. There were no voxels in which activation to this contrast varied as a function of age of ASL onset (Supplementary Figure 8).

Response timecourses to the movie were highly correlated with an independent average adult response timecourse (M(SE) *r*-value: children: .39(.03), adults: .29 (.04)). Possibly due to an under sampling of young delayed signers in this task (see Supplementary Figure 9 & Supplementary Figure 10), among children, there was a significant ASL-onset-by-age interaction (b=-.73, t=-2.1, 95% CI=[-1.4,.03], p=.04) such that the effect of age on "functional maturity" was smaller in children who experienced a longer delay before exposure to ASL, but there was no main effect of age of ASL onset (age of ASL onset: b=.42, t=1.5, 95% CI=[-.16,1.0], p=.14; for full statistics, see Supplementary Table 5). In a regression with the interaction term removed, age but not age of ASL onset predicted functional maturity (Supplementary Table 5). There was no effect of age of ASL onset on functional maturity in the full sample or in adults (Supplementary Table 5).

There was no difference in response magnitude of the ToM network during ToM events as a function of age of ASL onset (for statistics see Supplementary Table 5; for visualization in children see Supplementary Figure 9 and Supplementary Figure 10). The same results were obtained when excluding a single outlier value (response to event T04: -5.32, measured in a native signing child).

The results of the planned fMRI analyses provide evidence for reduced selectivity in RTPJ among children with delayed exposure to ASL during the story task, but no neural differences between native and delayed signers during the movie task. One difference between the analyses of these two tasks is that the story task analyses focused on responses in individual ROIs, whereas the movie analyses used the average response across multiple ToM brain regions (bilateral TPJ, precuneus, D/M/VMPFC group ROIs). We analyzed functional maturity and response magnitude during the movie-viewing task in the same individual RTPJ ROIs used for the story task analyses. There was no effect of age of ASL onset on functional maturity or on the magnitude of response to ToM events in RTPJ among children, adults, or in the full sample (Supplementary Table 5 and Supplementary Figure 10). The same pattern of results was obtained in analyses of group RTPJ ROIs.

Inter-region correlations within the ToM network were significantly higher than across ToM-Pain network correlations in children ($t(53.7)=12.3$, 95% CI=[.33,.46], $p<2.2\times10^{-16}$) and in adults ($t(55.3)=14.8$, 95% CI=[.42,.56], $p<2.2\times10^{-16}$), and, critically, similarly high regardless of age ASL onset in children and adults (**children:** age of ASL onset: b=.17, t=1.1, 95% CI=[-.13,.47], p=.26, age: b=.60, t=4.1, 95% CI=[.30,.90], p=.0004, motion: b=-.25, t=-1.8, 95% CI=[-.54,.03], p=.08; **adults:** age of ASL onset: b=.23, t=.80, 95% CI=[-.36,.81], p=.43, motion: b=-.31, t=-1.1, 95% CI=[-.89,.27], p=.29). In the full sample, within-ToM correlations were *higher* in individuals with a longer delay before ASL exposure (age of ASL onset: b=.34, t=2.0, 95% CI=[.005,.67], p=.047, age group: b=.59, t=2.3, 95% CI=[.08,1.1], p=.02, motion: b=-.39, t=-2.4, 95% CI=[-.72,-.06], p=.02). Across-ToM-Pain network correlations did not differ based on age of ASL onset among children (age of ASL onset: b=.24, t=1.2, 95% CI=[-.17,.65], p=.23, age: b=-.31, t=-1.5, 95% CI=[-.72,.10], p=.14, motion: b=.22, t=1.1, 95% CI=[-.17,.61], p=.26), adults (b=-.41, t=-1.6, 95% CI=[-.94,.12], p=.12, motion: b=.68, t=2.7, 95% CI=[.15,1.21], p=.01), or in the full sample (age of ASL onset: b=-.21, t=-1.2, 95% CI=[-.55,.14], p=.24, age group: b=-.20, t=-.78, 95% CI=[-.73,.32], p=.44, motion: b=.46, t=2.7, 95% CI=[.12,.80], p=.009).

In contrast to the story task, age of ASL onset did not affect functional maturity of the RTPJ as measured in the non-linguistic movie task. If language experience affects development of ToM concepts per se, why are these effects observable only in response to linguistic narratives? Because there is no prior evidence about development of ToM brain regions in d/Deaf children, we tentatively suggest that the linguistic task may have provided a more direct and/or sensitive measure of RTPJ selectivity than the non-linguistic task. In support of this speculation, selectivity measured in the story task and functional maturity measured in the movie task were uncorrelated in a prior dataset of neurotypical hearing children (Supplementary Figure 7). Unlike the linguistic and minimally linguistic behavioral tasks, the neuroimaging story and movie tasks were not designed or selected to be conceptually analogous. They both evoke mental states, including mistaken beliefs and changing emotions. However, the control conditions differ across the two experiments; the Social stories describe the enduring relationships of characters and their

physical appearances and states, whereas the control events in the movie depict physical pain and bodily transformations. Individuals with delayed access to ASL may still be refining preferential responses that distinguish between mental and social content, despite having developed preferential responses between other conceptual categories relevant for ToM (i.e., minds vs. bodies). It is difficult to test this hypothesis directly with the naturalistic movie stimulus used here because moments that highlight mental states generally also highlight other social aspects of the characters. Of course, it is also possible that we simply did not have the sample size necessary to detect the effect of ASL onset in the non-linguistic fMRI task. Subsequent research is necessary to test the selectivity of RTPJ for mental versus other social information in non-linguistic tasks, and to measure RTPJ responses in conceptually analogous linguistic and non-linguistic tasks.

Supplementary Note 11

Among children, we tested for significant correlations between neural measures and performance on the linguistic and minimally linguistic ToM tasks. We did not find evidence for a correlation between any of the neural measures from the ASL story task and ToM performance (**Response selectivity**: ToM_L: b=.18, t=.97, 95% CI=[-.19,.55], p=.35, ROI: b=-.10, t=-.34, 95% CI=[-.70,.50], p=.74, motion: b=.14, t=.78, 95% CI=[-.22,.51], p=.45; ToM_{ML}: b=.29, t=1.8, 95% CI=[-.03,.60], p=.08, ROI: b=-.03, t=-.12, 95% CI=[-.61,.54], p=.91, motion: b=.29, t=1.9, 95% CI=[-.02,.60], p=.08; **Response lateralization**: ToM_L: b=.07, t=.38, 95% CI=[-.34,.49], p=.71, motion: b=-.17, t=-.80, 95% CI=[-.62,.28], p=.43; ToM_{ML}: b=.21, t=.99, 95% CI=[-.23,.65], p=.33, motion: b=-.16, t=-.75, 95% CI=[-.60,.28], p=.46; **Inter-Region Correlations**: ToM_L: wi-ToM: b=.09, t=.31, 95% CI=[-.53,.72], p=.76, wi-Lang: b=-.21, t=-.51, 95% CI=[-1.1,.67], p=.62, ac-ToM-Lang: b=.27, t=.69, 95% CI=[-.55,1.1], p=.50, motion: b=.09, t=.34, 95% CI=[-.47,.65], p=.74; ToM_{ML}: wi-ToM: b=.09, t=.32, 95% CI=[-.47,.64], p=.75, wi-Lang: b=.05, t=.13, 95% CI=[-.69,.78], p=.90, ac-ToM-Lang: b=-.20, t=-.57, 95% CI=[-.94,.53], p=.58, motion: b=-.07, t=-.28, 95% CI=[-.58,.44], p=.79).

We similarly did not find robust evidence for a correlation between neural measures from the non-linguistic movie-viewing task and ToM performance. Across all children, within-ToM inter-region correlation and functional maturity measures were significantly positively correlated with performance on the minimally linguistic ToM task (**wi-ToM IRC:** ToM_{ML}: b=.50, t=3.1, 95% CI=[.17,.83], p=.005; motion: b=-.38, t=-2.3, 95% CI=[-.71,-.05], p=.03; **FM:** ToM_{ML}: b=.34, t=2.1, 95% CI=[.08,.68], p=.045; motion: b=-.49, t=-3.02, 95% CI=[-.83,-.16], p=.006), but neither of these correlations remained significant when additionally controlling for age (**wi-ToM IRC:** ToM_{ML}: b=.07, t=.33, 95% CI=[-.34,.48], p=.74, age: b=.61, t=3.1, 95% CI=[.20,1.0], p=.006, motion: b=-.25, t=-1.7, 95% CI=[-.55,.05], p=.10; **FM:** ToM_{ML}: b=.04, t=.16, 95% CI=[-.42,.49], p=.87, age: b=.44, t=2.0, 95% CI=[-.02,.90], p=.06, motion: b=-.40, t=-2.5, 95% CI=[-.74,-.07], p=.02). These two measures were not correlated with performance on the linguistic ToM task (**wi-ToM IRC:** ToM_L: b=.31, t=1.4, 95% CI=[-.14,.76], p=.17; motion: b=-.25, t=-1.2, 95% CI=[-.69,.20], p=.26; **FM:** ToM_L: b=.10, t=.52, 95% CI=[-.30,.50], p=.61; motion: b=-.61, t=-3.2, 95% CI=[-1.0,-.21], p=.005). The across-ToM-Pain inter-region correlation measure and the response magnitude to ToM events were not correlated with performance on either ToM task (**across-ToM-Pain:** ToM_L: b=-.16, t=-.63, 95% CI=[-.69,.37], p=.54; ToM_{ML}: b=-.20, t=-1.1,

95% CI=[-.59,.19], p=.30; **Response magnitude**: ToM_L: b=.12, t=1, p=.32; ToM_{ML}: b=.20, t=2, p=.06).

Supplementary References

Desmond, J. E., Sum, J. M., Wagner, A. D., Demb, J. B., Shear, P. K., Glover, G. H., et al. (1995). Functional MRI measurement of language lateralization in Wada-tested patients. *Brain*, *118*(6), 1411–1419.

Dufour, N., Redcay, E., Young, L., Mavros, P. L., Moran, J. M., Triantafyllou, C., et al. (2013). Similar Brain Activation during False Belief Tasks in a Large Sample of Adults with and without Autism. *PLoS ONE*, *8*(9), e75468. http://doi.org/10.1371/journal.pone.0075468

Enns, C. J., & Herman, R. C. (2011). Adapting the assessing british sign language development: Receptive skills test into American sign language. *Journal of Deaf Studies and Deaf Education*, *16*(3), 362–374.

Knecht, S., Dräger, B., Deppe, M., Bobe, L., Lohmann, H., Flöel, A., et al. (2000). Handedness and hemispheric language dominance in healthy humans. *Brain*, *123*(12), 2512–2518.

Reher, K., & Sohn, P. (2009). *Partly Cloudy* [Motion Picture]. Pixar Animation Studios and Walt Disney Pictures.

Richardson, H., Lisandrelli, G., Riobueno-Naylor, A., & Saxe, R. (2018). Development of the social brain from age three to twelve years. *Nature Communications*, *9*(1), 1027.

Szaflarski, J. P., Binder, J. R., Possing, E. T., McKiernan, K. A., Ward, B. D., & Hammeke, T. A. (2002). Language lateralization in left-handed and ambidextrous people fMRI data. *Neurology*, *59*(2), 238–244.

Supplementary Figure 1

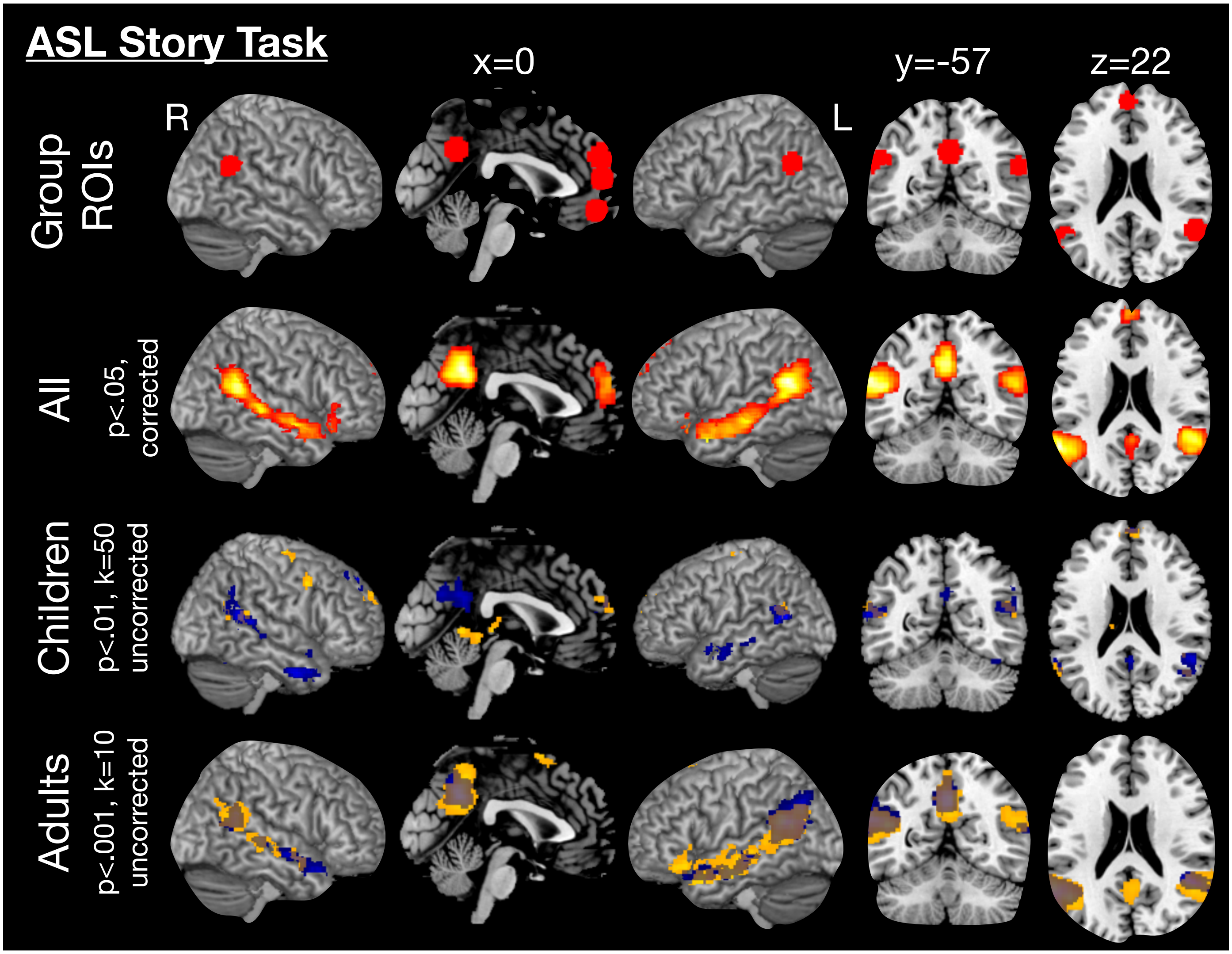

Supplementary Figure 1. ASL Story Task: Group ToM ROIs and Random Effects Analysis of ToM Response. Group ROIs are shown in red (top row). Whole-brain analyses were used to examine the main contrast of interest (Mental > Physical, one-sample t-tests) in the whole sample ("All"; second row, hot colors, n=60), and per group (native (blue) and delayed (orange) signers, in children (n=8 delayed signers, n=16 native signers) and adults (n=16 delayed signers, n=20 native signers)). "Corrected" indicates clusters survived correction for multiple comparisons with permutation analyses (SnPM, p<.05); uncorrected analyses were not corrected for multiple comparisons (this more lenient procedure enabled visualization of all groups). Subtraction analyses (native > delayed signers) revealed no significant clusters in either age group.

Supplementary Figure 2

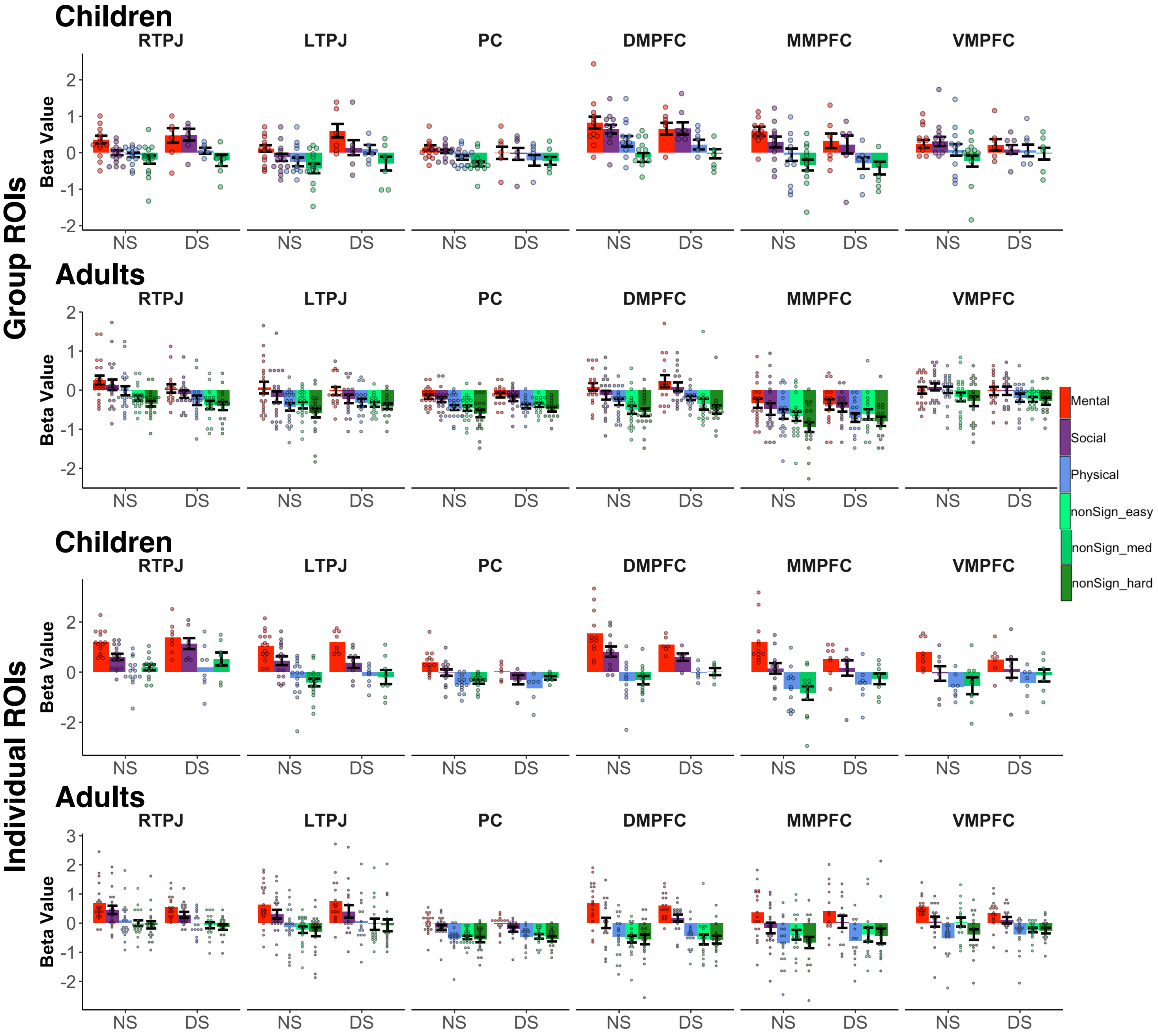

Supplementary Figure 2. ASL Story Task: Average beta values per condition and ToM ROI. Bar plots show average beta values per condition +/- SEM, for each ROI, by age of ASL onset group (native signers (NS) and delayed signers (DS)) and age (children and adults) in group ROIs (top) and individually-defined ROIs (bottom); individual dots refer to individual participants. For group ROIs, all bar plots show data for n=16 NS children, n=8 DS children, n=20 NS adults, and n=16 DS adults. For individual ROIs, bar plots show data for: RTPJ: n=15 NS children, n=8 DS children, n=20 NS adults, n=16 DS adults; DMPFC: n=12 NS children, n=6 DS children, n=19 NS adults, n=16 DS adults; LTPJ: n=16 NS children, n=8 DS children, n=20 NS adults, n=16 DS adults; MMPFC: n=12 NS children, n=7 DS children, n=18 NS adults, n=14 DS adults; VMPFC: n=8 NS children, n=7 DS children, n=16 NS adults, n=16 DS adults; PC: n=14 NS children, n=6 DS children, n=20 NS adults, n=15 DS adults. Because individual ROIs were defined based on the Mental and Physical conditions, these conditions are plotted for visualization purposes only and do not have error bars. All statistical tests found a similar pattern of results in individual and group ROIs (Supplementary Note 4, Supplementary Table 3). Note that overall lower response magnitudes in adults are consistent with prior unpublished data from adults who completed an English version of this (child-directed) story task during fMRI. Source data are provided as a Source Data file and at https://osf.io/kyu3f/.

Supplementary Figure 3

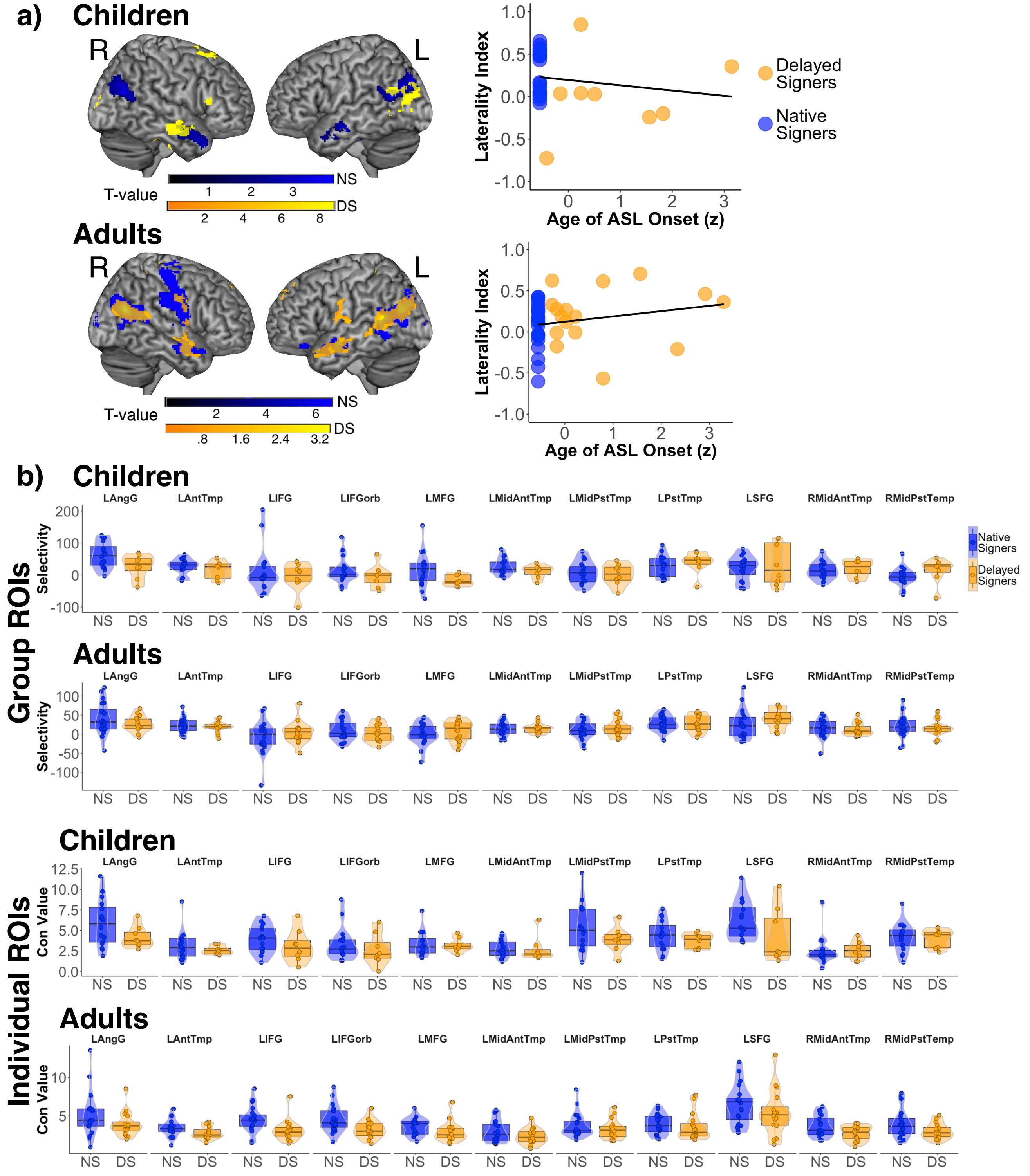

Supplementary Figure 3. Language Responses to ASL Story Task. a) Left: Whole-brain random effects analysis for the Physical > Non-Sign contrast, corrected for multiple comparisons using permutation analyses (p<.05, SnPM). Delayed signing children (n=8) and adults (n=16) are shown in orange; native signing children (n=16) and adults (n=20) are shown in blue. There were no voxels in which activity was significantly predicted by age of ASL onset. Right: Scatterplots show laterality index (y-axis, using p<.001 threshold) by age of ASL onset (x-axis); age of ASL onset did not predict the laterality of the language response among children (b=-.14, t=-.67, p=.51; linear regression controlling for age and motion) or adults (b=.11, t=.40, p=.70; linear regression controlling for motion); see Supplementary Table 4 and Supplementary Notes 7 and 8. **b)** Violin plots show the selectivity index (Physical-Non-Signs*100) in group ROIs (top) and the Physical > Non-Signs contrast value in individually-defined ROIs (bottom); see Table 2 and Supplementary Note 7. Figure legend continues on the following page.

All adult analyses used the 8 Non-Sign condition (Children: 5 Non-Signs). In all violin plots, the center line reflects the median, the box reflects the inter-quartile range (IQR), and whiskers show the first quartile/third quartile -/+ 1.5*IQR. In all plots, native signers are shown in blue and delayed signers are shown in orange. Source data are provided as a Source Data file and at https://osf.io/kyu3f/.

Supplementary Figure 4

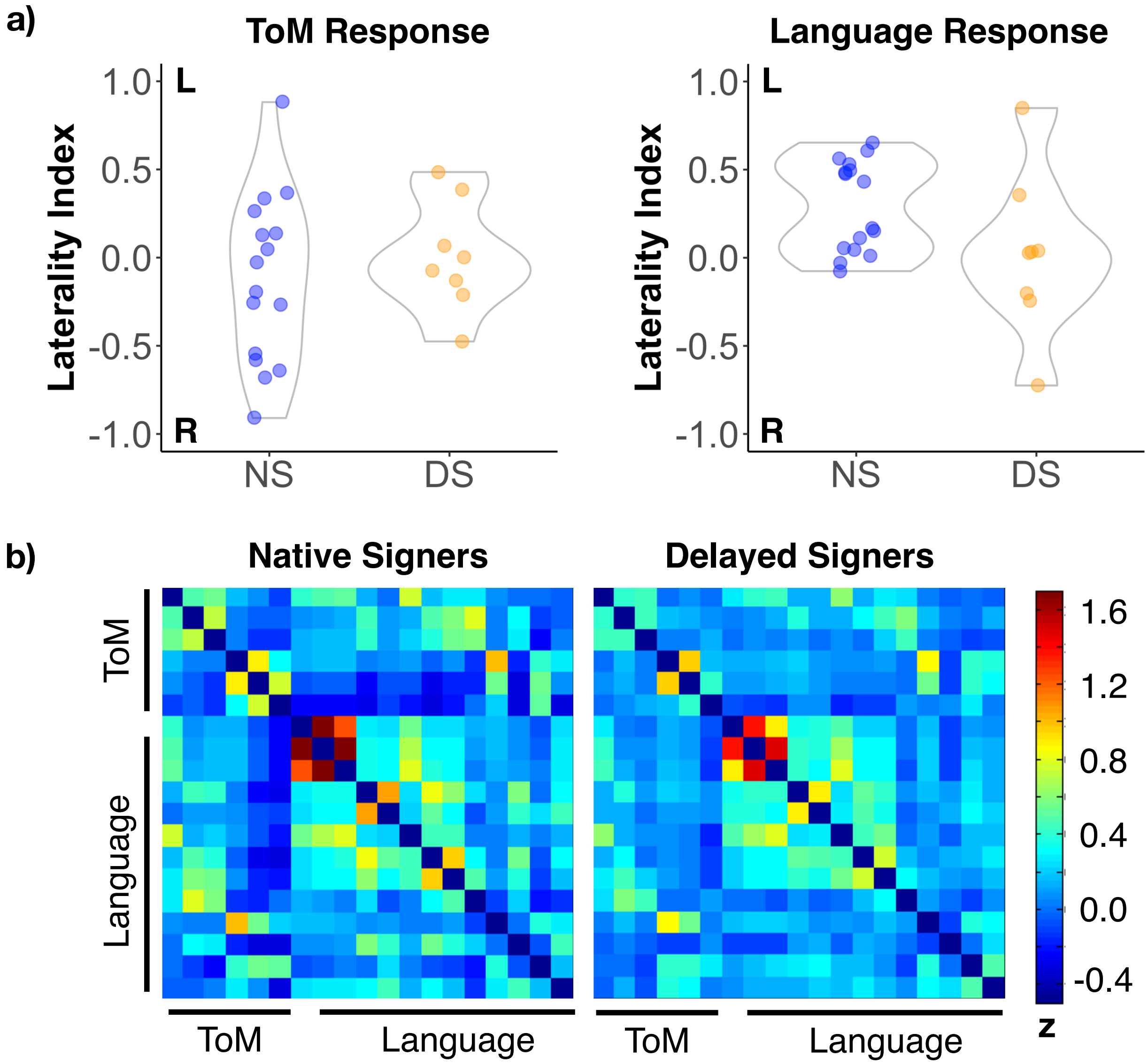

Supplementary Figure 4. Lateralization and Inter-region Correlations in ToM and Language Networks to ASL Story Task. a) Violin plots show the laterality index for native signing (NS; blue/left, n=16) and delayed signing (DS; orange/right, n=8) children in ToM (left) and language (right) cortex. Laterality index was calculated as ($NumVox_L$ - $NumVox_R$)/($NumVox_L$ + $NumVox_R$), where NumVox is number of suprathreshold voxels to the Mental > Physical (ToM) or Physical > Non-Sign (Language) contrast (p<.001; results unchanged at p<.01). Age of ASL onset did not predict ToM (b=.15, t=.68, p=.51) or language (b=-.14, t=-.67, p=.51) response lateralization (linear regressions controlling for age and motion; Supplementary Table 4). Source data are provided as a Source Data file and at https://osf.io/kyu3f/. **b)** Correlation matrices show average z-scored inter-region correlation values across all ToM and language brain regions of interest, for native signing (left, n=16) and delayed signing (right, n=8) children. Regions are in the same order along the x- and y-axes: R/LTPJ, PC, D/M/VMPFC, RSTS, RSTS/R Middle Anterior Temporal lobe (overlap), R/L Middle Anterior Temporal lobe, L Anterior Temporal lobe, R/L Middle Posterior Temporal lobe, L Posterior Temporal lobe, L Angular Gyrus, L Superior Frontal Gyrus, L Middle Frontal Gyrus, L Orbital Inferior Frontal Gyrus, L Inferior Frontal Gyrus).

Supplementary Figure 5

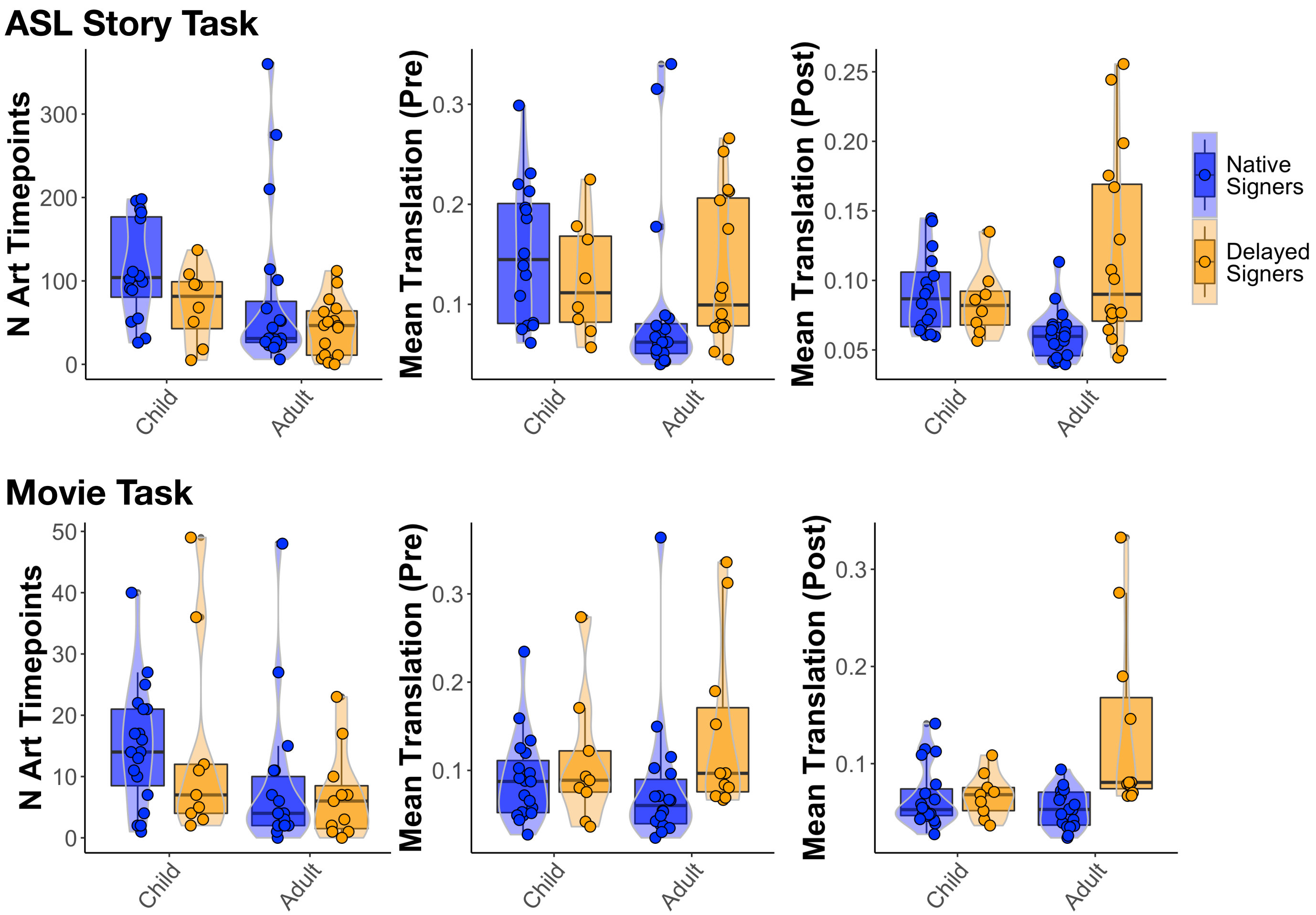

Supplementary Figure 5. Amount of motion in fMRI data. Left: Number of artifact timepoints identified in the timecourse of response (Top: ASL Story task, Bottom: Movie task), by group (ASL Story task: n=8 delayed signing and n=16 native signing children; n=16 delayed signing and n=20 native signing adults; Moving-Viewing task: n=9 delayed signing and n=19 native signing children; n=11 delayed signing and n=18 native signing adults). Artifact timepoints are timepoints in which there is 2mm motion and/or a global signal change greater than three standard deviations from the mean, relative to the previous timepoint. Middle/Right: Mean translation (motion in x, y, z directions) in millimeters per ASL-onset and age group, pre- (middle) and post (right) exclusion of artifact timepoints. In all box plots, the center line reflects the median, the box reflects the inter-quartile range (IQR), and whiskers show the first quartile/third quartile -/+ 1.5*IQR. Source data are provided as a Source Data file and at https://osf.io/kyu3f/.

Supplementary Figure 6

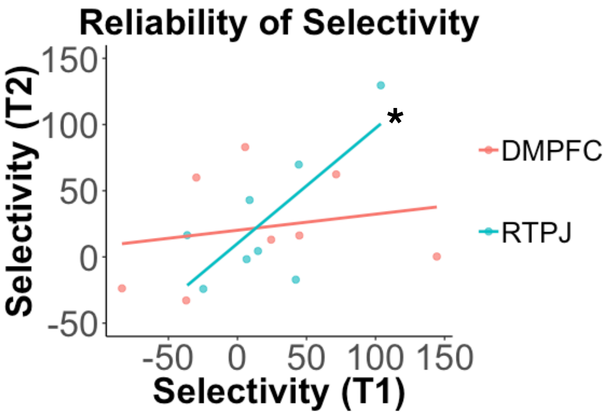

Supplementary Figure 6. Pilot data on the reliability of response selectivity. Scatterplot shows the within-subject correlation between selectivity in RTPJ (blue) and DMPFC (pink) group ROIs, as measured during two analogous but distinct English story tasks in a pilot sample of neurotypical hearing children (n=8; see Supplementary Note 6 for details). Asterisk indicates that response selectivity was reliable in RTPJ ($r(6)$=.75, 95% CI=[.08, .95], p=.03; Pearson's correlation); this correlation was not significant in DMPFC ($r(6)$=.20, 95% CI=[-.58,.79], p=.63). A mixed effect linear regression including both ROIs suggested the selectivity measure was not significantly more reliable in RTPJ than DMPFC (interaction: b=1.02, t=1.9, p=.12). Source data are provided as a Source Data file and at https://osf.io/kyu3f/.

Supplementary Figure 7

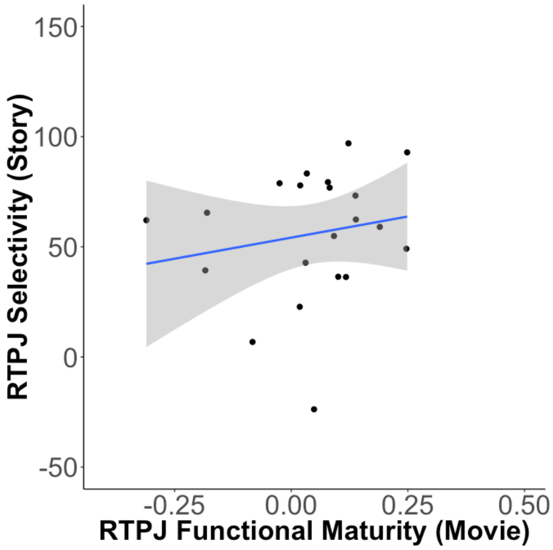

Supplementary Figure 7. Story Task & Movie-Viewing Measures are Uncorrelated in Prior Data. In an independent dataset (n=26 five year old neurotypical hearing children; M(SD)=5.5(.27) years), we used linear regressions to test for a significant correlation between individually-defined RTPJ selectivity, as measured during an English version of the story task (Gweon et al., 2012; see also https://osf.io/wzd8a/), and functional maturity of the RTPJ and ToM network, as measured during the movie-viewing task, controlling for age and average amount of motion across the two tasks. Individual and group regions of interest were defined using the same methods described in the main text. Functional maturity of the RTPJ did not significantly predict RTPJ selectivity (linear regression analyses; effect of functional maturity: b=.13, t=.59, 95% CI = [-.33,.59], p=.56; effect of age: b=.16, t=.72, 95% CI=[-.30,.61], p=.48; effect of motion: b=.42, t=1.8, 95% CI= [-.06,.91], p=.08). The same pattern of results was found in linear regressions that tested for effects of functional maturity of the entire ToM network (b=.39, t=1.8, 95% CI=[-.06,.85], p=.09) and in a group RTPJ ROI (b=.37, t=1.4, 95% CI= [-.20,.94], p=.19) on RTPJ selectivity, controlling for age and motion. Source data are provided as a Source Data file and at https://osf.io/kyu3f/.

Supplementary Figure 8

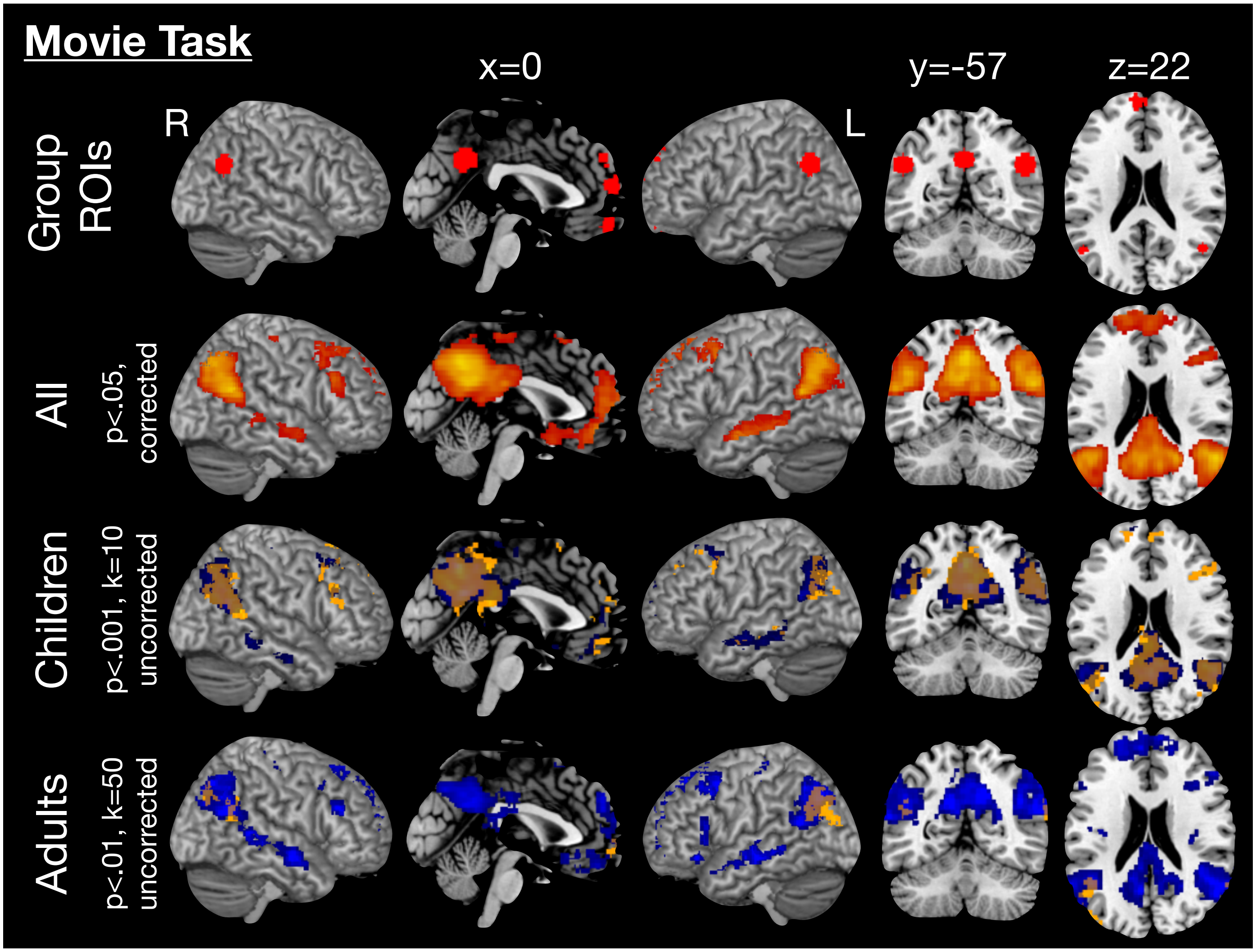

Supplementary Figure 8. Movie Task: Group ToM ROIs and Random Effects Analysis of ToM Response. Group ROIs are shown in red (top row). Whole-brain analyses were used to examine the main contrast of interest (Mental > Pain, one-sample t-tests) in the whole sample ("All", hot colors, n=57), and per signing and age group. Native signers are shown in blue (n=19 children; n=18 adults); delayed signers are shown in orange (n=9 children, n=11 adults). "Corrected" indicates clusters survived correction for multiple comparisons with permutation analyses (SnPM, p<.05); uncorrected analyses were not corrected for multiple comparisons (this more lenient procedure enabled visualization of all groups). Subtraction analyses (native > delayed signers) revealed no significant clusters in either age group.

Supplementary Figure 9

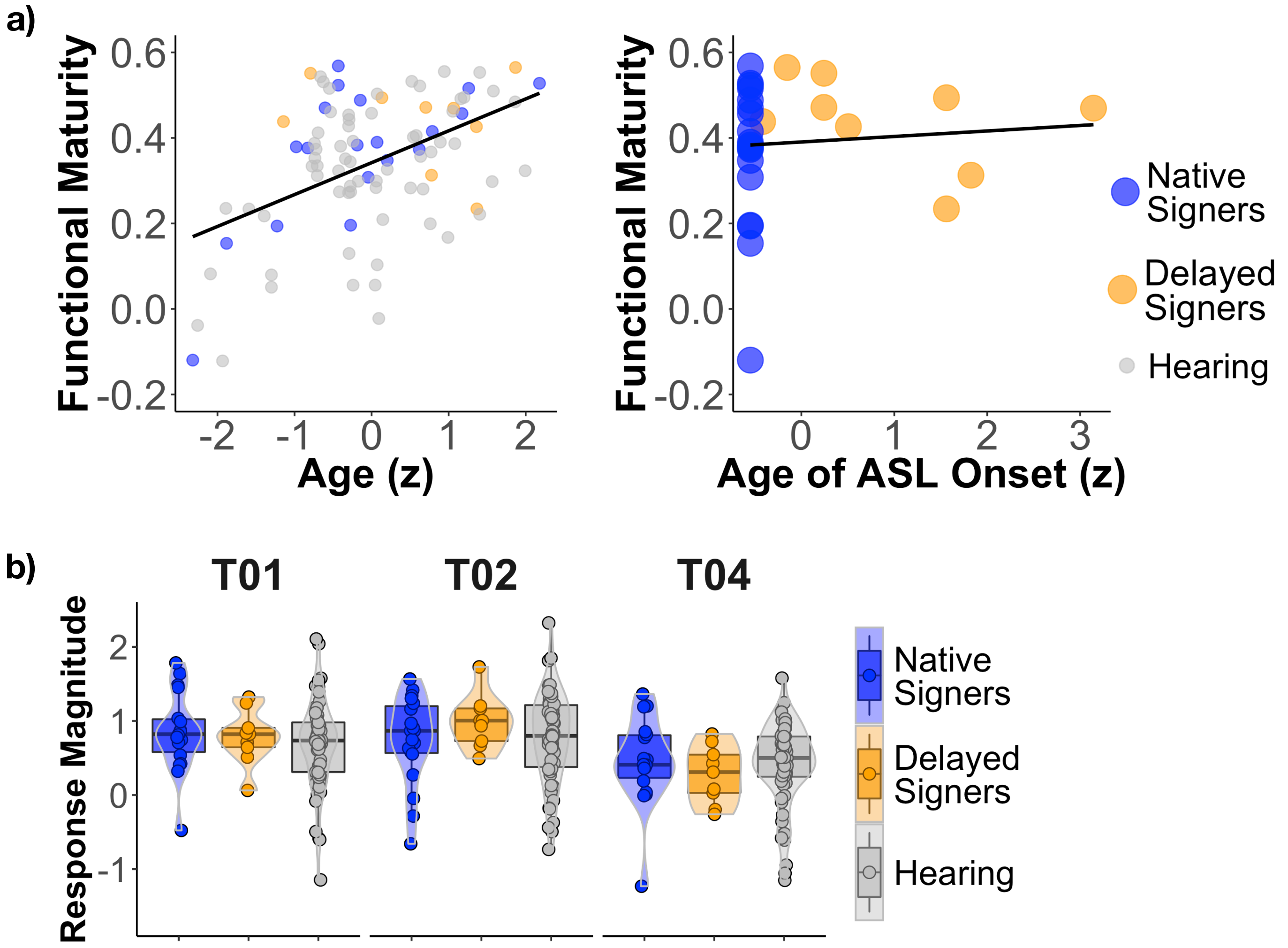

Supplementary Figure 9. Neural Responses to Movie Task. a) Scatterplots show the functional maturity (i.e. similarity to an average adult timecourse, Pearson's *r*; y-axis) in the ToM network by z-scored age (x-axis; left) and by z-scored age of ASL onset (x-axis; right) among child participants. Native signing children (n=19) are shown in blue; delayed signing children (n=9) are shown in orange. Grey dots show data from an age-matched sample of neurotypical hearing children (n=65, 4-12 year olds). **b)** Box plots show the response magnitude in the ToM network in native signing, delayed signing, and hearing children to three ToM events (T01, T02, T04), which previously showed developmental change with age (T01, T02) and ToM score (T04; Richardson et al., 2018). The center line of the box plots reflects the median, the box reflects the inter-quartile range (IQR), and whiskers show the first quartile/third quartile -/+ 1.5*IQR. Z-scores are used on x-axes for de-identification of data. Source data are provided as a Source Data file and at https://osf.io/kyu3f/.

Supplementary Figure 10

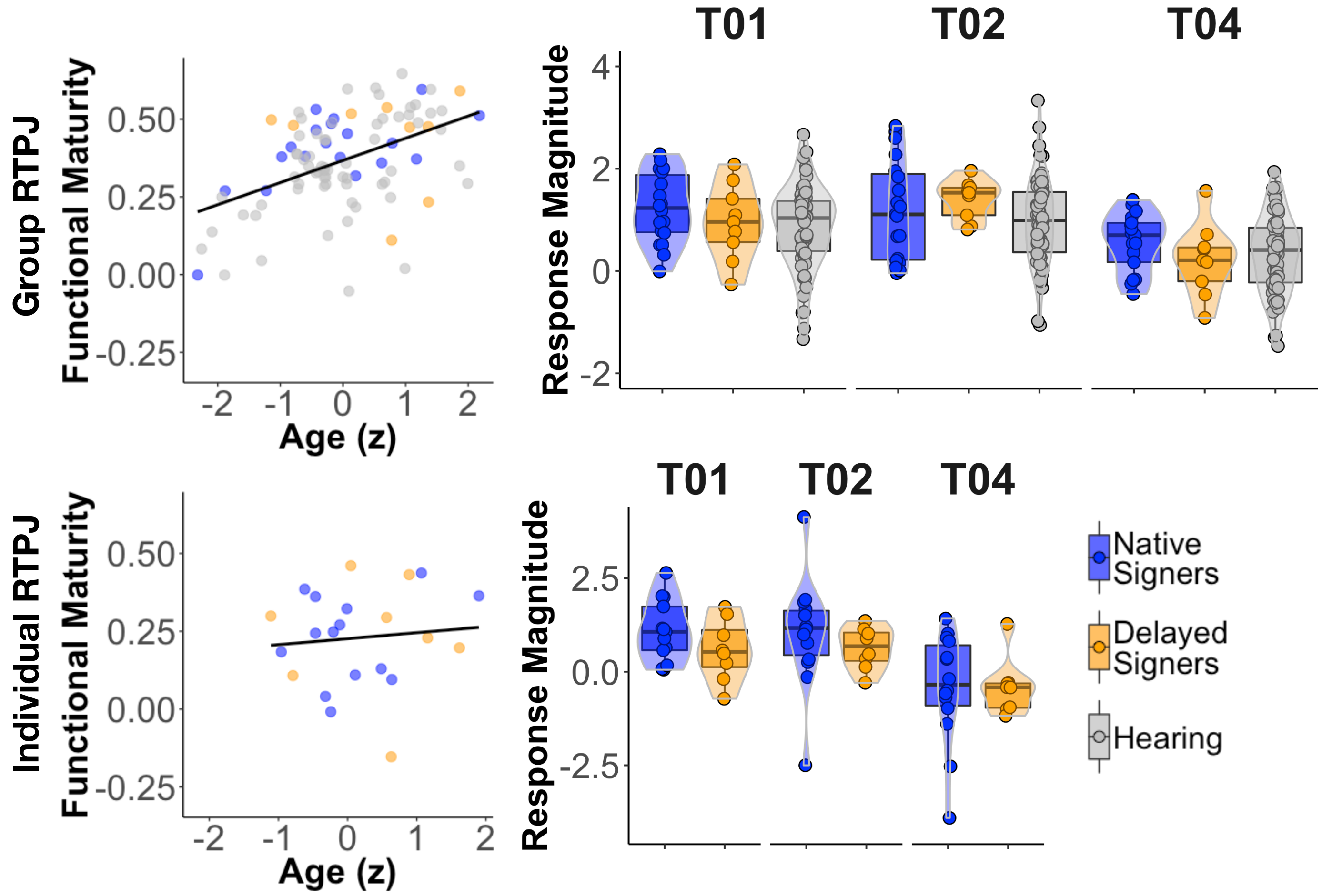

Supplementary Figure 10. RTPJ Responses as Measured by Movie Task. Scatterplots (left) show functional maturity (i.e., similarity to an average adult timecourse, Pearson's *r*) on the y-axis and z-scored age on the x-axis in native signing children (blue, n=19), delayed signing children (orange, n=9), and in an age-matched group of neurotypical hearing participants who completed this fMRI task as part of other studies (grey; n=65 4-12 year olds) in a group RTPJ ROI (top), and in individual RTPJ ROIs defined using the linguistic story task (bottom; current sample only). Individual RTPJ ROIs were not available for hearing participants because they did not also complete the story task. Boxplots (right) show response magnitude in the group RTPJ ROI (top; excluding a single outlier value (response to T04 in native signing child: -5.32)) and in individual RTPJ ROIs (bottom) in native signing (blue), delayed signing (orange), and hearing (grey) children, to three ToM events (T01, T02, T04), which previously showed developmental change with age (T01, T02) and ToM score (T04; Richardson et al., 2018). In all box plots, the center line reflects the median, the box reflects the inter-quartile range (IQR), and whiskers show the first quartile/ third quartile -/+ 1.5*IQR. Z-scores are used on x-axes for de-identification of data. Source data are provided as a Source Data file and at https://osf.io/kyu3f/.

Supplementary Table 1

Subject	Age (Z)	Gender	Handedness	Age of ASL Onset (Z)	Parent Hearing Status	Native vs. Delayed Signer	ASL-Rec	KBIT-Std	CORSI	Flanker	ToM-L	ToM-ML	Num Art (Story)	Mean Trans (Story)	Num Art (Movie)	Mean Trans (Movie)	fMRI Data (Story)	fMRI Data (Movie)
C_01	1.25	F	R	-0.55	DOD	NS	0.975	115	NA	NA	0.884	0.862	31	0.099	NA	NA	1	0
C_02	-1.09	M	L	-0.42	DOH	DS	0.825	NA	NA	NA	0.529	0.615	51	0.090	2	0.0754	1	1
C_03	-0.22	M	R	-0.55	CODA	NS	0.750	116	NA	NA	0.889	0.667	106	0.060	1	0.0273	1	1
C_04	-1.75	M	R	-0.55	CODA	NS	0.500	104	NA	NA	NA	0.400	NA	NA	21	0.0384	0	1
C_05	1.01	F	R	-0.55	CODA	NS	0.875	120	5	0.05118	0.791	0.852	99	0.068	22	0.0416	1	1
C_06	-0.30	F	R	-0.55	CODA	NS	0.650	116	5	0.22357	0.867	0.833	106	0.145	4	0.1125	1	1
C_07	0.65	M	R	1.82	DOH	DS	0.825	102	NA	-0.058	0.700	0.833	137	0.099	4	0.0901	1	1
C_08	-0.24	F	R	-0.02	DOH	DS	0.825	115	5	0.0842	0.841	0.800	NA	NA	NA	NA	0	0
C_09	-1.16	F	R	-0.55	CODA	NS	0.400	86	NA	-0.2139	NA	0.621	NA	NA	2	0.0484	0	1
C_10	0.13	F	R	-0.55	CODA	NS	0.675	119	5	-0.01	0.833	0.842	196	0.093	17	0.0786	1	1
C_11	-0.18	M	R	-0.55	DOD	NS	NA	143	5	0.04325	NA	0.767	102	0.114	27	0.0583	1	1
C_12	-0.94	M	R	-0.55	DOD	NS	0.550	109	5	-0.0579	NA	0.600	198	0.076	2	0.0526	1	1
C_13	0.51	M	L	-0.55	DOD	NS	NA	122	5	-0.0453	NA	0.767	186	0.143	16	0.1149	1	1
C_14	-0.60	M	R	-0.55	DOD	NS	0.425	87	4	0.04396	0.766	0.667	111	0.103	21	0.1090	1	1
C_15	0.66	M	R	-0.55	DOD	NS	0.675	128	5	0.04603	0.936	0.733	51	0.125	17	0.0694	1	1
C_16	1.92	M	R	-0.55	DOD	NS	0.875	117	5	0.06353	0.938	0.931	91	0.072	14	0.0504	1	1
C_17	-0.44	F	R	-0.55	DOD	NS	0.875	132	4	NA	0.848	0.867	89	0.061	14	0.0469	1	1
C_18	-0.80	M	R	-0.55	DOD	NS	0.800	124	4	0.10282	0.818	0.688	NA	NA	25	0.0632	0	1
C_19	-0.09	F	R	-0.55	DOD	NS	0.900	111	4	0.0016	0.979	0.867	175	0.090	13	0.0463	1	1
C_20	-2.15	F	R	-0.55	DOD	NS	0.556	101	NA	NA	0.474	0.750	NA	NA	40	0.1412	0	1
C_21	-1.46	M	R	-0.55	DOD	NS	0.525	96	3	NA	0.700	0.533	NA	NA	NA	NA	0	0
C_22	-0.44	M	R	-0.55	DOD	NS	0.775	130	5	0.0969	0.795	0.867	182	0.083	10	0.0478	1	1
C_23	0.01	F	L	-0.55	DOD	NS	0.875	124	5	0.01753	0.894	0.867	55	0.064	11	0.0546	1	1
C_24	1.09	F	L	-0.55	DOD	NS	0.650	129	6	0.02944	0.939	0.800	26	0.061	7	0.0429	1	1
C_25	-0.89	F	R	0.42	DOH	DS	0.800	135	NA	NA	0.750	0.667	NA	NA	NA	NA	0	0
C_26	1.18	M	R	0.51	DOH	DS	0.825	110	5	0.15655	0.911	0.900	95	0.135	12	0.1086	1	1
C_27	1.64	M	R	-0.15	DOH	DS	0.775	118	NA	0.09075	0.796	0.867	18	0.070	3	0.0421	1	1
C_28	0.91	M	R	3.14	DOH	DS	0.725	95	5	0.06032	0.660	0.767	5	0.057	7	0.0681	1	1
C_29	-0.77	F	R	0.24	DOH	DS	0.800	104	5	-0.0375	0.729	0.667	108	0.086	36	0.0516	1	1
C_30	0.07	F	R	1.56	DOH	DS	0.650	111	4	0.0755	0.959	0.867	68	0.063	5	0.0363	1	1
C_31	0.73	M	R	2.62	DOH	DS	0.650	60	4	NA	0.422	0.500	NA	NA	NA	NA	0	0
C_32	0.58	F	R	0.24	DOH	DS	0.825	142	6	0.03619	0.875	0.967	96	0.078	11	0.0709	1	1
C_33	1.18	M	R	1.56	DOH	DS	0.900	132	5	-0.0009	0.804	0.900	NA	NA	49	0.0609	0	1
A_01	-1.0001	F	R	-0.55	DOD	NS							52	0.053	NA	NA	1	0
A_02	0.47119	M	R	-0.17	DOH	DS							7	0.108	NA	NA	1	0
A_03	-0.1346	F	R	-0.17	DOH	DS							112	0.129	NA	NA	1	0
A_04	-0.5673	M	R	-0.26	DOH	DS							11	0.079	NA	NA	1	0
A_05	-1.0001	F	R	-0.07	DOH	DS							11	0.045	NA	NA	1	0
A_06	2.63481	M	R	1.57	DOH	DS (LS)							47	0.199	3	0.1460	1	1
A_07	-1.1732	F	R	-0.55	DOH	NS							210	0.087	7	0.0743	1	1
A_08	-0.1346	M	L	-0.55	DOH	NS							31	0.041	4	0.0392	1	1
A_09	0.03846	F	R	-0.55	CODA	NS							30	0.057	2	0.0351	1	1
A_10	-0.5673	M	L	-0.55	DOD	NS							23	0.068	15	0.0722	1	1
A_11	-0.2212	F	R	-0.26	DOH	DS							67	0.073	17	0.0807	1	1
A_12	-0.3943	M	L	0.22	DOH	DS							52	0.050	7	0.0668	1	1
A_13	-0.827	F	R	-0.55	DOH	NS							114	0.066	NA	NA	1	0
A_14	-0.3943	M	R	0.02	DOH	DS							98	0.058	6	0.0791	1	1
A_15	-1.0001	F	R	-0.55	CODA	NS							101	0.060	0	0.0661	1	1
A_16	-0.1346	M	R	-0.55	DOD	NS							53	0.067	1	0.0525	1	1
A_17	-0.9135	M	R	-0.55	CODA	NS							275	0.068	48	0.0422	1	1
A_18	0.99046	M	L	0.79	DOH	DS							46	0.101	23	0.0783	1	1
A_19	0.55773	F	R	-0.55	DOD	NS							21	0.042	2	0.0237	1	1
A_20	1.16355	F	R	-0.55	CODA	NS							27	0.063	11	0.0730	1	1
A_21	-0.9135	M	R	-0.55	DOD	NS							31	0.064	4	0.0625	1	1
A_22	-0.7404	M	R	-0.55	DOD	NS							360	0.113	27	0.0940	1	1
A_23	-1.0866	F	R	-0.55	DOD	NS							44	0.060	4	0.0533	1	1
A_24	1.68282	F	R	-0.55	DOD	NS							27	0.042	11	0.0359	1	1
A_25	-0.3943	M	R	-0.17	DOH	DS							25	0.076	7	0.0701	1	1
A_26	2.46172	M	R	2.91	DOH	DS (LS)							78	0.167	NA	NA	1	0
A_27	-0.2212	F	L	-0.55	DOD	NS							24	0.040	2	0.0346	1	1
A_28	-0.827	M	R	-0.55	CODA	NS							67	0.046	3	0.0258	1	1
A_29	-0.827	F	R	-0.55	CODA	NS							27	0.044	2	0.0480	1	1
A_30	0.2981	F	R	-0.55	CODA	NS							6	0.054	4	0.0578	1	1
A_31	0.99046	M	R	0.02	DOH	DS							2	0.077	1	0.0807	1	1
A_32	0.55773	M	R	3.30	DOH	DS (LS)							0	0.175	0	0.1898	1	1
A_33	1.25009	M	R	2.34	DOH	DS (LS)							51	0.256	10	0.2758	1	1
A_34	0.81737	M	NA	0.79	DOH	DS							44	0.244	2	0.3327	1	1
A_35	-1.0866	F	NA	0.22	DOH	DS							63	0.064	1	0.0670	1	1
A_36	0.64428	F	R	-0.55	DOD	NS							20	0.075	6	0.0783	1	1

Supplementary Table 1. Participant Demographics. Z-scored age and age of ASL onset variables were calculated per age group for de-identification of data. Parent hearing status indicates participants who are deaf children of deaf adults (DoD), hearing children of deaf adults (CoDA), and deaf children of hearing adults (DoH). Native signers (NS) were exposed to ASL from birth; delayed signers received exposure to ASL after a delay of up to 7 years (DS) or longer (late signers, DS (LS)). ASL-Rec indicates score on a test of receptive vocabulary (ASL-RST, KBIT-Std indicates standardized score on the non-verbal IQ subtest, CORSI indicates blocks passed on computerized working memory task; Flanker indicates reaction time cost (s) for incongruent relative to congruent trials. ToM-L is proportion correct on the linguistic ToM task; ToM-ML is proportion correct on the min-linguistic ToM task. NumArt (Story/Movie) indicates the number of artifact timepoints during the story and movie fMRI tasks, respectively; MeanTrans (Story/Movie) indicates the mean amount of translation (movement in x-, y-, and z- axes) between timepoints during the story and movie fMRI tasks, respectively. fMRI Data (Story/ Movie) indicates whether a participant contributed usable data to the story and movie tasks, respectively.

Supplementary Table 2

INDIVIDUAL Regions of Interest						
Age Group	ROI	ASL Group	# Identifed	Peak Coordinate	M(SD) N Voxels	M(SD) Peak T
Child	RTPJ	NS	15/16	[53 -47 18]	161 (105)	6.9 (2.3)
		DS	8/8	[57 -49 21]	133 (103)	6.6 (1.7)
	DMPFC	NS	12/16	[-2 54 30]	130 (90)	6.7 (1.4)
		DS	6/8	[4 56 28]	156 (90)	6.3 (1.3)
	LTPJ	NS	16/16	[-53 -58 20]	138 (99)	6.5 (1.9)
		DS	8/8	[-54 -57 29]	124 (74)	7.1 (1.5)
	MMPFC	NS	12/16	[4 54 14]	128 (78)	6.7 (1.3)
		DS	7/8	[-1 58 12]	147 (112)	6.6 (1.9)
	VMPFC	NS	8/16	[2 56 -9]	112 (103)	5.6 (1.4)
		DS	7/8	[-1 45 -11]	82 (76)	5.5 (1.4)
	PC	NS	14/16	[0 -54 33]	170 (119)	6.3 (2.0)
		DS	6/8	[3 -55 37]	117 (127)	5.8 (1.7)
Adult	RTPJ	NS	20/20	[54 -51 20]	179 (91)	8.0 (1.9)
		DS	16/16	[56 -47 19]	179 (99)	7.9 (2.1)
	DMPFC	NS	19/20	[-4 55 32]	88 (54)	6.9 (1.8)
		DS	16/16	[-1 54 30]	116 (88)	7.0 (2.9)
	LTPJ	NS	20/20	[-53 -59 22]	221 (89)	8.9 (1.6)
		DS	16/16	[-52 -57 21]	210 (103)	8.5 (2.4)
	MMPFC	NS	18/20	[-1 58 16]	86 (77)	6.2 (1.9)
		DS	14/16	[3 56 14]	134 (99)	7.3 (2.1)
	VMPFC	NS	16/20	[0 56 -15]	104 (52)	7.3 (2.2)
		DS	16/16	[-1 54 -14]	99 (76)	6.3 (1.8)
	PC	NS	20/20	[-1 -57 39]	203 (105)	7.3 (1.6)
		DS	15/16	[-5 -55 37]	199 (99)	7.7 (2.4)
GROUP Regions of Interest						
STORY	ROI			Center Coordinate	N Voxels	
ToM	RTPJ			[54 -52 23]	463	
	LTPJ			[-52 -58 25]	379	
	PC			[1 -56 34]	498	
	DMPFC			[-1 53 29]	455	
	MMPFC			[1 54 12]	498	
	VMPFC			[1 50 -12]	498	
	RSTS			[55 -10 -16]	172	
Overlap	RSTS/RMidAntTemp			N/A	326	
Language	RMidAntTemp			[55 -14 -13]	210	
	LMidAntTemp			[-55 -18 -13]	536	
	LAntTemp			[-52 2 -18]	515	
	RMidPostTemp			[58 -45 10]	463	
	LMidPostTemp			[-56 -40 10]	515	
	LPostTemp			[-48 -62 15]	379	
	LAngGyrus			[-37 -76 30]	498	
	LSFG			[-7 50 41]	461	
	LMFG			[-40 -2 53]	498	
	LIFGOrb			[-48 33 -4]	498	
	LIFG			[-48 16 24]	515	
MOVIE	ROI			Center Coordinate	N Voxels	
ToM	RTPJ			[48 -60 30]	376	
	LTPJ			[-48 -62 30]	368	
	PC			[0 -54 34]	382	
	DMPFC			[-6 54 36]	217	
	MMPFC			[-4 58 16]	275	
	VMPFC			[-4 56 -16]	198	
Pain	RS2			[60 -28 38]	368	
	LS2			[-62 -32 34]	269	
	Rinsula			[42 6 -6]	309	
	Linsula			[-42 -2 -4]	240	
	RMFG			[50 42 12]	142	
	LMFG			[-46 36 14]	256	
	AMCC			[0 2 42]	249	

Supplementary Table 2. Individual and Group Regions of Interest. Top half of table summarizes information about individually-defined functional regions of interest. ASL group indicates native (NS) vs. delayed (DS) signing participants. # Identified is number of participants in whom an ROI was successfully identified at p<.001, k=10 thresholds, to the Mental > Physical contrast. Peak coordinates are in MNI space. Bottom half of table provides information about group ROIs.

Supplementary Table 3

Individual ROIs		Predictor	Children	Adults
Planned	RTPJ	ASL-onset	**b=-.47, t=-2.3, p=.03**	b=-.30, t=-1.1, p=.28
		Age	b=.28, t=1.4, p=.18	
		Motion	b=.11, t=.55, p=.59	b=.28, t=1.0, p=.31
	DMPFC	ASL-onset	b=-.09, t=-.39, p=.71	b=-.17, t=-.60, p=.55
		Age	b=.34, t=1.6, p=.13	
		Motion	b=.43, t=1.6, p=.14	b=.15, t=.54, p=.59
Exploratory	LTPJ	ASL-onset	b=-.17, t=-.81, p=.43	b=-.13 t=-.46, p=.65
		Age	b=.39, t=1.9, p=.07	
		Motion	b=-.03, t=-.13, p=.90	b=.22, t=.82, p=.42
	MMPFC	ASL-onset	b=-.25, t=-1.1, p=.27	b=.05, t=.17, p=.87
		Age	b=.23, t=1.1, p=.31	
		Motion	b=.14, t=.55, p=.59	b=.01, t=.02, p=.99
	VMPFC	ASL-onset	b=-.27, t=-1.1, p=.30	b=.04, t=.13, p=.90
		Age	b=.15, t=.62, p=.55	
		Motion	b=.31, t=.84, p=.42	b=-.04, t=-.15, p=.88
	PC	ASL-onset	b=-.03, t=-.17, p=.86	b=.20, t=.71, p=.48
		Age	b=-.06, t=-3.2, p=.76	
		Motion	**b=.67, t=3.3, p=.004**	b=.02, t=.09, p=.93
**Group ROIs**		**Predictor**	**Children**	**Adults**
Planned	RTPJ	ASL-onset	**b=-.52, t=-2.7, p=.01**	b=-.24, t=-.87, p=.39
		Age	b=.28, t=1.4, p=.17	
		Motion	b=.0001, t=-.001, p=.99	b=.32, t=1.2, p=.25
	DMPFC	ASL-onset	b=-.29, t=-1.3, p=.21	b=-.02, t=-.08, p=.94
		Age	b=.17, t=.78, p=.45	
		Motion	b=.03, t=.14, p=.89	b=-.09, t=-.32, p=.75
Exploratory	LTPJ	ASL-onset	b=-.22, t=-1.0, p=.31	b=-.37 t=-1.4, p=.18
		Age	b=.41, t=2.02, p=.06	
		Motion	b=-.11, t=-.56, p=.58	b=.33, t=1.2, p=.23
	MMPFC	ASL-onset	b=-.34, t=-1.6, p=.13	b=-.10, t=-.35, p=.73
		Age	b=.25, t=1.2, p=.25	
		Motion	b=-.20, t=-.93, p=.36	b=.07, t=.24, p=.82
	VMPFC	ASL-onset	b=.33, t=1.6, p=.12	b=.34, t=1.3, p=.20
		Age	b=-.24, t=-1.2, p=.25	
		Motion	b=.38, t=1.9, p=.07	b=-.04, t=-.18, p=.86
	PC	ASL-onset	b=-.29, t=-1.3, p=.20	b=-.12, t=-.45, p=.65
		Age	b=.24, t=1.1, p=.27	
		Motion	b=-.34, t=-.16, p=.88	b=.25, t=.92, p=.36

Supplementary Table 3. ASL Story Task: Response Selectivity by ToM ROI. Statistical results of linear regressions testing for significant effects of age of ASL onset (ASL-onset), age, and motion on response selectivity, by ROI. Planned tests in the main analyses focused on responses in RTPJ and DMPFC; analyses of responses in other ToM ROIs (LTPJ, MMPFC, VMPFC, PC) were exploratory. Significant effects are in bold: selectivity of RTPJ is reduced based on age of ASL onset, among children. These results do not reflect adjustments for multiple comparisons (planned analyses reported in the main text test the primary regions of interest in a single test). All results from individual ROI analyses were confirmed in group ROIs. Source data are provided (https://osf.io/kyu3f/).

Supplementary Table 4

**Response Lateralization of ToM Response**	**Predictor**	**Children**	**Adults**	**Full Sample**
lm(Laterality ~ ASL-onset + Age + Motion)	ASL-onset	b=.15, t=.68, p=.51	b=.01, t=.02, p=.98	b=.15, t=.85, p=.40
	Age	b=-.01, t=-.04, p=.97	NA	b=.41, t=1.5, p=.15
	Motion	b=-.14, t=-.63, p=.54	b=.16, t=.60, p=.56	b=-.05, t=-.27, p=.79
**Response Lateralization of Language Response**	**Predictor**	**Children**	**Adults**	**Full Sample**
lm(Laterality ~ ASL-onset + Age + Motion)	ASL-onset	b=-.14, t=-.67, p=.51	b=.11, t=.40, p=.70	b=-.05, t=-.27, p=.79
	Age	b=.13, t=.63, p=.54	NA	b=-.20, t=-.71, p=.48
	Motion	b=.23, t=1.1, p=.29	b=.13, t=.47, p=.64	b=.24, t=1.4, p=.18
**Inter-Region Correlations between ToM and Language Regions**	**Predictor**	**Children**	**Adults**	**Full Sample**
lm(wi-ToM ~ ASL-onset + Age + Motion)	ASL-onset	b=-.06, t=-.30, p=.77	b=.40, t=1.6, p=.12	
	Age	b=.15, t=.72, p=.48	NA	
	Motion	b=-.33, t=-1.5, p=.14	**b=-.67, t=-2.7, p=.01**	
lm(wi-Lang ~ ASL-onset + Age + Motion)	ASL-onset	b=-.01, t=-.04, p=.97	b=-.17, t=-.65, p=.52	
	Age	b=.05, t=.23, p=.82	NA	
	Motion	b=-.32, t=-1.5, p=.15	b=-.26, t=-1.0, p=.32	
lm(ac-ToM-Lang ~ ASL-onset + Age + Motion)	ASL-onset	b=.09, t=.42, p=.68	b=-.46, t=-1.9, p=.06	
	Age	b=.17, t=.79, p=.44	NA	
	Motion	b=-.23, t=-1.1, p=.29	b=-.09, t=-.37, p=.71	

Supplementary Table 4. ASL Story Task: Predictors of Response Lateralization & Inter-Region Correlations. Left column indicates neural measure of interest (grey headers) and linear regression equations used for statistical results. The beta values, t-values, and p-values of each tested predictor are given per linear regression, tested in children (n=8 delayed signers; n=16 native signers), adults (n=16 delayed signers, n=20 native signers), and, when possible given identical paradigms, the full sample. Note that the full sample results are not independent from the child-only and adult-only results. ASL-onset is a continuous variable for the age of first exposure to ASL (0 for native signers). Age is a continuous variable in regressions within the child sample, and a factor (child vs. adult) within the full sample. Motion refers to mean translation. Significant predictors are indicated in bold; no adjustments for multiple comparisons were made. Source data are provided (https://osf.io/kyu3f/).

Supplementary Table 5

**Movie Task**				
**Functional Maturity of ToM Response**	**Predictor**	**Children**	**Adults**	**Full Sample**
lm(FM ~ ASL-onset + Age + ASL-onset*Age + Motion)	ASL-onset	b=.42, t=1.5, p=.14	b=-.16, t=-.56, p=.58	b=.003, t=.02, p=.99
	Age	b=.25, t=1.3, p=.21	NA	b=-.40, t=-1.5, p=.14
	Motion	**b=-.31, t=-2.1, p=.048**	b=-.09, t=-.31, p=.76	b=-.25, t=-1.5, p=.15
	ASL-onset*Age	**b=-.73, t=-2.1, p=.04**	NA	NA
lm(FM ~ ASL-onset + Age + Motion)	ASL-onset	b=-.08, t=-.48, p=.64	b=-.16, t=-.56, p=.58	b=.003, t=.02, p=.99
	Age	b=.49, t=2.96, p=.007	NA	b=-.40, t=-1.5, p=.14
	Motion	**b=-.39, t=-2.5, p=.02**	b=-.09, t=-.31, p=.76	b=-.25, t=-1.5, p=.15
**Response Magnitude in ToM Network to ToM Events**	**Predictor**	**Children**	**Adults**	**Full Sample**
lme(RM ~ ASL-onset + Age + Event + Motion)	ASL-onset	b=-.05, t=-.53, p=.60	b=-.15, t=-.70, p=.49	b=-.03, t=-.23, p=.82
	Age	**b=.23, t=2.2, p=.03**	NA	b=-.36, t=-1.9, p=.06
	Event (T01)	b=-.002, t=-.01, p=.99	b=-.38, t=-1.8, p=.08	b=-.22, t=-1.4, p=.16
	Event (T04)	**b=-.79, t=-3.4, p=.001**	b=-.22, t=-1.0, p=.31	**b=-.43, t=-2.7, p=.008**
	Motion	**b=-.30, t=-3.1, p=.005**	b=-.04, t=-.21, p=.83	b=-.17, t=-1.4, p=.17
**Analyses in Individually-Defined RTPJ**				
**Functional Maturity of RTPJ Response**	**Predictor**	**Children**	**Adults**	**Full Sample**
lm(FM-RTPJ ~ ASL-onset + Age + ASL-onset*Age + Motion)	ASL-onset	b=.08, t=.34, p=.74	b=-.34, t=-1.2, p=.23	b=-.17, t=-.93, p=.36
	Age	b=.10, t=.35, p=.73	NA	b=.05, t=.16, p=.88
	Motion	b=-.29, t=-1.2, p=.25	b=.04, t=.14, p=.89	b=-.09, t=-.50, p=.62
**Response Magnitude in RTPJ to ToM Events**	**Predictor**	**Children**	**Adults**	**Full Sample**
lme(RM-RTPJ ~ ASL-onset + Age + Event + Motion)	ASL-onset	b=-.18, t=-1.6, p=.13	b=-.08, t=-.42, p=.68	b=-.13, t=-1.1, p=.29
	Age	b=.23, t=1.6, p=.12	NA	b=.05, t=.26, p=.79
	Event (T01)	b=.01, t=.03, p=.98	b=-.18, t=-.74, p=.46	b=-.10, t=-.58, p=.56
	Event (T04)	**b=-1.0, t=-4.1, p=.0002**	**b=-.65, t=-2.7, p=.009**	**b=-.83, t=-4.7, p=7.8x10^{-6}**
	Motion	b=.11, t=.99, p=.34	b=.01, t=.06, p=.95	b=.07, t=.62, p=.54

Supplementary Table 5. Movie-Viewing Task: Predictors of functional maturity and response magnitude. Left column indicates neural measure of interest (grey headers) and regression equations. Regressions summarized in the top half of the table reflect measures of the ToM network; regressions summarized in the bottom half of the table reflect measures extracted from individual RTPJ ROIs used for ASL Story task analyses. The standardized beta values, t-values, and p-values of each tested predictor are given per regression, tested in children (n=9 delayed signers, n=19 native signers), adults (n=11 delayed signers, n=18 native signers), and the full sample. Note that the full sample results are not independent from the child-only and adult-only results. ASL-onset is a continuous variable for the age of first exposure to ASL (0 for native signers). Age is a continuous variable in regressions within the child sample, and a factor (child vs. adult) within the full sample; negative effects of age group in the full sample indicate smaller values among children relative to adults. Event levels include T01, T02, and T04. Motion refers to mean translation. Significant predictors are indicated in bold; no adjustments for multiple comparisons were made. Source data are provided (https://osf.io/kyu3f/).
