# Supplementary material for: Reduced neural selectivity for mental states in deaf children with delayed exposure to sign language
Source: Nat Commun. 2020 Jun 26;11:3246. doi: 10.1038/s41467-020-17004-y (PMC7319957; doi:10.1038/s41467-020-17004-y)
Supplement: Supplementary file 3 — Reporting Summary [file 41467_2020_17004_MOESM3_ESM.pdf]

natureresearch

Corresponding author(s): Hilary Richardson
Last updated by author(s): May 27, 2020

Reporting Summary

Nature Research wishes to improve the reproducibility of the work that we publish. This form provides structure for consistency and transparency in reporting. For further information on Nature Research policies, see Authors & Referees and the Editorial Policy Checklist.

Statistics

For all statistical analyses, confirm that the following items are present in the figure legend, table legend, main text, or Methods section.

n/a	Confirmed	
☐	☒	The exact sample size (*n*) for each experimental group/condition, given as a discrete number and unit of measurement
☐	☒	A statement on whether measurements were taken from distinct samples or whether the same sample was measured repeatedly
☐	☒	The statistical test(s) used AND whether they are one- or two-sided<br>*Only common tests should be described solely by name; describe more complex techniques in the Methods section.*
☐	☒	A description of all covariates tested
☐	☒	A description of any assumptions or corrections, such as tests of normality and adjustment for multiple comparisons
☐	☒	A full description of the statistical parameters including central tendency (e.g. means) or other basic estimates (e.g. regression coefficient) AND variation (e.g. standard deviation) or associated estimates of uncertainty (e.g. confidence intervals)
☐	☒	For null hypothesis testing, the test statistic (e.g. *F*, *t*, *r*) with confidence intervals, effect sizes, degrees of freedom and *P* value noted<br>*Give P values as exact values whenever suitable.*
☒	☐	For Bayesian analysis, information on the choice of priors and Markov chain Monte Carlo settings
☒	☐	For hierarchical and complex designs, identification of the appropriate level for tests and full reporting of outcomes
☐	☒	Estimates of effect sizes (e.g. Cohen's *d*, Pearson's *r*), indicating how they were calculated

Our web collection on statistics for biologists contains articles on many of the points above.

Software and code

Policy information about availability of computer code

Data collection	Behavioral data during the primary fMRI experiment were collected via custom scripts in Matlab 2010a.
Data analysis	FMRI data were analyzed using SPM8 (version R4010; http://www.fil.ion.ucl.ac.uk/spm) and custom software written in Matlab 2017a (MathWorks, Natick, MA). Corrected random effects analyses were completed with SPM5's SnPM5b toolbox (SPM5 version 1111). Identification of motion artifact in the fMRI data was completed using the Artifact Detection Toolbox (https://www.nitrc.org/projects/artifact_detect). Statistical analyses were conducted in Matlab 2017a and R 3.3.3 (https://www.r-project.org/). Custom code for statistical analyses is provided on the Open Science Framework (https://osf.io/kyu3f/).

For manuscripts utilizing custom algorithms or software that are central to the research but not yet described in published literature, software must be made available to editors/reviewers. We strongly encourage code deposition in a community repository (e.g. GitHub). See the Nature Research guidelines for submitting code & software for further information.

Data

Policy information about availability of data

All manuscripts must include a data availability statement. This statement should provide the following information, where applicable:

- Accession codes, unique identifiers, or web links for publicly available datasets
- A list of figures that have associated raw data
- A description of any restrictions on data availability

Because these data were collected up to eight years ago, and prior to the normalization of data sharing, the conditions of our ethics approval did not include public archiving of individual raw MRI or behavioral data. Processed data, which enables reproducing all statistical results and figures, fMRI story stimuli, and behavioral ToM tasks are publicly available on OSF (https://osf.io/kyu3f/). To ensure anonymity of participants, participant ages and ages of ASL onset have been z-scored. The source data underlying Figures 1, 2, 3 and Supplementary Figures 2, 3, 4a, 5, 6, 7, 9, and 10 are provided as a Source Data file. A reporting summary for this Article is available as a Supplementary Information file. The corresponding author welcomes any additional requests for materials.

Field-specific reporting

Please select the one below that is the best fit for your research. If you are not sure, read the appropriate sections before making your selection.

☒ Life sciences ☐ Behavioural & social sciences ☐ Ecological, evolutionary & environmental sciences

For a reference copy of the document with all sections, see nature.com/documents/nr-reporting-summary-flat.pdf

Life sciences study design

All studies must disclose on these points even when the disclosure is negative.

Sample size

We measured ToM behaviorally and neurally in 33 fluent signing children (n=21 native signers (NS), n=12 delayed signers (DS); 4-12 years old) and adults (n=36). Native signing children included deaf children (n=15) and hearing children (n=6) born to d/Deaf parents. The age of first exposure to ASL, a proxy for linguistic experience, ranged among DS children from .25 – 7 years. Unlike many prior behavioral studies of this population, all delayed signing participants were exposed to a full sign language, not a homesign or to English alone (e.g., oral children), and had fluent, age-appropriate comprehension of ASL morphology and syntax at the time of the study (see Supplementary Table 1).

The narrow linguistic criteria of inclusion, along with the typical constraints of functional magnetic resonance imaging (fMRI) studies – that participants have no known neurological or cognitive disabilities, or non-removable metal (e.g., cochlear implants) – required effortful recruitment of a scarce population over four years, and ultimately resulted in a unique dataset. We did not employ statistical methods to determine a sufficient sample size; instead, we endeavored to recruit as many eligible participants as possible given the funding that we had to complete the study. Participants were recruited via the researchers' social networks, by snowball sampling, and, in the case of child participants, with help from several schools for the deaf. Any potential participant who fit the criteria outlined above was recruited and offered compensation for travel costs (several children were flown in from other states in order to participate).

Some participants did not complete all fMRI tasks, or were excluded from fMRI analyses due to excessive motion, leaving n=8 delayed signing and n=16 native signing children in analyses of the story fMRI task, and n=9 delayed signing and n=19 native signing children in analyses of the movie fMRI task. Due to the relatively small number of child participants, we include data from a large sample of age-matched neurotypical hearing children in figures, so that the data from the current sample can be visualized within a larger distribution of participants. These participants are described within the legends of the figures that include them; statistical tests were conducted on the current sample of delayed and native signers only.

In sum, our sample size reflects the maximum number of eligible children we could identify and recruit using exhaustive recruitment approaches over the course of four years. However, because our sample size is small (especially for children who experienced delayed exposure to ASL), we (1) visualize the data within a larger distribution of hearing children when possible, (2) explicitly note that null results should be treated with caution, and (3) discuss the importance of replication attempts with larger sample sizes (which will likely require using alternative methods that facilitate recruiting a larger sample size; e.g., functional near-infrared spectroscopy).

Data exclusions

Participants were excluded from analyses of the primary (story) fMRI task if they completed fewer than two runs of the task (n=6 children), or if they moved excessively during the scan (n=3 children for the primary story task; n=3 children for the movie task). Thresholds for exclusions based on participant motion were set based on prior studies and pre-registered (https://osf.io/mhgp8).

Replication

A replication study would be challenging, due to the scarcity of the population of interest (fluent ASL-signing children who are otherwise neurotypical, and metal-free (i.e., no cochlear implants), and including participants who are non-native signers and vary in amount of early language delay).

However, due to the relatively small number of child participants in this study, we include data from age-matched neurotypical hearing children in figures, such that the data from the current sample (which shows relative delays in both behavioral and neural measures of ToM in children with delayed access to language) can be visualized within a larger distribution of participants.

Randomization

Experimental groups were delayed and native signers. Native signers were exposed to American Sign Language from birth; delayed signers were exposed to ASL after a delay. We additionally collected age, current receptive ASL score (measured via the ASL-RST), non-verbal IQ score (KBIT-II), and performance on an executive functions task in order to control for and understand the role of these covariates in addition to the covariate of interest (age of ASL onset).

Blinding

Detailed information about timing of exposure to and proficiency in ASL was collected during recruitment for screening purposes, and experimenters who conducted data collection were not naive to the participant's language history. Recruitment/screening and data collection were carried out by a relatively small team of individuals, who were proficient or native ASL signers, and therefore would likely be able to tell the difference between signing of native and non-native signers (similar to how spoken accents are detectable). Additionally, child participants were accompanied by parents to the testing session, which would often make their group membership/language history obvious to experimenters (delayed signers were children of parents who did not sign at the time of their birth). However, our primary measures of interest (neural responses during fMRI experiments) were not susceptible to biases in experimenter administration, because the fMRI experiments involved watching movies that were developed prior to any data collection.

Reporting for specific materials, systems and methods

We require information from authors about some types of materials, experimental systems and methods used in many studies. Here, indicate whether each material, system or method listed is relevant to your study. If you are not sure if a list item applies to your research, read the appropriate section before selecting a response.

Materials & experimental systems

n/a	Involved in the study
☒	☐ Antibodies
☒	☐ Eukaryotic cell lines
☒	☐ Palaeontology
☒	☐ Animals and other organisms
☐	☒ Human research participants
☒	☐ Clinical data

Methods

n/a	Involved in the study
☒	☐ ChIP-seq
☒	☐ Flow cytometry
☐	☒ MRI-based neuroimaging

Human research participants

Policy information about studies involving human research participants

Population characteristics

Child participants were 21 native signers (4-12.7 years old, M(SD) = 8.19(2.2) years, 10 female), who received exposure to ASL from birth from deaf parents (15 deaf children and 6 hearing children), and 12 delayed signers (6.2-12.1 years old, M(SD) = 9.29(1.9) years, 5 female), who were born deaf to hearing parents and received exposure to ASL after an initial delay of .25 – 7 years (M(SD) = 2.9 (2.2) years).

Among adults, the native signing participants (n=20, 20-53 years old, M(SD) = 30.1(9.4)) included deaf people who had deaf parents (n=10), hearing people who had deaf parents (n=7), and deaf people who had hearing parents and deaf older siblings (n=3). All delayed signing adults were born deaf to hearing parents (n=16, 21-64 years old, M(SD) = 37.9(12.8) years, 4 female, mean delay before ASL exposure M(SD) = 6.5 (6.2) years). Delayed signing adults included 12 "early signers" (1.5-7 year delay, M(SD) = 3.3 (1.9) years) and 4 "late signers" (exposure to ASL at ages 11, 15, 18, and 20 years). All adult participants contributed fMRI story task data (n=16 DS, n=20 NS); n=11 delayed signing and n=18 contributed fMRI movie task data. See Supplementary Table 1 for additional information about participants.

Recruitment

Participants were recruited via the researchers' social networks, by snowball sampling, and, in the case of child participants, with help from several schools for the deaf over the course of four years. All participants were screened by a native ASL signer; only fluent signers were recruited to participate. The narrow linguistic criteria of inclusion, along with the typical constraints of neuroimaging studies – that participants have no known neurological or cognitive disabilities, or non-removable metal (e.g., cochlear implants) – resulted in effortful recruitment and testing of a unique and scarce population over the course of four years. Any potential participant who fit the criteria outlined above was recruited and offered compensation for travel costs. Thus, we attempted to be exhaustive in our recruitment approach and covered travel costs for participants and their families to come to the lab to participate. Of course, like any scientific study, our sample is comprised of individuals who were willing and able to participate and to contribute usable data.

We note in our Discussion section that in contrast to our results (which finds an effect of age of ASL onset on ToM behavior and RTPJ selectivity), prior studies find broad cognitive effects of delayed access to language (e.g., delayed development of language comprehension and production, literacy, executive functions, differences in language-related brain development, and consequences for mental health). We suspect that this discrepancy is related to characteristics of our sample: all of our child participants, and almost all of our adult participants, experienced a relatively short delay prior to learning ASL, and all were proficient signers at the time of the study. Broader cognitive and emotional effects of language delay may be strongest following longer delays that more substantially impede ASL acquisition.

Additionally, of all children born deaf to non-signing parents, there are many factors that drive parents' choice to have their children receive cochlear implants and oral education, versus be taught American Sign Language as their first language. Given this complex background, we cannot be sure that the two populations are matched along every developmentally-relevant dimension.

Ethics oversight

All assent and consent forms and experimental protocols were approved by the Committee on the Use of Humans as Experimental Subjects at Massachusetts Institute of Technology.

Note that full information on the approval of the study protocol must also be provided in the manuscript.

Magnetic resonance imaging

Experimental design

Design type

The primary fMRI (story) task used a block design. The movie fMRI experiment was a naturalistic viewing experiment viewed during a single 5.6-minute run.

Design specifications

During the primary fMRI (story) task, child participants viewed 24 stories (8 per condition (Mental, Social, Physical)) across four 8.3-minute runs. Adult participants viewed 30 stories (10 per condition) across five 10.3-minute runs. All children saw the same 8 stories per condition; each adult participant saw 10 of 14 stories per condition. Participants also saw 8 (child) or 20 (adult) clips of non-signs; the non-sign stimuli were used in control analyses of language processing.

Each run included six 60-second blocks (2 per condition), as well as 10 seconds of rest at the beginning and end of each run. The order of conditions in each run was palindromic (e.g., A B C C B A) and counterbalanced across runs.

Behavioral performance measures

To encourage engagement during the story task, stories were presented in two consecutive segments: the main story (29-41s) and a final sentence containing the story ending or the ending of an unrelated story (4-8s). Only the main story, and not the ending, was included in the fMRI data analysis. Half of the presented stories/non-signs were followed by the correct ending ("Yes" response). Incorrect endings were drawn randomly from another story. After each non-sign stimulus, participants saw an identical or novel sequence of non-signs and had to judge whether the final ending sequence matched the signs in the initial sequence. After the stimuli completed there was a 3s pause during which participants responded to indicate whether the ending fit the main story by pushing one of two buttons ("Yes" or "No").

Behavioral performance on the ASL story task was measured via accuracy (mean and standard deviation of the proportion of questions answered correctly) on trials from included functional runs only; trials from runs that were excluded due to excessive motion were not analyzed. Additionally, accuracy during the linguistic conditions (Mental, Social, and Physical) were used to test for behavioral performance differences by age of ASL onset and age.

Acquisition

Imaging type(s)

Structural and functional

Field strength

3T

Sequence & imaging parameters

Whole-brain structural and functional MRI data were acquired on a 3-Tesla Siemens Tim Trio scanner located at the Athinoula A. Martinos Imaging Center at MIT, using custom 32-channel phased-array head coils made for children112 or the standard Siemens 32-channel head coil. T1-weighted structural images were collected in 176 interleaved sagittal slices with 1mm isotropic voxels (GRAPPA parallel imaging, acceleration factor of 3; adult coil: FOV: 256mm; pediatric coils: FOV: 192mm). Functional data were collected with a gradient-echo EPI sequence sensitive to Blood Oxygen Level Dependent (BOLD) contrast in 3 mm isotropic voxels with a 20% slice gap (n=7 adults, n=28 children) or 3.13 mm isotropic voxels with no slice gap (n=29 adults, n=1 child) in 32 interleaved near-axial slices aligned with the anterior/posterior commissure, and covering the whole brain (EPI factor: 64; TR: 2s, TE: 30ms, flip angle: 90°); all functional data were subsequently upsampled in normalized space to 2mm isotropic voxels. Prospective acquisition correction was used to adjust the positions of the gradients based on the participant's head motion one TR back. 310 (adults) or 250 (children) volumes were acquired in each run of the story task. 155 volumes were acquired during the single run of the movie-viewing task. Four dummy scans were collected to allow for steady-state magnetization in each run.

Area of acquisition

Whole-brain structural and functional MRI data were acquired.

Diffusion MRI ☐ Used ☒ Not used

Preprocessing

Preprocessing software

FMRI data were analyzed using SPM8 (http://www.fil.ion.ucl.ac.uk/spm) and custom software written in Matlab (MathWorks, Natick, MA). Functional images were registered to the first image of each run; that image was registered to each participant's anatomical scan, and each participant's anatomical scan was normalized (with linear and non-linear transformations) to a common brain space (Montreal Neurological Institute (MNI) template). All data were smoothed using a Gaussian filter (5mm kernel).

Normalization

Functional images were registered to the first image of each run; that image was registered to each participant's anatomical scan, and each participant's anatomical scan was normalized (with linear and non-linear transformations) to a common brain space (Montreal Neurological Institute (MNI) template).

Normalization template

Montreal Neurological Institute (MNI) template.

Noise and artifact removal

Motion artifact timepoints were identified using the ART toolbox (https://www.nitrc.org/projects/artifact_detect/) as timepoints when there was 1) more than 2mm of motion or 2) a fluctuation in global signal >3 SD. Runs were excluded if one-third or more of the timepoints were identified as motion artifacts. Participants were excluded from analyses of the story task if they had fewer than two runs of usable data (n=3 children). The movie task consisted of one run; 3 children were excluded for excessive motion during this task.

We used a general-linear model to analyze BOLD activity of each participant as a function of condition. Data were modeled in SPM8 using a standard hemodynamic response function (HRF). Boxcar regressors for each condition and the response period were convolved with the standard HRF, and nuisance covariates were included for run effects, motion artifact timepoints, and signal of no interest (five PCA-based regressors generated with CompCor (Behzadi et al., 2007) from individually tailored white matter masks, eroded by two voxels in each direction).

All analysis decisions (including preprocessing and motion exclusion and treatment) and planned analyses for the story task were pre-registered via OSF (ToM: https://osf.io/mhgp8; Language: https://osf.io/7y263). Story and movie task analyses were constrained by methods used in prior studies, in order to facilitate comparisons across studies (https://osf.io/jh68b/; Richardson et al., 2018).

Volume censoring

Motion artifact timepoints were identified using the ART toolbox (https://www.nitrc.org/projects/artifact_detect/) as timepoints when there was 1) more than 2mm of motion or 2) a fluctuation in global signal >3 SD. Runs were excluded if one-third or more of the timepoints were identified as motion artifacts. Participants were excluded from analyses of the story task if they had fewer than two runs of usable data (n=3 children). The movie task consisted of one run; 3 children were excluded for excessive motion during this task.

Amount of motion per group is visualized in Supplementary Figure 5. We additionally provide statistical evidence that amount of motion among children was uncorrelated with age, theory of mind behavior, and age of ASL onset (Supplementary Note 3).

Statistical modeling & inference

Model type and settings

Primary (Story) fMRI Task:
We used a general-linear model to analyze BOLD activity of each participant as a function of condition. Data were modeled in SPM8 using a standard hemodynamic response function (HRF). Boxcar regressors for each condition and the response period were convolved with the standard HRF, and nuisance covariates were included for run effects, motion artifact timepoints, and signal of no interest (five PCA-based regressors generated with aCompCor (Behzadi et al., 2007) from individually tailored white matter masks, eroded by two voxels in each direction).

We additionally conducted whole-brain random effects analyses on the Mental > Physical and Physical > Non-Sign contrasts in order to visualize regions active for ToM and language processing, respectively, and to test for differences in activation by age of ASL onset.

Effect(s) tested

Primary (Story) fMRI Task:
For ToM regions of interest, we extracted the mean beta value per condition per region, and calculated selectivity as (Mental – Social) / (Mental – Physical)*100. This calculation has been used in previous studies of ToM brain region development (Gweon et al., 2012; https://osf.io/jh68b/). Because the Mental and Physical difference is used to define ROIs, the selectivity measure focuses on the relative difference between Mental and Social conditions. Based on previous analyses, we expected the selectivity measure to be between -50 and 200 in individual ROIs, and excluded values outside of this range (https://osf.io/mhgp8; n=1 NS adult DMPFC value was excluded, selectivity = -64.2).

To measure responses in language regions of interest, we calculated the mean beta value to Physical – Non-Sign conditions, multiplied by 100. We also conducted a parallel analysis in ROIs spatially tailored to each individual, by extracting responses from the 50 voxels with the highest T-values to the Physical > Non-Sign contrast within these eleven regions.

We additionally measured the lateralization of ToM and language neural responses, and conducted inter-region correlation (IRC) analyses on the responses within and across group ToM and language brain regions. We measured the lateralization of the neural response to the Mental > Physical (ToM) contrast in ToM regions, and to the Physical > Non-Sign (Language) contrast in language regions. For the ToM lateralization analysis, we created a large ROI encompassing the bilateral temporal lobe from publicly available right hemisphere search spaces (http://saxelab.mit.edu/ToMgroupMaps.php) (Dufour et al., 2013); the right hemisphere was flipped to create the left hemisphere ROI. For the language lateralization analysis, we created a composite language ROI in the left hemisphere using the nine left hemisphere ROIs described above, and the mirror image of these regions in the right hemisphere. The lateralization index (LI) was calculated as the number of suprathreshold voxels in the left hemisphere minus the number of suprathreshold voxels in the right hemisphere, divided by the sum of the number of suprathreshold voxels in the left and right hemispheres ((NumVoxL - NumVoxR)/(NumVoxL + NumVoxR)) (Desmond et al., 1995). We used a threshold of $p<.001$, uncorrected, and confirmed that results were not threshold dependent by repeating analyses at $p<.01$. We planned to exclude participants if the denominator was smaller than 20, indicating fewer than 20 suprathreshold voxels, bilaterally; zero participants were excluded based on this criterion. Large positive LI values indicate strong left lateralization, whereas an LI of zero indicates no response lateralization. See Supplementary Notes 8 and 9.

Movie fMRI Task:
Analyses of the movie viewing task followed methods developed in a prior study (Richardson et al., 2018). We tested whether the functional maturity of each participant's response timecourse (i.e. similarity to adults) varied as a function of the age at which they were first exposed to ASL. We calculated the Pearson correlation between each participant's ToM timecourse (averaged across ToM group ROIs, TRs 11:155) and an average adult timecourse derived from the prior study.

We additionally tested for differences in response magnitude in ToM regions to three events in the movie. In the previous study, response magnitude to two of these events increased with age in three to twelve year old children (events T01 and T02). In addition, response magnitude to the third event (event T04) was positively correlated with performance on a linguistic ToM behavioral battery, controlling for age and motion (and correcting for multiple comparisons using a Bonferroni correction).

Finally, we conducted Inter-Region Correlation (IRC) analyses on response timecourses from ToM brain regions and the extended "Pain Matrix" (Supplementary Note 10).

Statistics:
We used linear regressions to test if each of these neural response properties in ToM or language brain regions differed as a function of age of first exposure to ASL, a continuous variable ranging between .25-7 years in delayed signing children, and 1.5-20 years in delayed signing adults. Age of ASL onset for native signers was zero. Statistical analyses were conducted in Matlab 2017a (MathWorks, Natick, MA) and R 3.3.3 (https://www.r-project.org/). We conducted regressions within children, within adults, and, when possible, across the full sample. We included age group (regressions across the full sample; child vs. adult) or age (regressions within children only; continuous variable) as a covariate. All planned regressions on selectivity during the story task included data from both ToM ROIs (RTPJ, DMPFC), and tested for a significant effect (and interaction) of ROI; regressions on mean contrast value in language ROIs similarly included ROI as a covariate. Regressions on the response magnitude to the three ToM events during the movie task included data from three events (T01, T02, T04), and tested for a significant effect (and interaction) of event. As specified in the analysis plan, we first tested for significant age (or age-group)*ASL-onset interactions, and if the interaction term was not significant, removed it from the regression. Regressions included motion (mean translation) as

a between-subject predictor in all regressions, and a subject identifier as a random effect in those that included non-independent measurements (e.g., data from two ROIs, or three ToM events, per subject). Continuous regression variables were standardized, such that the units of the regression beta coefficients are the same.

Specify type of analysis: ☐ Whole brain ☐ ROI-based ☒ Both

Anatomical location(s)

Individual "theory of mind" ROIs were defined as contiguous (minimum k=10) suprathreshold (p<.001) voxels within a 9mm radius sphere of the peak voxel to the Mental > Physical contrast, within previously defined region search spaces. Region search spaces were defined in a random effects analysis of a False-Belief > False-Photograph contrast in a independent group of 462 neurotypical adults (Dufour et al., 2013). These search spaces are publicly available for download (http://saxelab.mit.edu/use-our-theory-mind-group-maps).

To test whether language delay affected responses in cortical language regions, we also defined group language ROIs as 10mm spheres drawn around peak coordinates reported by Fedorenko et al., 2010: including left inferior frontal gyrus, orbital inferior frontal gyrus, medial frontal gyrus, superior frontal gyrus, anterior temporal lobe, middle anterior temporal lobe, middle posterior temporal lobe, posterior temporal lobe, angular gyrus, and right middle anterior temporal lobe and middle posterior temporal lobe; cerebellar ROIs were excluded due to lack of coverage.

Statistic type for inference
(See Eklund et al. 2016)

Combined voxel and cluster-wise correction (theta value of .5) was implemented for all whole-brain analyses.

Correction

Whole-brain analyses were corrected for multiple comparisons by estimating the false-positive rate via 5,000 Monte Carlo permutations using the SnPM5b toolbox for SPM5 (version 1111; http://www.fil.ion.ucl.ac.uk/spm/software/spm5/), at p<.05. For whole-brain analyses of delayed signing children only (Story Task: n=8; Movie Task: n=9), the maximum number of Monte Carlo permutations (given the sample size) were used (256 permutations; 512 permutations, respectively).

Models & analysis

n/a	Involved in the study
☐	☒ Functional and/or effective connectivity
☒	☐ Graph analysis
☒	☐ Multivariate modeling or predictive analysis

Functional and/or effective connectivity

We conducted inter-region correlation (IRC) analyses on the responses within and across group ToM and language brain regions (see Supplementary Table 2 for information about ROIs), using the procedure of a prior study (Richardson, Lisandrelli, Riobueno-Naylor, & Saxe, 2018). Preprocessed, scaled timecourses were extracted from each voxel per ROI. The five PCA-based noise regressors and motion artifact timepoint regressors (included as nuisance regressors in the story task) were regressed from these timecourses, and the residual timecourses were high-pass filtered with a cut-off of 100 seconds. Timecourses from voxels within an ROI were averaged, creating one timecourse per ROI, and artifact timepoints were subsequently NaNed. Each ROI timecourse was correlated with every other ROI timecourse, per subject, and these correlation values were Fisher z-transformed. Within-ToM and within-Lang network correlations were calculated as the average correlation value between brain regions within each network. Similarly, across-ToM-Lang correlations were calculated as the average correlation value between ToM and Language brain regions. In order to test if different brain networks (ToM-Lang) were functionally dissociated, we used t-tests to compare within- versus across-network correlations.

Additionally, because a prior study found that responses in ToM and Pain networks are driven by “Partly Cloudy” (Reher & Sohn, 2009), and that regions within these two networks become increasingly correlated within-network, and increasingly anti-correlated across-network, during childhood (Richardson et al., 2018), we conducted IRC analyses on the same ToM and pain group regions of interest utilized in the prior study, using the same procedures as those described above (which additionally match the methods used in the prior study; Supplementary Note 10).
