# Supplementary material for: Reduced neural selectivity for mental states in deaf children with delayed exposure to sign language
Source: Nat Commun. 2020 Jun 26;11:3246. doi: 10.1038/s41467-020-17004-y (PMC7319957; doi:10.1038/s41467-020-17004-y)
Supplement: Supplementary file 2 — Peer Review File [file 41467_2020_17004_MOESM2_ESM.pdf]

Reviewers' comments:

Reviewer #1 (Remarks to the Author):

Reduced Neural Selectivity for Mental States in Deaf Children with Delayed Exposure to Sign Language

The manuscript reports data collected from deaf children who were either native signers or who had been exposed initially to sign only after varying amounts of time. The goal of the project is to determine whether delay of exposure (up to 7 years) to linguistic input influences theory of mind processing, both behaviorally and neurally. The focus is on determining the "mechanism of correlation" between language skills and theory of mind ability. Overall, behavioral data show delays on theory of mind tests given delays in initial exposure to language. Neurally, the right temporoparietal junction showed reduced response selectivity to theory of mind stimuli/tasks in the language delay sample of signers relative to the native signers. The authors argue that the data "…provide compelling evidence that linguistic experience facilitates developmental change in ToM specific representations, via the development of a selective brain region for ToM."

The authors are to be commended for pursuing this difficult study. As they note, the data are valuable. The results are interesting, if not surprising: language experience introduces complex concepts, which in turn develop understanding of mental states in others. This experiment highlights an ideal way to establish this effect. What is unclear is what the debate is actually about. As quoted above, the authors argue that their findings provide clear evidence of language influencing development of the neural underpinnings that support theory of mind abilities. The only alternative would be that theory of mind is somehow magically (innately) intact at birth. Does anyone make this argument? Moreover, the "mechanism of correlation" that the authors purport to have determined seems uncontroversial: language experience is critical for the development of theory of mind abilities. Were there arguments to the contrary? It was not clear from the introduction, and that position seems hard to justify outside of a purely modular view of the brain. An important data point that might get at mechanism was the finding that age of ASL onset was significantly negatively correlated with standardized non-verbal IQ score in this sample (although the effect of age of ASL onset on the linguistic ToM task was significant when additionally including standardized non-verbal IQ as a covariate). This brings to mind recent findings by Matt Hall on executive function and other learning delays in those with delayed exposure to sign and/or spoken language (in the case of CI users). Although tangential to the theory of mind question being addressed here, that work might be useful to the authors in thinking about how to frame/interpret these data.

The following two aspects of the data are surprising: 1) the remarkable similarities in performance on many measures across the two groups of signers, and 2) the lack of differences in theory of mind abilities in adult native vs. delayed signers. Regarding the first point, an example of an unexpected outcomes is the following: although receptive ASL proficiency increased with age, there was no difference in receptive ASL proficiency as a function of age of ASL onset. In fact, the delayed signers averaged (nonsignificantly) better performance: M(SE) proportion correct in delayed signers: .79(.02); native signers: .70(.04). That's surprising, isn't it? Likewise, the finding that the effect doesn't hold in the two types of adult signers indicates that this processing ability is remarkably plastic in a way that the comparison abilities (e.g., face recognition, visual word form processing) are not. The authors push that comparison with their fMRI findings, but the similarities among the adult signers challenges that comparison.

More generally, the use of the dorsal medial prefrontal cortex as a comparison region is never justified/explained (nor is the acronym even introduced; it just appears without the full name of the region). This is one example of many aspects of the manuscript's structure that are problematic, making the logic of the authors' argument hard to follow. These are (indeed!) valuable data, but the exposition makes them harder to appreciate. It is not until discussion that the different mechanistic

arguments become clear (or clearer).

In short, the importance of the data is clear, but the presentation needs work.

Reviewer #2 (Remarks to the Author):

The current study investigates the effect of delayed exposure to sign language (ASL) on ToM performance and neural signature in children and adults born deaf. The main findings are that delayed access to ASL was associated with impaired performance and reduced functional selectivity of ToM brain regions in linguistic, but not in non-linguistic ToM tasks. The effects of delayed language exposure were only present in the children but not in the adult group. In addition, lateralization of ToM and language responses and inter-region correlations within and across the ToM and language network were analyzed, but did not show any significant effect of ASL onset. The authors conclude that these results support the view that language facilitates the functional specialization of ToM regions and is intrinsically related to ToM regions rather than merely to the expression of ToM abilities in ToM tasks.

The study addresses a very relevant question testing a scarce population of deaf children with (and without) delayed access to ASL, which is particularly well-suited to address the role of language for ToM development. Despite the small number of participants (n = 12 children with delayed ASL access), in particular, the MRI data of these children therefore constitute a very rare and valuable data set. The pattern of results, however, are relatively complex, so that I do not think that the strong conclusions of a direct causal relation of language exposure on ToM development drawn by the authors are supported. In addition, I have a number of methodological concerns and questions explained below.

1) Conclusions of a direct influence are too strong
Given the complexity of the results and the correlational nature of the findings, the authors' conclusion that early language input directly facilitates ToM development appears to be too strong.
First of all, given the correlational nature of the relation of ASL onset and ToM performance and selectivity, there could be other mediating factors, such as, social background, presence of older siblings or any other cognitive abilities that are likely to be related to both variables.
Secondly, if language exposure matters for the development of ToM concepts rather than for task comprehension and expression, ASL onset should be related to both the verbal and the minimally verbal task. The authors, however, only found a relation with the verbal task. The authors argue that ASL onset and its relation with selectivity were independent of language proficiency as measured by the ASL-RST. However, the relation could have resulted from more complex linguistic aspects of the task (not tested for in the test of ASL proficiency), as for example pragmatics, in particular given that the authors claim that the correlation might have been driven by "hard" items of the verbal ToM task, including sarcasm, lies, etc.
In addition, the authors argue that a poorer comprehension of mental aspects would have caused lower activation for the mental state condition per se rather than lower selectivity (l.435, l.456). However, in contrast to these predictions, less advanced linguistic abilities might also predict a less refined differentiation of mental versus social contents and therefore potentially less differentiated activity patterns (exactly as observed in the current study).
Finally, the authors argue that there wasn't more activity in language regions in children with later ASL onset which would have indicated that these children struggled more with the task. However, it is not possible to interpret a null-result to conclude the opposite, namely that they did not struggle more, in particular in such a small sample and so widely defined language ROIs (compared to the

much more restrictive ToM ROIs, see comment below).
The complexity of the results and ambiguity in their interpretation need to be made clearer in the abstract, conclusions, and throughout the manuscript (e.g., in the last sentence of the abstract and in the Discussion in lines 377, 433, 504), and these arguments need to be refined.

2) Relatedly, if early language deprivation impacts ToM development, how do the authors explain that there was no effect of delayed ASL onset on selectivity for ToM in adults?
3) The introduction would benefit from a better theoretical embedding of different ways how language might influence ToM development (e.g. understanding conversations in their environment might allow picking up on mental concepts, but also language might provide the framework to think about these abstract concepts as e.g. argued by de Villiers or Harris and colleagues). E.g. line 32 ff.: Not only complement structures and mental verbs were shown to relate to ToM, but also more general linguistic abilities, syntax as well as vocabulary, and there are controversial findings and theories on the direction of effects (see e.g. Milligan et al. 2007).

4) References infant ToM:
Instead of citing 3 (randomly?) selected studies of infant ToM (references 18-20), I would suggest to cite one or two review articles that give an overview of all non-verbal infant ToM studies, maybe from different perspectives including the current controversy (e.g. new meta-analysis by Barone, Corradi, & Gomila, 2019).

5) References neural basis of ToM:
Line 118 ff.: The authors write that ToM and language regions are clearly distinct. The IFG and posterior superior temporal regions as well as the structural connection between the two regions have been shown to be involved in both (e.g. Schurz et al. 2015; Grosse Wiesmann et al. 2017)
Line 119 ff.: The authors only mention the rTPJ being involved in ToM. Given that ToM tasks quite consistently activate a bilateral network of multiple brain regions (e.g., Schurz et al., 2015), I would suggest to mention all the regions of the ToM network.
Line 125 ff.: Concerning the children's literature, I would suggest to add that similar network of brain regions is involved in the development of ToM in early childhood (e.g. Richardson et al. 2017; Sabbagh et al. 2009, Grosse Wiesmann et al. 2017) in addition to an increase of selectivity in the rTPJ and other regions.

6) Specific role of the rTPJ?
Also in the results, the authors suggest a specific role of the selectivity of rTPJ rather than the DMPFC. Table 1 shows no significant effect of ROI (for the ToM ROIs) nor is there a significant interaction of ASL onset and ROI. What was the rational behind conducting post-hoc analyses in each ROI separately then?

7) Definition of the ROIs
The supplemental material suggests that the reported pattern of results also holds for ROIs defined on a group level. Given that this ROI definition is independent of individual activation in the mental state condition, I find these results more convincing and would suggest to report these in the main body of the paper (potentially instead of the functionally defined ROIs).
Relatedly, what was the rational behind defining the language ROIs in an entirely different way from the ToM ROIs and based on coordinates of one specific study? Also, I believe reference 67 is not the correct reference here? I was surprised how many regions were included in the analysis of the language ROIs, especially given how restrictive the authors were with respect to the ToM network (i.e., only including rTPJ and DMPFC rather than the entire ToM network).

8) How was ASL onset exactly defined to be included as a covariate in the regressions e.g. in Table 1? Where native and delayed signers treated separately? Or how did the authors take into account the skewed distribution of this variable?

9) Role of inhibition?
- l. 256 ff.: The authors report that participants' response inhibition was assessed, but I was unable to find the results related to this measure. How was ASL onset related to response inhibition? What role did inhibition play for the relation of ASL onset with ToM performance and with neural selectivity for mental state reasoning?

10) Item analysis
It didn't get quite clear to me whether there was a main effect of item category in the exploratory regression in l.272 ff.? This would need to be the case in order to test whether children performed worse in one category than another.

Reviewer #3 (Remarks to the Author):

This manuscript aims to investigate the documented role of language development in Theory of Mind (ToM). Currently there are two main views: one is that ToM tasks require some level of linguistic abilities and this is why there exists a relationship between the two domains. The second view is that language plays some role in the development of ToM concepts themselves. The authors here aimed to tease apart these two explanations by investigating the specificity of the response in the classic ToM brain regions on the premise that specificity is equivalent to functional maturity. In this sense, if a brain region 'for' a particular function is functionally mature, it responds to a narrow range of inputs, characteristic of its function. What they find here is that deaf children who are late signers show less specificity of the TPJ than early signers, and, since late signers are likely to have less language input than early signers, the inference is that language contributes to cortical specificity. I am generally positive about this paper and think it makes a nice contribution to a very tricky theoretical question. I follow their logic and though I am uncomfortable with the very small sample size upon which these analyses are based, I equally recognize that this sample is valuable. Thus, I do think that the results are likely to be an important contribution to the theoretical debate. That said, I have some comments and questions that I think the authors should deal with.

1. I found it difficult to figure out what they think is the relationship between cortical specialization and performance on behavioural tasks. They report that response selectivity did not correlate with ToM behavior and I am wondering why it doesn't? Late signing deaf kids do worse in ToM tasks, including 'minimal linguistic' tasks. Maybe the sample size is too small to find a relationship but then what is the role of this reduced specificity? The authors don't really talk about why they think that reduced selectivity would lead to worse performance (and even though they didn't find that relationship in this paper, the implication is that if language is important in the development of ToM, this immature selectivity is a factor).

2. The authors found no relationship in adults. Do we know that late signers eventually catch up such that no remnants of their impairment can be observed? If I remember correctly, Gary Morgan found that adults who learned to sign after the age of 10 years, showed lasting deficits in Theory of Mind use. Would then the hypothesis be that these very late signers would continue to show reduced selectivity into adulthood?

3. How do these data mesh with the apparently quite narrow selectivity of the TPJ region in infants (Hyde et al., 2018). In that study, the authors found that the TPJ was particularly responsive to a 'false belief' event and less responsive (if I remember correctly, not responsive above chance) to a 'true belief' or 'false belief that doesn't matter' event. This would seem to imply quite a lot of specificity is already there in very young infants – despite presumably no language comprehension, at least not of mental state terms. I don't know what the answer is, but I do think the authors should discuss this in their paper. I think the implication of the current manuscript should be that whatever infants are doing on infant-false-belief tasks, it isn't thinking about nuanced mental states like beliefs,

which (according to the current data) would presumably be expected to be language-dependent.

Reduced Neural Selectivity for Mental States in Deaf Children with Delayed Exposure to Sign Language

Tracking number: NCOMMS-19-19645A

Response to Reviewers

Thank you to the reviewers for their thoughtful comments and suggestions. We respond to each, below, using bold font. Edited portions of the manuscript text are highlighted.

Reviewer #1 (Remarks to the Author):
The manuscript reports data collected from deaf children who were either native signers or who had been exposed initially to sign only after varying amounts of time. The goal of the project is to determine whether delay of exposure (up to 7 years) to linguistic input influences theory of mind processing, both behaviorally and neurally. The focus is on determining the "mechanism of correlation" between language skills and theory of mind ability. Overall, behavioral data show delays on theory of mind tests given delays in initial exposure to language. Neurally, the right temporoparietal junction showed reduced response selectivity to theory of mind stimuli/tasks in the language delay sample of signers relative to the native signers. The authors argue that the data "…provide compelling evidence that linguistic experience facilitates developmental change in ToM specific representations, via the development of a selective brain region for ToM."

The authors are to be commended for pursuing this difficult study. As they note, the data are valuable. The results are interesting, if not surprising: language experience introduces complex concepts, which in turn develop understanding of mental states in others. This experiment highlights an ideal way to establish this effect. What is unclear is what the debate is actually about. As quoted above, the authors argue that their findings provide clear evidence of language influencing development of the neural underpinnings that support theory of mind abilities. The only alternative would be that theory of mind is somehow magically (innately) intact at birth. Does anyone make this argument?

Moreover, the "mechanism of correlation" that the authors purport to have determined seems uncontroversial: language experience is critical for the development of theory of mind abilities. Were there arguments to the contrary? It was not clear from the introduction, and that position seems hard to justify outside of a purely modular view of the brain. An important data point that might get at mechanism was the finding that age of ASL onset was significantly negatively correlated with standardized non-verbal IQ score in this sample (although the effect of age of ASL onset on the linguistic ToM task was significant when additionally including standardized non-verbal IQ as a covariate). This brings to mind recent findings by Matt Hall on executive function and other learning delays in those with delayed exposure to sign and/or spoken language (in the case of CI users). Although tangential to the theory of mind question being addressed here, that work might be useful to the authors in thinking about how to frame/interpret these data.

Thank you for this feedback.

We believe that the current study primarily speaks to an important (and more nuanced)

debate about *how* language experience impacts performance on ToM tasks. Though most would be comfortable saying that environmental and experiential factors shape ToM development, linguistic experience could plausibly play a superficial role in *expressing* ToM knowledge, rather than *constructing* ToM representations. This argument has been made particularly salient in recent years, given (controversial) evidence that even young infants pass false belief tasks when tested in non-linguistic formats. A strong interpretation of evidence from infants (which is certainly held by some) is that infants already have the conceptual repertoire and representational capacities to reason about others' beliefs, and that behavioral improvement on ToM tasks primarily reflects development of domain-general abilities (language, executive functions) that enables children to meet task demands.

We have more clearly articulated the debate that these data speak to in the Introduction:

> **"Traditional false belief tasks require children to comprehend linguistically sophisticated narratives and questions (e.g. *When Sally comes back where will she look first for her cookie?*). Consequently, performance on these false belief tasks reflects children's ability to follow, understand, and remember a linguistic narrative and question, and (typically) to form, select, and produce a linguistic response (Bloom & German, 2000). Linguistic abilities correlate with and predict children's performance on false belief tasks (Astington, 2006; Astington & Jenkins, 1999; Milligan, Astington, & Dack, 2007). Moreover, in a single testing session young children can fail linguistic false belief tasks and succeed on conceptually analogous non- or minimally linguistic false belief tasks (Clements & Perner, 1994; Geren, Snedeker, Shafto, & Geren, 2009; see also Wang, Ali, Frisson, & Apperly, 2016), which reduce linguistic task demands by showing the narrative through sequences of pictures or movies, and by measuring action predictions via eye gaze or pointing behaviors. Even very young children show precocious success on non- or minimally linguistic false belief tasks (He, Bolz, & Baillargeon, 2011; Onishi & Baillargeon, 2005; Scott, He, Baillargeon, & Cummins, 2012), suggesting that children's linguistic abilities may be a rate-limiting factor for performance on traditional ToM tasks.**
>
> **One possibility is therefore that linguistic *experience* primarily affects performance on ToM tasks indirectly, via a direct effect on the linguistic *abilities* that children need for most ToM tasks. This possibility is particularly salient given the controversial evidence that toddlers and even infants pass false belief tasks when presented in non-linguistic formats (Onishi & Baillargeon, 2005; Southgate, Senju, & Csibra, 2007; Buttelmann, Carpenter, & Tomasello, 2009; though see Barone, Corradi, & Gomila, 2019; Kulke & Rakoczy, 2018; Powell, Hobbs, Bardis, Carey, & Saxe, 2017). Some have claimed that human infants have the conceptual repertoire and representational capacities to reason about others' beliefs, and that subsequent behavioral improvement on traditional ToM tasks primarily reflects development of domain-general abilities, such as language and executive functions that enable children to meet task demands (Carruthers, 2013; Scott & Baillargeon, 2017).**

However, the representational capacities that are required to pass non-linguistic false belief tasks remain debated (Heyes, 2014; Southgate, 2018).

On the other hand, conversational experience may play a direct role in the construction of ToM concepts and representations. Children could learn to differentiate mental state concepts (e.g. *believe* vs. *know*, *want* vs. *hope*) from the way adults use mental state verbs in conversation (Ruffman, Slade, & Crowe, 2002; Gleitman, 1990). Conversations provide experience with linguistic structures that could be particularly important for ToM representations used during false belief tasks, like syntactic complement structures (de Villiers & de Villiers, 2009; de Villiers & Pyers, 2002; Hale & Tager-Flusberg, 2003; Lohmann & Tomasello, 2003; Ruffman et al., 2002). Even utterances that do not contain mental state verbs or syntactic complement structures (e.g. *Where are my keys?*) frequently provide evidence about another person's beliefs and desires, and the link between those mental states and observable behavior (Peterson & Siegal, 1999; Siegal & Peterson, 1994). Experience in linguistically rich conversational exchanges could thus directly support development of a more specific and accurate understanding of other minds (de Rosnay & Hughes, 2006; Dunn, Brown, Slomkowski, Tesla, & Youngblade, 1991; Slaughter & Peterson, 2012).

…

Competing hypotheses about the role of linguistic experience in ToM development make distinct predictions for the development of brain regions selective for ToM, and specifically, for RTPJ. If linguistic experience directly influences development of domain-specific ToM concepts, then delayed access to language may affect the development of selective responses in RTPJ. That is, instead of resembling responses in chronologically age-matched children, who have the same amount of biological maturation, the RTPJ response in delayed signers might most resemble responses in younger typically developing children, who have the same amount of linguistic experience. We predicted that the response in RTPJ in native signers would be similar to previously observed ToM-selective responses in age-matched hearing children. We then tested whether RTPJ responses would be less selective, as a function of delayed access to language. These specific neural predictions provide a novel and complementary way to investigate the role of linguistic experience in ToM development – which is not only theoretically significant, but also provides important information for parents, who must make difficult choices about how to ensure their child learns language."

We have additionally removed text that was tangential to this debate from the Introduction, for clarity.

The following two aspects of the data are surprising: 1) the remarkable similarities in performance on many measures across the two groups of signers, and 2) the lack of differences in theory of mind abilities in adult native vs. delayed signers. Regarding the first point, an example of an unexpected outcome is the following: although receptive ASL proficiency

increased with age, there was no difference in receptive ASL proficiency as a function of age of ASL onset. In fact, the delayed signers averaged (nonsignificantly) better performance: M(SE) proportion correct in delayed signers: .79(.02); native signers: .70(.04). That's surprising, isn't it?

In the current sample, the lack of a difference in receptive ASL proficiency between native and delayed signing children was not particularly surprising to us, for a few reasons: 1) only proficient ASL signers were recruited to participate in the study, and children were screened by a proficient ASL signer prior to participating (indeed – the entire testing session was conducted in ASL, and therefore required interacting with experimenters in ASL over the course of ~4 hours); 2) the delayed signing children are all considered to be "early" signers: the duration of delay ranged from .25 – 7 years, with a majority of children gaining access to ASL by age five years. All children had at least 3.5 years of exposure to ASL prior to participating; 3) the receptive ASL task may not be sensitive to subtle differences in ASL proficiency, or to differences in more complicated aspects of linguistic processing. The task that we selected is one of the few available standardized measures for assessing receptive ASL.

Likewise, the finding that the effect doesn't hold in the two types of adult signers indicates that this processing ability is remarkably plastic in a way that the comparison abilities (e.g., face recognition, visual word form processing) are not. The authors push that comparison with their fMRI findings, but the similarities among the adult signers challenges that comparison.

We did not observe any differences in the ToM neural response as a function of delayed access to language among our adult participants. Given that a large majority of our adult participants are "early" signers – that is, they received exposure to language by age seven years – this result could reflect a lack of enduring ToM delays among our adult participants. Of course it is also possible that our neural measure – response selectivity – is not sensitive to enduring ToM delays. We now address this comment in our Discussion section:

> **"All of our child participants, and a vast majority of our adult participants, received access to ASL after a relatively short delay (.25 – 7 years) – suggesting that even relatively short delays can delay the development of selective responses for ToM in RTPJ. From our results alone, it is unclear how much linguistic experience is sufficient to overcome this delay, or if there is a sensitive period for the impact of linguistic experience on ToM. Prior behavioral studies have found evidence for enduring ToM delays in adults who received access to language after longer delays (e.g., after age ten years; Gagne & Coppola, 2017; Morgan & Kegl, 2006; O'Reilly, Peterson, & Wellman, 2014), but there is also evidence that ToM delays can resolve with subsequent linguistic experience (Morgan & Kegl, 2006; Peterson, Wellman, & Liu, 2005; Peterson, Wellman, & Slaughter, 2012; Pyers & Senghas, 2009). Here, we did not observe an enduring effect of delayed access to language on RTPJ selectivity in adults. Additional work is needed to determine whether there are prolonged effects of delayed access to language on more sophisticated aspects of ToM reasoning (O'Reilly et al., 2014), and/or more fine-grained aspects of the neural response (e.g., the organization of spatial response patterns within RTPJ). "**

More generally, the use of the dorsal medial prefrontal cortex as a comparison region is never justified/explained (nor is the acronym even introduced; it just appears without the full name of the region). This is one example of many aspects of the manuscript's structure that are problematic, making the logic of the authors' argument hard to follow. These are (indeed!) valuable data, but the exposition makes them harder to appreciate. It is not until discussion that the different mechanistic arguments become clear (or clearer).

Thank you for pointing this out – this mistake was a consequence of reorganizing the manuscript content. We now justify our focus on the DMPFC in addition to the RTPJ, and introduce this region by its full name prior to using the abbreviation:

> **"We planned to use established experimental analysis protocols to measure response selectivity in RTPJ in deaf children and adults as a function of the length of delay prior to first exposure to a sign language, and pre-registered analyses via the Open Science Framework (OSF; https://osf.io/kyu3f/?view_only=35949e8028f743bc973932fe3adf7831).**
>
> **However, because this is the first fMRI study of ToM in d/Deaf individuals, and because this dataset was exceedingly difficult to collect, and as such, is rare and precious, we additionally pre-registered analyses that tested for effects of age of ASL onset on a broad array of other potential metrics for the development of ToM brain regions (pre-registration: https://osf.io/kyu3f/?view_only=35949e8028f743bc973932fe3adf7831). Specifically, while prior evidence suggested that effects might be localized to the RTPJ (summarized above), our planned analyses additionally focused on development in dorsomedial prefrontal cortex (DMPFC), given some prior evidence that DMPFC development correlates with behavioral ToM performance in children (Bowman, Dodell-Feder, Saxe, & Sabbagh, 2019; Gweon, Dodell-Feder, Bedny, & Saxe, 2012; Sabbagh, Bowman, Evraire, & Ito, 2009). Additionally, we measured responses during "Non-Sign" stimuli – which were visually similar to the ASL stories, but lacked higher-level linguistic features (semantic meaning and syntax). This condition enabled us to analyze an array of metrics for the development of language brain regions (pre-registration: https://osf.io/8cmt4/?view_only=fd6a8bc69f1041ae8ec183ea8221771b). We also included a non-linguistic movie-viewing fMRI experiment. The movie-viewing experiment was not designed or selected to be conceptually analogous to the ASL story task. Rather, this experiment was selected because it is a short, engaging, and non-linguistic paradigm that evokes rich mental state inferences, recruits ToM brain regions (Jacoby, Bruneau, Koster-Hale, & Saxe, 2016; Richardson, Lisandrelli, Riobueno-Naylor, & Saxe, 2018), and could plausibly be completed along with the ASL task within the time constraints inherent to pediatric neuroimaging studies. This experiment was analyzed using identical methods to a prior study on ToM brain region development (Richardson et al., 2018). These additional planned, but exploratory, measures helped ensure that real but unpredicted effects wouldn't go unnoticed in such a valuable dataset."**

In short, the importance of the data is clear, but the presentation needs work.

We appreciate that the reviewer recognizes the importance of the data, and thank the reviewer for their thoughtful comments. We have re-organized and re-written for clarity, and believe that these edits have strengthened the manuscript.

Reviewer #2 (Remarks to the Author):

The current study investigates the effect of delayed exposure to sign language (ASL) on ToM performance and neural signature in children and adults born deaf. The main findings are that delayed access to ASL was associated with impaired performance and reduced functional selectivity of ToM brain regions in linguistic, but not in non-linguistic ToM tasks. The effects of delayed language exposure were only present in the children but not in the adult group. In addition, lateralization of ToM and language responses and inter-region correlations within and across the ToM and language network were analyzed, but did not show any significant effect of ASL onset. The authors conclude that these results support the view that language facilitates the functional specialization of ToM regions and is intrinsically related to ToM regions rather than merely to the expression of ToM abilities in ToM tasks.

The study addresses a very relevant question testing a scarce population of deaf children with (and without) delayed access to ASL, which is particularly well-suited to address the role of language for ToM development. Despite the small number of participants (n = 12 children with delayed ASL access), in particular, the MRI data of these children therefore constitute a very rare and valuable data set. The pattern of results, however, are relatively complex, so that I do not think that the strong conclusions of a direct causal relation of language exposure on ToM development drawn by the authors are supported. In addition, I have a number of methodological concerns and questions explained below.

1) Conclusions of a direct influence are too strong
Given the complexity of the results and the correlational nature of the findings, the authors' conclusion that early language input directly facilitates ToM development appears to be too strong.

First of all, given the correlational nature of the relation of ASL onset and ToM performance and selectivity, there could be other mediating factors, such as, social background, presence of older siblings or any other cognitive abilities that are likely to be related to both variables.

We have added this caveat to the Discussion section:

> **"Future research is also necessary to determine whether other factors plausibly related to linguistic experience and ToM development (e.g., social experience, presence of siblings, other cognitive abilities) mediate the correlation between these two variables, to test which aspects of linguistic experience (e.g., mental state vocabulary (Hale & Tager-Flusberg, 2003), syntactic complement structures (de**

> **Villiers & de Villiers, 2009; de Villiers & Pyers, 2002; Hale & Tager-Flusberg, 2003; Lohmann & Tomasello, 2003; Ruffman et al., 2002), conversational turns (Morgan et al., 2014) promote RTPJ selectivity (Bianco, Lecce, & Banerjee, 2016; Lecce, Bianco, Demicheli, & Cavallini, 2014a; Lecce, Bianco, Devine, Hughes, & Banerjee, 2014b; Lohmann & Tomasello, 2003), and to characterize whether and which other aspects of experience influence RTPJ development (Hughes & Devine, 2016)."**

Secondly, if language exposure matters for the development of ToM concepts rather than for task comprehension and expression, ASL onset should be related to both the verbal and the minimally verbal task. The authors, however, only found a relation with the verbal task.

The minimally verbal behavioral task did not include the hardest ToM concepts. The behavioral data are difficult to interpret alone precisely because the items that language-delayed signers performed worse on are both conceptually and linguistically hard. We make this argument in the Discussion, as it helps to make clear why our neuroimaging data are particularly useful:

> **"Consistent with the hypothesis that language abilities can limit or facilitate ToM task performance, we found that delayed access to ASL led to deficits on a linguistic ToM task. When ToM concepts were tested in a minimally linguistic format, native and delayed signers' performance was matched. The observed interaction of ASL delay and task format was also observed in a subset of conceptually analogous items testing moral reasoning, and is consistent with some prior observations that delayed language affects performance on linguistic more than minimally linguistic ToM tasks (Figueras-Costa & Harris, 2001; Gale, De Villiers, De Villiers, & Pyers, 1996; Schick, De Villiers, De Villiers, & Hoffmeister, 2007).**
>
> **A simple interpretation might therefore be that the complex language of ToM tasks can mask true conceptual abilities, especially in children with delayed access to language. However, we do not favor this interpretation. In the current study, all children in the sample, including those with delayed access to language, showed an overall *benefit* of the linguistic format on ToM performance. That is, we observed that all groups of children were actually better able to reason about false beliefs when the scenario was presented with a linguistic narrative, than when presented via pictures alone. This pattern of evidence is consistent with a recent study in which deaf children, like hearing children, performed better on false belief questions that required providing linguistic explanations of behavior, compared to those that required making linguistically simpler forced-choice predictions (Peterson & Wellman, 2018). Thus linguistic narratives may have actually facilitated children's ability to encode, mentally manipulate, and retrieve complex mental state concepts (Frank, Everett, Fedorenko, & Gibson, 2008). Relatedly, consistent with some prior studies (de Villiers & Pyers, 2001; Hughes, 1998; Schick et al., 2007; Woolfe, Want, & Siegal, 2002; though see Grosse Wiesmann, Friederici, Singer, & Steinbeis, 2017; Low, 2010), children's receptive ASL proficiency was highly correlated with performance on the *minimally* linguistic ToM task. Children with proficient language skills may spontaneously create linguistic narratives to help them encode**

the sequence of non-linguistic images. Overall, these results argue against interpreting performance on the linguistic ToM task as an underestimate of children's ToM competence, masked by limited language abilities, at least for the current sample of children who are highly proficient signers. Instead, we hypothesize that the effect of delayed exposure to language was stronger on the linguistic task at least in part because it included the most tests of advanced ToM. Because of the limitations of the format, the most sophisticated ToM concepts (understanding lies, sarcasm, and second-order false beliefs) were not tested in the minimally linguistic task. Children with delayed access to language show improvement over time on measures of ToM (Peterson & Wellman, 2018); in our relatively old and linguistically proficient sample, residual ToM delays may only be seen among the most sophisticated and later-acquired concepts.

In sum, the current behavioral results replicate prior findings that delayed access to language can delay children's performance on ToM tasks (Figueras-Costa & Harris, 2001; Gale et al., 1996; Peterson et al., 2005; 2012; Peterson & Siegal, 1999; Peterson & Wellman, 2018; Schick et al., 2007; Schick & Hoffmeister, 2001; Woolfe et al., 2002), even in children who are fluent in ASL. However, the behavioral results cannot resolve whether linguistic experience mainly facilitates task performance, or promotes development in ToM reasoning per se.

Our neuroimaging evidence offers independent and complementary insight into this question…"

The authors argue that ASL onset and its relation with selectivity were independent of language proficiency as measured by the ASL-RST. However, the relation could have resulted from more complex linguistic aspects of the task (not tested for in the test of ASL proficiency), as for example pragmatics, in particular given that the authors claim that the correlation might have been driven by "hard" items of the verbal ToM task, including sarcasm, lies, etc.

The reviewer is correct that the receptive ASL task may not have been sensitive to ongoing delays in more complex or later developing aspects of language. Pragmatics is an interesting example to highlight – as it sits at the intersection of ToM and language. Delayed development in pragmatics could reflect delayed ToM development or delayed linguistic development or both. Indeed, our behavioral ToM task includes some items that fall under the description of pragmatics, including reasoning about irony and sarcasm – and delays observed on these items difficult to interpret, because they are complicated both in terms of language and in terms of ToM concepts. (Note that there was no effect of delayed access to language on control items on the behavioral ToM task – suggesting that all children could at least follow the narrative and answer simple control questions using ASL).

We do not believe that the correlation between RTPJ selectivity and ASL onset could have resulted from more complex linguistic aspects of the fMRI task. The ASL stories in the fMRI task were written for children. We recruited individuals who reported using ASL in their everyday lives, completed the entire (4+ hour-long) testing session in ASL, and

showed ASL proficiency on a basic test of receptive ASL. Our fMRI experiment enabled us to measure responses in language brain regions in addition to ToM brain regions, and there were no differences in the responses in canonical language brain regions as a function of delayed access to language. While null results should of course be treated with caution - we did not observe any neural evidence that indicated greater processing difficulty of the ASL stories in children with delayed access to language (i.e., in language cortex, or in the multiple demand network). Additionally, all children recruited RTPJ to process the mental state stimuli; there were no observable differences in the magnitude of response to mental state stimuli as a function of delayed access to language. Instead, we observed *less selective* responses in the RTPJ of children with delayed access to language (high responses not only to descriptions of characters' mental states, but also to more general information about characters). The difference observed between RTPJ responses in native and delayed signers resembles that previously observed between older and younger children (Gweon et al., 2012; Saxe et al., 2009). Together, these results make it unlikely that the correlation between age of ASL onset and RTPJ selectivity reflects uncaptured linguistic complexity.

We have edited the Introduction and Discussion in order to address this comment, and the comment that immediately follows. See the edited text below ("In many cortical regions…").

In addition, the authors argue that a poorer comprehension of mental aspects would have caused lower activation for the mental state condition per se rather than lower selectivity (l.435, l.456). However, in contrast to these predictions, less advanced linguistic abilities might also predict a less refined differentiation of mental versus social contents and therefore potentially less differentiated activity patterns (exactly as observed in the current study).

We have attempted to clarify our prediction in the Introduction:

> **"In many cortical regions, increasing selectivity during childhood occurs by the suppression of responses to non-preferred stimuli. For example, selectivity of the fusiform face area develops through the suppression of responses to non-face objects; this suppression correlates with performance on face recognition tasks (Cantlon, Pinel, Dehaene, & Pelphrey, 2010; Golarai et al., 2007; Gomez et al., 2017). Selectivity of the visual word form area develops through the suppression of responses to faces (Cantlon et al., 2010); this suppression predicts literacy and reading expertise (Dehaene et al., 2010; Dehaene-Lambertz, Monzalvo, & Dehaene, 2018). Similarly, selectivity of the RTPJ develops through the suppression of responses to other (non-mentalistic) social information, and correlates with performance on ToM tasks (Gweon et al., 2012). The development of a spatially localized neural population with shared selectivity for mental state reasoning may facilitate finer-grained distinctions between mental state concepts (Kanwisher, 2010; Koster-Hale & Saxe, 2013). While the development of selective brain regions for perception of faces or words appears to depend on extensive experience with those visual categories (Arcaro, Schade, Vincent, Ponce, & Livingstone, 2017; Dehaene et al., 2010; Dehaene, Cohen, Morais, & Kolinsky, 2015), there is currently no evidence concerning the degree to which development of selective responses in RTPJ is**

influenced by environmental input, nor about which aspects of the environment drive development of selective responses in this region.

Competing hypotheses about the role of linguistic experience in ToM development make distinct predictions for the development of brain regions selective for ToM, and specifically, for RTPJ. If linguistic experience directly influences development of domain-specific ToM concepts, then delayed access to language may affect the development of selective responses in RTPJ. That is, instead of resembling responses in chronologically age-matched children, who have the same amount of biological maturation, the RTPJ response in delayed signers might most resemble responses in younger typically developing children, who have the same amount of linguistic experience. We predicted that the response in RTPJ in native signers would be similar to previously observed ToM-selective responses in age-matched hearing children. We then tested whether RTPJ responses would be less selective, as a function of delayed access to language. These specific neural predictions provide a novel and complementary way to investigate the role of linguistic experience in ToM development – which is not only theoretically significant, but also provides important information for parents, who must make difficult choices about how to ensure their child learns language."

We additionally edited the relevant section of the Discussion for clarity:

"We found that in native signing children, and all adults, the RTPJ showed selective responses to stories about mental states in the ASL story task. Delayed signing children showed high responses to Mental stories, but the response in their RTPJ was also high for non-mentalistic social information – like physical appearances and enduring relationships. Moreover, the length of the delay prior to access to ASL correlated with delayed selectivity of RTPJ (despite current ASL proficiency and controlling for age). This profile of response is difficult to explain in terms of general difficulty with constructing or accessing linguistic representations. We recruited children who reported using ASL in their everyday lives and who could complete an entire study (4+ hours) in ASL, and confirmed that there were not differences in performance on a basic task of ASL proficiency as a function of delayed access to language at the time of the study. Moreover, all children showed high RTPJ responses to the Mental stories, suggesting that the semantic content of these stories was successfully extracted. Rather than resembling the response in age-matched children, who have the same amount of biological maturation, the response profile in RTPJ in delayed signing children resembles that previous observed in young children (Gweon et al., 2012; Saxe, Whitfield-Gabrieli, Scholz, & Pelphrey, 2009), who have a similar amount of linguistic experience. Also, we did not observe any effect of delayed access to ASL on activity in language-related regions, nor did we observe additional recruitment of brain regions that would indicate greater difficulty processing the linguistic stimuli in delayed signers (e.g., language brain regions, or the multiple demand network); though null results in fMRI are difficult to interpret and should be treated with caution. Overall, the pattern of results

> **suggests that linguistic experience has a direct impact on domain-specific ToM development."**

Finally, the authors argue that there wasn't more activity in language regions in children with later ASL onset which would have indicated that these children struggled more with the task. However, it is not possible to interpret a null-result to conclude the opposite, namely that they did not struggle more, in particular in such a small sample and so widely defined language ROIs (compared to the much more restrictive ToM ROIs, see comment below).

Of course the reviewer is correct. Null results are difficult to interpret and should be interpreted with caution. But we still believe it is important to present the data and results that we have on language responses - this experiment enabled us to measure language responses, we pre-registered a set of analyses to characterize language responses, and we expect readers to be interested in these results. We do now remind readers that null results must be treated with caution, in the Discussion section (see blue highlights in excerpt above).

The complexity of the results and ambiguity in their interpretation need to be made clearer in the abstract, conclusions, and throughout the manuscript (e.g., in the last sentence of the abstract and in the Discussion in lines 377, 433, 504), and these arguments need to be refined.

Thank you. We have re-written the abstract and manuscript to highlight the main prediction that this experiment tests, which is that if linguistic experience directly impacts ToM development, we should see neural differences as a function of linguistic experience in the development of selective responses in RTPJ. We highlight our rationale for this prediction, and explain our inclusion of other tests and measures (e.g., selectivity in DMPFC, responses in language brain regions, and responses in ToM regions during a movie experiment). We have attempted to transparently report our hypotheses and planned and exploratory tests. We also attempt to make a clear, simple argument given our results, while also providing a thorough description of all of the results. In some cases, this meant moving interesting but tangential results and discussion to the Supplementary Information.

2) Relatedly, if early language deprivation impacts ToM development, how do the authors explain that there was no effect of delayed ASL onset on selectivity for ToM in adults?

This is a null effect that must be interpreted with caution. But one interpretation is that lengthy exposure to ASL can compensate for initial delayed access to language, for the development of selective RTPJ responses (of course – there may be prolonged differences in RTPJ responses based on delayed access to language that the current experiment and approach are insensitive to). We address this comment in the following paragraph in the Discussion:

> **"All of our child participants, and a vast majority of our adult participants, received access to ASL after a relatively short delay (.25 – 7 years) – suggesting that even relatively short delays can delay the development of selective responses for ToM in**

RTPJ. From our results alone, it is unclear how much linguistic experience is sufficient to overcome this delay, or if there is a sensitive period for the impact of linguistic experience on ToM. Prior behavioral studies have found evidence for enduring ToM delays in adults who received access to language after longer delays (e.g., after age ten years; Gagne & Coppola, 2017; Morgan & Kegl, 2006; O'Reilly et al., 2014), but there is also evidence that ToM delays can resolve with subsequent linguistic experience (Morgan & Kegl, 2006; Peterson et al., 2005; 2012; Pyers & Senghas, 2009). Here, we did not observe an enduring effect of delayed access to language on RTPJ selectivity in adults. Additional work is needed to determine whether there are prolonged effects of delayed access to language on more sophisticated aspects of ToM reasoning (O'Reilly et al., 2014), and/or more fine-grained aspects of the neural response (e.g., the organization of spatial response patterns within RTPJ)."

3) The introduction would benefit from a better theoretical embedding of different ways how language might influence ToM development (e.g. understanding conversations in their environment might allow picking up on mental concepts, but also language might provide the framework to think about these abstract concepts as e.g. argued by de Villiers or Harris and colleagues). E.g. line 32 ff.: Not only complement structures and mental verbs were shown to relate to ToM, but also more general linguistic abilities, syntax as well as vocabulary, and there are controversial findings and theories on the direction of effects (see e.g. Milligan et al. 2007).

We try to (succinctly) highlight the various ways that linguistic experience could directly impact ToM development in the Introduction:

"A majority of studies on ToM use the false belief task, which involves asking participants to predict the action of a character that has a false belief (e.g. *Sally left her cookie in the drawer, but when Sally wasn't looking, Anne moved the cookie to the desk*). Experience with both mental state vocabulary (e.g., "*think*", "*know*") and syntactic complement structures (e.g., "*Sally thinks that the cookie is in the drawer*") predicts and drives early performance on false belief tasks (Lohmann & Tomasello, 2003; Ruffman et al., 2002), even when controlling for a child's current language abilities[9]. Nevertheless, it remains unclear whether improved performance on false belief tasks reflects more sophisticated ToM representations, or an improved ability to meet linguistic task demands.

Traditional false belief tasks require children to comprehend linguistically sophisticated narratives and questions (e.g. *When Sally comes back where will she look first for her cookie?*). Consequently, performance on these false belief tasks reflects children's ability to follow, understand, and remember a linguistic narrative and question, and (typically) to form, select, and produce a linguistic response (Bloom & German, 2000). Linguistic abilities correlate with and predict children's performance on false belief tasks (Astington, 2006; Astington & Jenkins, 1999; Milligan et al., 2007). Moreover, in a single testing session young children can fail linguistic false belief tasks and succeed on conceptually analogous non- or minimally linguistic false belief tasks (Clements & Perner, 1994; Geren et al., 2009; see also

Wang et al., 2016), which reduce linguistic task demands by showing the narrative through sequences of pictures or movies, and by measuring action predictions via eye gaze or pointing behaviors. Even very young children show precocious success on non- or minimally linguistic false belief tasks (He et al., 2011; Onishi & Baillargeon, 2005; Scott et al., 2012), suggesting that children's linguistic abilities may be a rate-limiting factor for performance on traditional ToM tasks.

One possibility is therefore that linguistic *experience* primarily affects performance on ToM tasks indirectly, via a direct effect on the linguistic *abilities* that children need for most ToM tasks. This possibility is particularly salient given the controversial evidence that toddlers and even infants pass false belief tasks when presented in non-linguistic formats (Onishi & Baillargeon, 2005; Southgate et al., 2007; Buttelmann et al., 2009; though see Barone et al., 2019; Kulke & Rakoczy, 2018; Powell et al., 2017). Some have claimed that human infants have the conceptual repertoire and representational capacities to reason about others' beliefs, and that subsequent behavioral improvement on traditional ToM tasks primarily reflects development of domain-general abilities, such as language and executive functions that enable children to meet task demands (Carruthers, 2013; Scott & Baillargeon, 2017). However, the representational capacities that are required to pass non-linguistic false belief tasks remain debated (Heyes, 2014; Southgate, 2018).

On the other hand, conversational experience may play a direct role in the construction of ToM concepts and representations. Children could learn to differentiate mental state concepts (e.g. *believe* vs. *know*, *want* vs. *hope*) from the way adults use mental state verbs in conversation (Ruffman et al., 2002; Gleitman, 1990). Conversations provide experience with linguistic structures that could be particularly important for ToM representations used during false belief tasks, like syntactic complement structures (de Villiers & de Villiers, 2009; de Villiers & Pyers, 2002; Hale & Tager-Flusberg, 2003; Lohmann & Tomasello, 2003; Ruffman et al., 2002). Even utterances that do not contain mental state verbs or syntactic complement structures (e.g. *Where are my keys?*) frequently provide evidence about another person's beliefs and desires, and the link between those mental states and observable behavior (Peterson & Siegal, 1999; Siegal & Peterson, 1994). Experience in linguistically rich conversational exchanges could thus directly support development of a more specific and accurate understanding of other minds (de Rosnay & Hughes, 2006; Dunn et al., 1991; Slaughter & Peterson, 2012).

The role of language in facilitating ToM development is specifically relevant for understanding ToM development in children who are d/Deaf (Note that the capitalized word "Deaf" refers to the cultural and linguistic minority group, and the lower-case "deaf" refers to the audiological status. We use "deaf" from this point forward because we were often unable to distinguish between the two (e.g., in young children)). Many deaf or hard-of-hearing children are at risk of not learning any language in early childhood because they have limited auditory access to spoken language, and their families do not know sign language at the time of birth (Mitchell

& Karchmer, 2004). Deaf children with delayed exposure to sign language show delays in ToM, relative to hearing children and relative to deaf children exposed to sign language from infancy. Interestingly, the delay in ToM appears not to be fully explained by the linguistic demands of ToM tasks. Delayed exposure to sign language affects performance on linguistic and minimally linguistic false belief tasks (Figueras-Costa & Harris, 2001; Gale et al., 1996; Peterson et al., 2005; 2012; Peterson & Siegal, 1999; Peterson & Wellman, 2018; Schick et al., 2007; Schick & Hoffmeister, 2001; Woolfe et al., 2002), and even on non-linguistic anticipatory looking paradigms (Meristo et al., 2012). Even once children are proficient in a sign language, there is an apparent lag before ToM performance catches up. Also, the effects on ToM are correlated not just with the child's language abilities, but specifically with the richness of maternal mental state language: when hearing parents learn a sign language as a second language, they vary in their use of mental state language which in turn predicts ToM performance among deaf children (Moeller & Schick, 2006).

In sum, it remains an open question whether linguistic experiences influence ToM mainly indirectly, by enhancing children's linguistic abilities to succeed on the tasks, or also directly, by supporting children's development of ToM concepts. In order to address this question, we provide a complementary approach to existing behavioral research, leveraging recent discoveries about the development of ToM-related responses in children's brains. …"

4) References infant ToM:
Instead of citing 3 (randomly?) selected studies of infant ToM (references 18-20), I would suggest to cite one or two review articles that give an overview of all non-verbal infant ToM studies, maybe from different perspectives including the current controversy (e.g. new meta-analysis by Barone, Corradi, & Gomila, 2019).

"Moreover, in a single testing session young children can fail linguistic false belief tasks and succeed on conceptually analogous non- or minimally linguistic false belief tasks (Clements & Perner, 1994; Geren et al., 2009; see also Wang et al., 2016), which reduce linguistic task demands by showing the narrative through sequences of pictures or movies, and by measuring action predictions via eye gaze or pointing behaviors. Even very young children show precocious success on non- or minimally linguistic false belief tasks (He et al., 2011; Onishi & Baillargeon, 2005; Scott et al., 2012), suggesting that children's linguistic abilities may be a rate-limiting factor for performance on traditional ToM tasks.

One possibility is therefore that linguistic *experience* primarily affects performance on ToM tasks indirectly, via a direct effect on the linguistic *abilities* that children need for most ToM tasks. This possibility is particularly salient given the controversial evidence that toddlers and even infants pass false belief tasks when presented in non-linguistic formats (Onishi & Baillargeon, 2005; Southgate et al., 2007; Buttelmann et al., 2009; though see Barone et al., 2019; Kulke & Rakoczy, 2018; Powell et al., 2017). Some have claimed that human infants have the

conceptual repertoire and representational capacities to reason about others' beliefs, and that subsequent behavioral improvement on traditional ToM tasks primarily reflects development of domain-general abilities, such as language and executive functions that enable children to meet task demands (Carruthers, 2013; Scott & Baillargeon, 2017). However, the representational capacities that are required to pass non-linguistic false belief tasks remain debated (Heyes, 2014; Southgate, 2018).

5) References neural basis of ToM:
Line 118 ff.: The authors write that ToM and language regions are clearly distinct. The IFG and posterior superior temporal regions as well as the structural connection between the two regions have been shown to be involved in both (e.g. Schurz et al. 2015; Grosse Wiesmann et al. 2017)

We have edited the manuscript to focus on the development of selective responses for ToM. Our hypotheses focus on brain regions that respond preferentially to process mental states (e.g., planned analyses in RTPJ and DMPFC; and exploratory analyses in LTPJ, PC, and M/VMPFC). We describe results from prior literature characterizing the selective functional responses in these brain regions in the Introduction:

> **"Human adults and children recruit a specific network of brain regions when reasoning about the minds of others, including bilateral temporoparietal junction, precuneus, and medial prefrontal cortex (Adolphs, 2009; Koster-Hale & Saxe, 2013). While many brain regions are recruited to process narratives and movies, these brain regions (the "ToM network") show high hemodynamic responses to linguistic narratives (Fletcher et al., 1995; Saxe & Kanwisher, 2003), non-linguistic cartoons (Gallagher et al., 2000; Sommer et al., 2007), and movies (Jacoby et al., 2016) that evoke considerations of characters' mental states, relative to non-mentalistic control stimuli. When children reason about minds, they recruit this same network of brain regions (Gweon et al., 2012; Sabbagh et al., 2009). As children get older, the 'ToM network' becomes increasingly functionally distinct (Richardson, 2018; Richardson et al., 2018) and structurally connected (i.e., via myelination of white matter tracts (Grosse Wiesmann, Schreiber, Singer, Steinbeis, & Friederici, 2017)). Additionally, the right temporoparietal junction (RTPJ) becomes particularly selective for mental state content (Gweon et al., 2012; Saxe et al., 2009), such that by adulthood, it preferentially responds when people think about a person's beliefs and desires, relative to other meta-representations like photographs or maps (Dodell-Feder, Koster-Hale, Bedny, & Saxe, 2011; Saxe & Kanwisher, 2003), other internal states of humans like physical pain (Bruneau, Pluta, & Saxe, 2012; Jacoby et al., 2016) or bodily sensations (Lombardo et al., 2010; Saxe & Powell, 2006; Spunt, Kemmerer, & Adolphs, 2015), and other social information like a person's appearance or place of origin (Mitchell, Banaji, & Macrae, 2005; Saxe & Powell, 2006)."**

In the data presented in this manuscript, these brain regions respond more to process linguistic stimuli that describe mental states relative to linguistic stimuli that do not (Mental > Physical stories). We have added Figure 2 (and Table 1) to the main text in order

to illustrate this result, and have revised Figure S2 to include all ToM brain regions:

Figure 2:

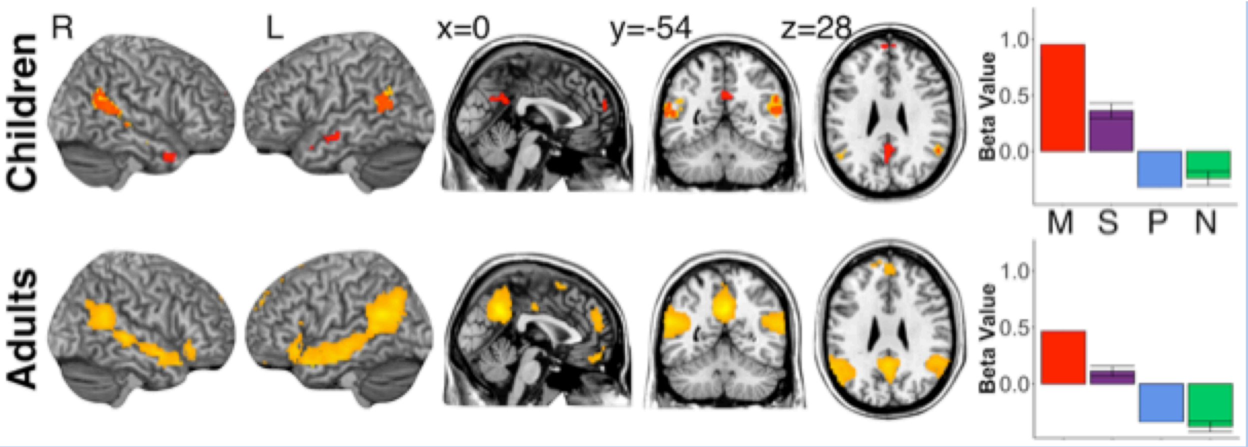

Figure S2

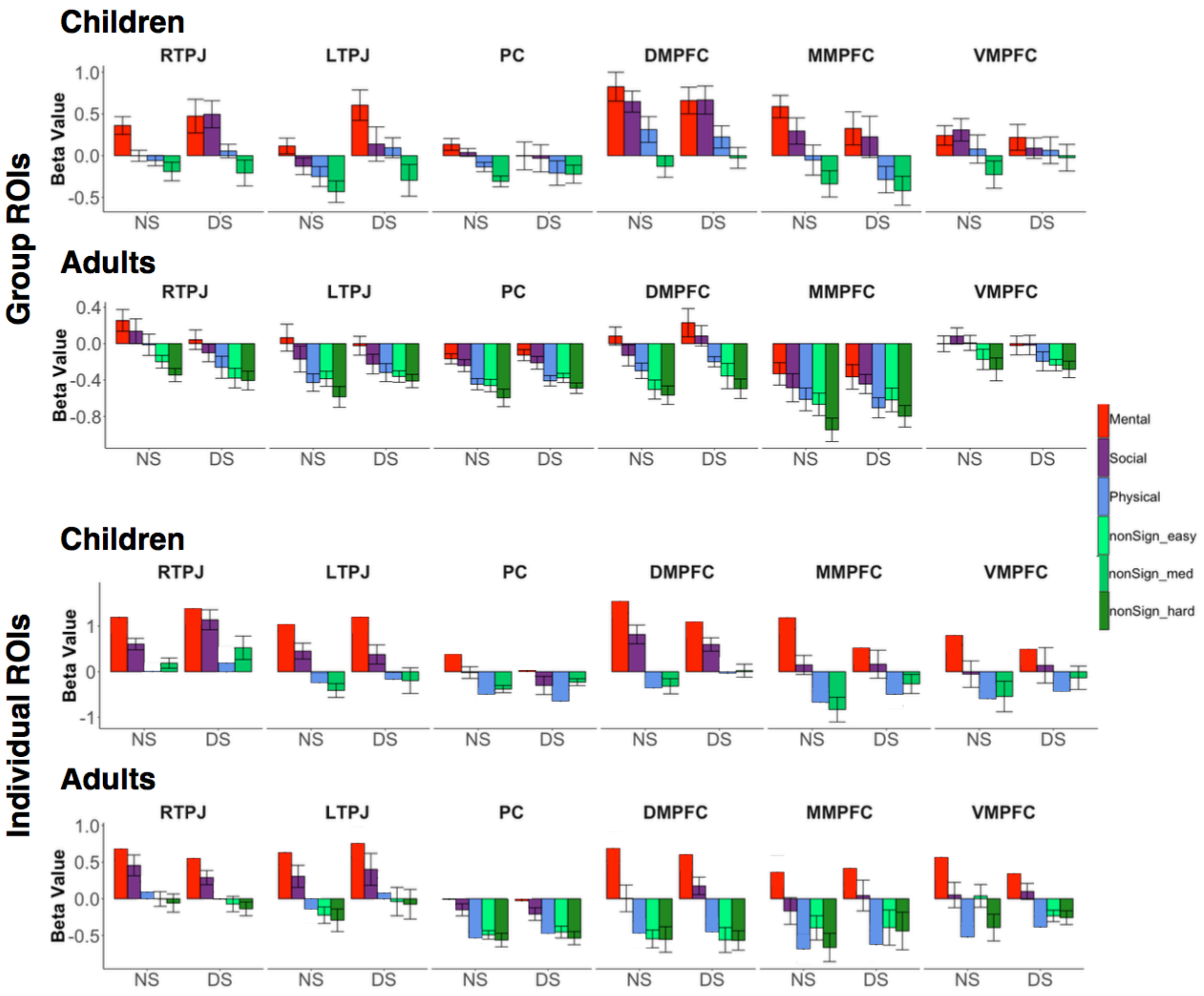

Line 119 ff.: The authors only mention the rTPJ being involved in ToM. Given that ToM tasks quite consistently activate a bilateral network of multiple brain regions (e.g., Schurz et al., 2015), I would suggest to mention all the regions of the ToM network.

The prior version of the manuscript highlighted the RTPJ, but explicitly noted it as part of a network of brain regions (referred to as the "ToM network"). However, we have re-written this section and now include the full names of the regions we are referring to. This paragraph now reads:

> **"Human adults and children recruit a specific network of brain regions when reasoning about the minds of others, including bilateral temporoparietal junction, precuneus, and medial prefrontal cortex (Adolphs, 2009; Koster-Hale & Saxe, 2013). While many brain regions are recruited to process narratives and movies, these brain regions (the "ToM network") show high hemodynamic responses to linguistic narratives (Fletcher et al., 1995; Saxe & Kanwisher, 2003), non-linguistic cartoons (Gallagher et al., 2000; Sommer et al., 2007), and movies (Jacoby et al.,**

> **2016) that evoke considerations of characters' mental states, relative to non-mentalistic control stimuli. When children reason about minds, they recruit this same network of brain regions (Gweon et al., 2012; Sabbagh et al., 2009). As children get older, the 'ToM network' becomes increasingly functionally distinct (Richardson, 2018; Richardson et al., 2018) and structurally connected (i.e., via myelination of white matter tracts (Grosse Wiesmann, Schreiber, Singer, Steinbeis, & Friederici, 2017)). Additionally, the right temporoparietal junction (RTPJ) becomes particularly selective for mental state content (Gweon et al., 2012; Saxe et al., 2009), such that by adulthood, it preferentially responds when people think about a person's beliefs and desires, relative to other meta-representations like photographs or maps (Dodell-Feder, Koster-Hale, Bedny, & Saxe, 2011; Saxe & Kanwisher, 2003), other internal states of humans like physical pain (Bruneau, Pluta, & Saxe, 2012; Jacoby et al., 2016) or bodily sensations (Lombardo et al., 2010; Saxe & Powell, 2006; Spunt, Kemmerer, & Adolphs, 2015), and other social information like a person's appearance or place of origin (Mitchell, Banaji, & Macrae, 2005; Saxe & Powell, 2006)."**

Line 125 ff.: Concerning the children's literature, I would suggest to add that similar network of brain regions is involved in the development of ToM in early childhood (e.g. Richardson et al. 2017; Sabbagh et al. 2009, Grosse Wiesmann et al. 2017) in addition to an increase of selectivity in the rTPJ and other regions.

We previously cited the Richardson & Sabbagh papers; we have added both to the initial description of prior work with children, in addition to the Grosse Wiesmann paper (see paragraph above).

6) Specific role of the rTPJ?
Also in the results, the authors suggest a specific role of the selectivity of rTPJ rather than the DMPFC. Table 1 shows no significant effect of ROI (for the ToM ROIs) nor is there a significant interaction of ASL onset and ROI. What was the rational behind conducting post-hoc analyses in each ROI separately then?

Our goal is to provide a clear and comprehensive description of the data. As described by the reviewer, we report that there is no effect of ROI, nor is there a significant interaction of ASL onset and ROI. We additionally note that statistical tests conducted per ROI were post-hoc. We conducted these tests because the visualizations of the data (Figure 3, Figure S2) suggested that the effect may be more pronounced in RTPJ – and we believe that this information is important for our readers to know and consider, especially given prior evidence about the different functional response profiles and developmental trajectories in these two regions. In particular, prior evidence suggests that RTPJ (and not DMPFC) is particularly selective for processing mental states in adults (Dodell-Feder et al., 2011; Saxe & Kanwisher, 2003; Bruneau et al., 2012; Jacoby et al., 2016; Lombardo et al., 2010; Saxe & Powell, 2006; Spunt et al., 2015), and prior studies find that responses in RTPJ (but not DMPFC) become increasingly selective for mental states during childhood (Gweon et al., 2012; Saxe et al., 2009).

7) Definition of the ROIs

The supplemental material suggests that the reported pattern of results also holds for ROIs defined on a group level. Given that this ROI definition is independent of individual activation in the mental state condition, I find these results more convincing and would suggest to report these in the main body of the paper (potentially instead of the functionally defined ROIs).

Respectfully, we have opted to keep results from individual ToM regions of interest in the main text. We pre-registered conducting analyses in individual ROIs as our primary, main analyses – in part because we wanted to be sure to sensitively capture functional responses in our individual participants (because group regions of interest are not tailored to individual functional response profiles, they can underestimate or even miss relevant activation in individual participants). This feels particularly important in the current study and sample, given our results concern reduced response selectivity. Using individual regions of interest helps to ensure we are not underestimating response selectivity in individual participants.

It is also worth noting that our selectivity index is independent from ROI definition. Essentially our analyses ask: in ToM regions (which fall within the search spaces, and respond more to Mental > Physical stories), how high is the response to the Social condition? This approach is suited to test our hypothesis about response selectivity, is directly based on prior work (Gweon et al., 2012; Saxe et al., 2009), and was pre-registered (https://osf.io/kyu3f/?view_only=35949e8028f743bc973932fe3adf7831).

Visualizations of responses in group ROIs and full statistics of response selectivity measured in group ROIs are provided in the Supplementary Materials.

Relatedly, what was the rational behind defining the language ROIs in an entirely different way from the ToM ROIs and based on coordinates of one specific study?

The ToM and language analyses were originally planned by two different scientists for (potentially) two different projects – one on responses in ToM brain regions, and one on responses in language brain regions. The different choices for ROI definition were not the result of researcher degrees of freedom; both choices were detailed in the respective pre-registered analysis plans (ToM: https://osf.io/kyu3f/?view_only=35949e8028f743bc973932fe3adf7831; Language: https://osf.io/8cmt4/?view_only=fd6a8bc69f1041ae8ec183ea8221771b). We ultimately decided to publish the results together in a single manuscript because we believe the language results strengthen the interpretation of the ToM results. However, because the main argument of this paper is about the behavioral and neural development of ToM, we have moved the details of the language analyses and results to the Supplementary Information. We still include a brief description of the language results in the main text and Table 2. We additionally conduct analyses of both networks in group regions of interest – which, for both networks, were created based on coordinates reported by prior studies. These results are reported in detail in the Supplementary Information.

Also, I believe reference 67 is not the correct reference here?

Thank you for catching this mistake. This reference has been corrected (the correct reference is:

> **Fedorenko, E., Hsieh, P. J., Nieto-Castañón, A., Whitfield-Gabrieli, S., & Kanwisher, N. (2010). New method for fMRI investigations of language: defining ROIs functionally in individual subjects. *Journal of neurophysiology*, *104*(2), 1177-1194.)**

I was surprised how many regions were included in the analysis of the language ROIs, especially given how restrictive the authors were with respect to the ToM network (i.e., only including rTPJ and DMPFC rather than the entire ToM network).

We include results from the entire ToM network in our Supplementary Materials (see a revised Figure S2, above), and reference these results in the Results section:

> **"While there were no significant age of ASL onset-by-ROI interactions (Table 2), suggesting that the size of the effect did not vary significantly across RTPJ and DMPC ROIs, post-hoc analyses within each ROI found a significant effect of age of ASL onset on selectivity in children's RTPJ only (effect in RTPJ: b=-.47, t=-2.3, 95% CI=[-.90,-.05], p=.03; effect in DMPFC: b=-.09, t=-.39, 95% CI=[-.58,.40], p=.7; all other ToM ROIs: all bs<|.27|, ts<|1.2|, 95% CIs=[(-.82,-.46),(.22,.39)], ps>.25; Table S3 and Figure S2)."**

We test all of the language regions described by Fedorenko et al., 2010 (except cerebellar ROIs, due to lack of coverage). We did not have a specific hypothesis for which language regions might be affected by delayed access to language, and wanted our analyses to be comprehensive.

8) How was ASL onset exactly defined to be included as a covariate in the regressions e.g. in Table 1? Were native and delayed signers treated separately? Or how did the authors take into account the skewed distribution of this variable?

ASL onset was a continuous variable referring to the age at which a child was exposed to ASL. For native signers, this value was 0. We used linear regressions for our analyses, which do not require dependent variables to be normally distributed. We additionally confirmed the significant effect of age of ASL onset on RTPJ selectivity by directly comparing native and delayed signers using a non-parametric Mann-Whitney U test:

> **"Non-parametric Mann-Whitney U tests confirmed that delayed signing children had less selective responses than native signing children in RTPJ (M(SE) selectivity in delayed signers: 25.5(8.9), native signers: 55.4(7.1), Z=-2.4, p=.02)."**

9) Role of inhibition?
- l. 256 ff.: The authors report that participants' response inhibition was assessed, but I was unable to find the results related to this measure. How was ASL onset related to response

inhibition? What role did inhibition play for the relation of ASL onset with ToM performance and with neural selectivity for mental state reasoning?

Thank you for pointing this out – we have added results from our behavioral measure of executive functions, which was a computerized flanker task made based on a prior paper (Rueda et al., 2004). We also now refer to this measure as "executive functions" rather than "response inhibition." Briefly, this measure was not significantly correlated with age of ASL onset, and did not differ between native and delayed signers. It additionally was not correlated with ToM behavioral performance or RTPJ selectivity. We also did not observe developmental improvement with age on this task. Overall, if there were individual differences in executive functions in this sample, this measure did not appear to be sensitive to them. We have added a brief description of these methods & results to the main text:

> **Methods:**
> **"Children also completed a standardized task of non-verbal IQ (KBIT-II (Kaufman, 1997)) and, when possible, a spatial working memory task (computerized CORSI (Kessels, Van Zandvoort, Postma, Kappelle, & De Haan, 2000; Vandierendonck, Kemps, Fastame, & Szmalec, 2004); n=24) and an executive functions task (custom-made computerized flanker task, modeled after a prior study (Rueda et al., 2004); n=24)."**
>
> **Results:**
> **"We additionally tested for differences in non-verbal IQ, spatial working memory, and executive functions as a function of age of ASL onset. Age of ASL onset was not correlated with spatial working memory span (M(SE) span in delayed signers: 4.9(.23); native signers: 4.7(.18); effect of age of ASL onset: b=-.27, t=-1.4, p=.18; effect of age: b=.61, t=3.2, p=.004) or executive functions (M(SE) reaction time cost (s) for incongruent vs. congruent flanker trials in delayed signers: .05(.02); native signers: .03(.02); effect of age of ASL onset: b=-.08, t=-.36, p=.72; effect of age: b=.31, t=1.4, p=.17)."**

10) Item analysis
It didn't get quite clear to me whether there was a main effect of item category in the exploratory regression in l.272 ff.? This would need to be the case in order to test whether children performed worse in one category than another.

We have tried to clarify this result:

> **"In a post-hoc linear regression testing for effects of age of ASL onset, age, item category ("easy", false beliefs, moral judgment, and "hard"), age of ASL onset had a significant negative effect on ToM_L performance (b=-.38, t=-4.7, p=8.0x10^{-6}), age had a significant positive effect on performance (b=.38, t=4.2, p=4.7x10^{-5}), and all children performed significantly worse on "hard" item category (relative to "easy": b=-.84, t=-3.6, p=.0004); there were no significant item category by age of ASL onset interactions (bs<|.27|, ts<|1.3|, ps>.2)."**

We believe that readers will be interested to view the results of the post-hoc analyses by item type, specifically because 1) isolating the false belief items enables comparison to most prior research on ToM development in this population, which focuses on false belief tasks, and 2) the moral reasoning items were the most advanced ToM items included in both task formats.

Reviewer #3 (Remarks to the Author):

This manuscript aims to investigate the documented role of language development in Theory of Mind (ToM). Currently there are two main views: one is that ToM tasks require some level of linguistic abilities and this is why there exists a relationship between the two domains. The second view is that language plays some role in the development of ToM concepts themselves. The authors here aimed to tease apart these two explanations by investigating the specificity of the response in the classic ToM brain regions on the premise that specificity is equivalent to functional maturity. In this sense, if a brain region 'for' a particular function is functionally mature, it responds to a narrow range of inputs, characteristic of its function. What they find here is that deaf children who are late signers show less specificity of the TPJ than early signers, and, since late signers are likely to have less language input than early signers, the inference is that language contributes to cortical specificity. I am generally positive about this paper and think it makes a nice contribution to a very tricky theoretical question. I follow their logic and though I am uncomfortable with the very small sample size upon which these analyses are based, I equally recognize that this sample is valuable. Thus, I do think that the results are likely to be an important contribution to the theoretical debate.

Thank you very much for your thoughtful summary and general encouragement.

That said, I have some comments and questions that I think the authors should deal with.

1. I found it difficult to figure out what they think is the relationship between cortical specialization and performance on behavioural tasks. They report that response selectivity did not correlate with ToM behavior and I am wondering why it doesn't? Late signing deaf kids do worse in ToM tasks, including 'minimal linguistic' tasks. Maybe the sample size is too small to find a relationship but then what is the role of this reduced specificity? The authors don't really talk about why they think that reduced selectivity would lead to worse performance (and even though they didn't find that relationship in this paper, the implication is that if language is important in the development of ToM, this immature selectivity is a factor).

This is a good question and we have incorporated the following edit to make the relevant hypotheses and interpretations clear.

> **"In many cortical regions, increasing selectivity during childhood occurs by the suppression of responses to non-preferred stimuli. For example, selectivity of the fusiform face area develops through the suppression of responses to non-face objects; this suppression correlates with performance on face recognition tasks (Cantlon et al., 2010; Golarai et al., 2007; Gomez et al., 2017). Selectivity of the**

> **visual word form area develops through the suppression of responses to faces (Cantlon et al., 2010); this suppression predicts literacy and reading expertise (Dehaene et al., 2010; Dehaene-Lambertz et al., 2018). Similarly, selectivity of the RTPJ develops through the suppression of responses to other (non-mentalistic) social information, and correlates with performance on ToM tasks (Gweon et al., 2012). The development of a spatially localized neural population with shared selectivity for mental state reasoning may facilitate finer-grained distinctions between mental state concepts (Kanwisher, 2010; Koster-Hale & Saxe, 2013). While the development of selective brain regions for perception of faces or words appears to depend on extensive experience with those visual categories (Arcaro et al., 2017; Dehaene et al., 2010; 2015), there is currently no evidence concerning the degree to which development of selective responses in RTPJ is influenced by environmental input, nor about which aspects of the environment drive development of selective responses in this region."**

It is difficult to interpret the lack of correlation between RTPJ selectivity and ToM performance in the current experiment and sample. It may be that we did not have the power to detect this correlation, given our small sample. While RTPJ selectivity and ToM performance are not correlated in this sample, we find it encouraging that they are the two measures (out of many) that were significantly impacted by delayed access to language.

In order to help bolster confidence in the selectivity measure, we include results from analyses of a small pilot dataset that suggests that RTPJ response selectivity is a reliable measure within individual participants (Supplementary Information & Figure S6).

2. The authors found no relationship in adults. Do we know that late signers eventually catch up such that no remnants of their impairment can be observed? If I remember correctly, Gary Morgan found that adults who learned to sign after the age of 10 years, showed lasting deficits in Theory of Mind use. Would then the hypothesis be that these very late signers would continue to show reduced selectivity into adulthood?

This is indeed a plausible hypothesis concerning the development of selective responses in late signers. All of our delayed signing child participants, and the vast majority of our delayed signing adult participants, were "early signers" – who received access to ASL after a delay ranging from .25 – 7 years (and before the 10 year cut-off described above). Thus our sample is not suited for testing hypotheses about late access (> 10 years) to ASL.

Another possibility is that magnitude of selective responses is not a sensitive neural marker of individual differences in ToM, once ToM reaches a certain point of maturity. That is, this neural measure might not be sensitive to subtle or later-developing aspects of ToM.

We have addressed this response in the following section in Discussion:

> **"All of our child participants, and a vast majority of our adult participants, received access to ASL after a relatively short delay (.25 – 7 years) – suggesting that even relatively short delays can delay the development of selective responses for ToM in**

> **RTPJ. From our results alone, it is unclear how much linguistic experience is sufficient to overcome this delay, or if there is a sensitive period for the impact of linguistic experience on ToM. Prior behavioral studies have found evidence for enduring ToM delays in adults who received access to language after longer delays (e.g., after age ten years; Gagne & Coppola, 2017; Morgan & Kegl, 2006; O'Reilly et al., 2014), but there is also evidence that ToM delays can resolve with subsequent linguistic experience (Morgan & Kegl, 2006; Peterson et al., 2005; 2012; Pyers & Senghas, 2009). Here, we did not observe an enduring effect of delayed access to language on RTPJ selectivity in adults. Additional work is needed to determine whether there are prolonged effects of delayed access to language on more sophisticated aspects of ToM reasoning (O'Reilly et al., 2014), and/or more fine-grained aspects of the neural response (e.g., the organization of spatial response patterns within RTPJ)."**

3. How do these data mesh with the apparently quite narrow selectivity of the TPJ region in infants (Hyde et al., 2018). In that study, the authors found that the TPJ was particularly responsive to a 'false belief' event and less responsive (if I remember correctly, not responsive above chance) to a 'true belief' or 'false belief that doesn't matter' event. This would seem to imply quite a lot of specificity is already there in very young infants – despite presumably no language comprehension, at least not of mental state terms. I don't know what the answer is, but I do think the authors should discuss this in their paper. I think the implication of the current manuscript should be that whatever infants are doing on infant-false-belief tasks, it isn't thinking about nuanced mental states like beliefs, which (according to the current data) would presumably be expected to be language-dependent.

Thank you for raising this point. This debate is certainly relevant to the arguments in this paper, and we include it as part of the theoretical motivation for the current experiment in the Introduction:

> **"… in a single testing session young children can fail linguistic false belief tasks and succeed on conceptually analogous non- or minimally linguistic false belief tasks (Clements & Perner, 1994; Geren et al., 2009; see also Wang et al., 2016), which reduce linguistic task demands by showing the narrative through sequences of pictures or movies, and by measuring action predictions via eye gaze or pointing behaviors. Even very young children show precocious success on non- or minimally linguistic false belief tasks (He et al., 2011; Onishi & Baillargeon, 2005; Scott et al., 2012), suggesting that children's linguistic abilities may be a rate-limiting factor for performance on traditional ToM tasks.**
>
> **One possibility is therefore that linguistic *experience* primarily affects performance on ToM tasks indirectly, via a direct effect on the linguistic *abilities* that children need for most ToM tasks. This possibility is particularly salient given the controversial evidence that toddlers and even infants pass false belief tasks when presented in non-linguistic formats (Onishi & Baillargeon, 2005; Southgate et al., 2007; Buttelmann et al., 2009; though see Barone et al., 2019; Kulke & Rakoczy, 2018; Powell et al., 2017). Some have claimed that human infants have the**

> conceptual repertoire and representational capacities to reason about others' beliefs, and that subsequent behavioral improvement on traditional ToM tasks primarily reflects development of domain-general abilities, such as language and executive functions that enable children to meet task demands (Carruthers, 2013; Scott & Baillargeon, 2017). However, the representational capacities that are required to pass non-linguistic false belief tasks remain debated (Heyes, 2014; Southgate, 2018).
>
> On the other hand, conversational experience may play a direct role in the construction of ToM concepts and representations."

There are at least two possible ways that our results may be integrated with Hyde's. The first possibility is that infants do have some basic capacity to represent false beliefs, sufficient for an action prediction task (and sufficient to recruit RTPJ responses), but which nevertheless undergoes substantial refinement or development, following linguistic experiences.

The alternative hypothesis, which the reviewer favors (as do we), is that infants can predict actions in some simple false belief scenarios without having a mature (or fully meta-representational) concept of beliefs. If so, the RTPJ may play a role in both the early precursors of ToM (whatever infants use) as well as the richer, linguistically mediated explicit ToM that develops over the course of childhood.

Indeed, a number of recent studies have argued that the role of the RTPJ in ToM is continuous over development, even while the child's behavioral performance, on ToM tasks generally and on false belief tasks specifically, undergoes substantial and dramatic change (Richardson et al., 2018; Bowman et al., 2019).

While a full discussion of these issues is out of the scope of the current manuscript (especially given feedback to simplify the manuscript), we have added the following text to the Discussion section:

> "A classic theoretical debate about social cognitive development posits that ToM is either instantiated in a distinct domain-specific biological mechanism, or is constructed through conversational interactions and social relationships (Carlson & Moses, 2001; Hughes & Devine, 2015; Scholl & Leslie, 2001). By contrast, these results suggest that ToM may be all of the above: RTPJ appears to be a domain-specific biological mechanism, in which selectivity is constructed in part through language exposure. One recent version of this theoretical debate concerns the nature of early success on non-linguistic (implicit) false belief tasks in toddlers and infants (Onishi & Baillargeon, 2005; Southgate et al., 2007; Buttelmann et al., 2009; though see Barone et al., 2019; Kulke & Rakoczy, 2018; Powell et al., 2017). A recent study using functional near-infrared spectroscopy (fNIRS) found evidence for activation in RTPJ to false belief scenarios in 7-month old infants (Hyde, Simon, Ting, & Nikolaeva, 2018). How can RTPJ support performance on such tasks, given our evidence that RTPJ development depends on rich linguistic experience? Of course, this question requires additional

research. But one possible explanation is that RTPJ is already selective for early-developing ToM concepts (e.g., reasoning about goals, perceptions, and knowledge access), and that linguistic experience shapes later-developing ToM concepts (e.g., belief representation, pragmatics) within a single continuous neural system."

Reviewers' comments:

Reviewer #1 (Remarks to the Author):

The authors have done a reasonable job responding to the reviews. I remain uncomfortable with the narrow (and modular) focus on theory of mind without any framing of other issues that might help indicate the relevance of early language deprivation more broadly. In particular, given that the authors only now report that their executive function measure did not show any effects of language delay, they really should include acknowledgement that other researchers have found language delay to have broad cognitive effects. Hall et al.'s recent JSLHR paper on executive function is important information for people concerned about this issue to be aware of. Such a discussion may well be something for the supplementary material, but it is important to acknowledge the breadth of cognitive processes potentially affected by language delay. As it stands, the findings here that people with delayed exposure to language "catch up" on theory of mind abilities is something that other researchers, such as those looking at implicit learning, executive function, and other cognitive domains, would be interested in. It would help to broaden the conversation by acknowledging other work in the general domain.

Reviewer #2 (Remarks to the Author):

Most of my comments and concerns have been addressed. Three requests remain that I feel need to be addressed so that the main message of the paper is accurate and the conclusions are fully supported:

1) The study reports a negative correlation of ASL-onset with the neural selectivity in the rTPJ for mental states rather than general social content. The authors argue that this reflects delayed ToM processing due to delayed access to language. While I agree with the authors that this is a plausible explanation for their findings, the arguments against the following alternative explanation still need to be refined: Children with less sophisticated language abilities might have had difficulties to differentiate mental from social contents because of linguistic difficulties, especially in the case of the most complex ToM concepts (such as, humor and second-order ToM).

The strongest argument here is that the reduced selectivity in delayed signers resulted from high neural activity in the social rather than reduced activity in the mental condition. As far as I can see, the authors have not directly tested for this statistically. I would suggest to make this prediction more explicit and then directly test for it statistically.

(I am less convinced of the additional arguments made by the authors: High ASL proficiency as tested by the ASL-RST might not have covered linguistic aspects required for the complex ToM items (e.g., second order embedding or sarcasm/reading between the lines). Similarly, I am not sure how the selectivity of language regions directly speaks to the children's ability to understand specific ToM items.)

2) The authors have moved a number of results to the Supplemental Information. While I see how this was necessary given the complex array of findings, I feel that the complexity of the results is not well captured by the main article anymore. For reasons of transparency, I would suggest to add the following 2 findings to the Abstract and Discussion of the paper:

- The RTPJ shows a decreased selectivity in delayed signers. However, the other regions of the ToM network (in particular, the LTPJ) show a selectivity for mental versus social contents also in delayed signers (see Figure S2). This should be mentioned and discussed in the main article.

- Delayed signers had similar patterns of activation and functional maturity of the RTPJ in the non-

verbal movie viewing task. The discussion of this finding should be added to the main paper, and this should be mentioned briefly in the Abstract.

3) Given that the data was collected up to 8 years ago, it makes sense that the analyses were preregistered after data collection. Nevertheless, for reasons of transparency, I would suggest to mention this in the manuscript along with the reference to the preregistration.

Reviewer #3 (Remarks to the Author):

The authors have answered the concerns/comments that I had and I have no further comments. I recommend publication of the manuscript.

Victoria Southgate.

Reduced Neural Selectivity for Mental States in Deaf Children with Delayed Exposure to Sign Language

Tracking number: NCOMMS-19-19645A

Response to Reviewers

We would like to thank the reviewers for their insightful feedback. We once again feel that their comments and suggestions have improved our manuscript. We provide point-by-point responses to their feedback below using bold font. Edited portions of the manuscript text are highlighted.

Reviewers' comments:

Reviewer #1 (Remarks to the Author):
The authors have done a reasonable job responding to the reviews. I remain uncomfortable with the narrow (and modular) focus on theory of mind without any framing of other issues that might help indicate the relevance of early language deprivation more broadly. In particular, given that the authors only now report that their executive function measure did not show any effects of language delay, they really should include acknowledgement that other researchers have found language delay to have broad cognitive effects. Hall et al.'s recent JSLHR paper on executive function is important information for people concerned about this issue to be aware of. Such a discussion may well be something for the supplementary material, but it is important to acknowledge the breadth of cognitive processes potentially affected by language delay. As it stands, the findings here that people with delayed exposure to language "catch up" on theory of mind abilities is something that other researchers, such as those looking at implicit learning, executive function, and other cognitive domains, would be interested in. It would help to broaden the conversation by acknowledging other work in the general domain.

We appreciate this feedback. We have edited the following paragraph in the Discussion section in order to address this comment:

> **"In contrast to our results, though, prior studies find that delayed access to language has broad cognitive effects (Hall, 2017; Humphries et al., 2016), including delayed development of language comprehension and production (Newport, 1990; Mayberry & Eichen, 1991; Henner, 2016; Mayberry & Kluender, 2018), literacy (Mayberry et al., 2011; Lederburg et al., 2013), and executive functions (Hall et al., 2018), differences in language-related brain development (Mayberry et al., 2011; Skotara et al., 2012; Penicaud et al., 2013), and consequences for mental health (Hall et al., 2017; Anderson et al,. 2016). This discrepancy is likely related to characteristics of our sample. All of our child participants, and a vast majority of our adult participants, received access to ASL after a relatively short delay (.25 – 7 years), and participants were screened to confirm that they had sufficient ASL proficiency to complete the study tasks. Broader cognitive and emotional effects of language delay may be strongest following longer delays that more substantially impede ASL acquisition.**

While our results suggest that even relatively short delays can delay the development of selective responses for ToM in RTPJ, from our results alone, it is unclear how much linguistic experience is sufficient to overcome this delay or if there is a sensitive period for the impact of linguistic experience on ToM. Prior behavioral studies have found evidence for enduring ToM delays in adults who received access to language after longer delays (e.g., after age ten years; Gagne & Coppola, 2017; Morgan & Kegl, 2006; O'Reilly, Peterson, & Wellman, 2014), but there is also evidence that ToM delays can resolve with subsequent linguistic experience (Morgan & Kegl, 2006; Peterson, Wellman, & Liu, 2005; Peterson, Wellman, & Slaughter, 2012; Pyers & Senghas, 2009). Here, we did not observe an enduring effect of delayed access to language on RTPJ selectivity in adults. Additional work is needed to determine whether there are prolonged effects of delayed access to language on more sophisticated aspects of ToM reasoning (O'Reilly et al., 2014), and/or more fine-grained aspects of the neural response (e.g., the organization of spatial response patterns within RTPJ)."

We have also added the Hall reference to the paragraph below:

"Future research is also necessary to determine whether other factors plausibly related to linguistic experience and ToM development (e.g., presence of siblings (Woolf et al., 2003), peer relationships (Peterson et al., 2016), executive functions (Hall et al., 2018) mediate the correlation between these two variables, to test which aspects of linguistic experience (e.g., mental state vocabulary (Hale & Tager-Flusberg, 2003), syntactic complement structures (de Villiers & de Villiers, 2009; de Villiers & Pyers, 2002; Hale & Tager-Flusberg, 2003; Lohmann & Tomasello, 2003; Ruffman, Slade, & Crowe, 2002), conversational turns (Morgan et al., 2014)) promote RTPJ selectivity (Bianco, Lecce, & Banerjee, 2016; Lecce, Bianco, Demicheli, & Cavallini, 2014a; Lecce, Bianco, Devine, Hughes, & Banerjee, 2014b; Lohmann & Tomasello, 2003), and to characterize whether and which other aspects of experience influence RTPJ development (Hughes & Devine, 2016). FNIRS may be particularly suited for future studies with deaf children, given the importance of replicating these results in a larger sample of participants and the ability to collect fNIRS data in individuals who have metallic cochlear implants."

Reviewer #2 (Remarks to the Author):

Most of my comments and concerns have been addressed. Three requests remain that I feel need to be addressed so that the main message of the paper is accurate and the conclusions are fully supported:

1) The study reports a negative correlation of ASL-onset with the neural selectivity in the rTPJ for mental states rather than general social content. The authors argue that this reflects delayed ToM processing due to delayed access to language. While I agree with the authors that this is a plausible explanation for their findings, the arguments against the following alternative explanation still need to be refined: Children with less sophisticated language abilities might

have had difficulties to differentiate mental from social contents because of linguistic difficulties, especially in the case of the most complex ToM concepts (such as, humor and second-order ToM).

Thank you for this feedback. The defining characteristic of the story stimuli in the Mental condition is that they have explicit mental state vocabulary (i.e., words that refer to beliefs, desires, and emotions, e.g., "thought", "wanted", "felt"). The mental state verbs included in the Mental stories are relatively simple and easy to understand – as the stories were written for children. The Mental stories do not require children to understand humor or sarcasm, or to "read between the lines." (We tested children's understanding of these more complex ToM concepts behaviorally, only).

As for second-order ToM: both mental and social stories include reported speech/action, which is sometimes called "role shift" in literature on ASL. An English equivalent of "role shift" would be something like, "She was all, 'He doesn't know where the gold is.'" Across different languages, there continues to be debate as to whether quoted speech/action is syntactically embedded under the sentence that introduces it. Given this, determining whether there are second-order/embedded mental states in our ASL stimuli is somewhat complicated. However, while all of the mental stories contain multiple mental state verbs and role shifts, only one story included a possible example of an embedded mental state, and instances of role shifts were equally frequent across Mental & Social conditions.

We suspect that the reviewer was misled to believe that the Mental state story stimuli are more complex than they actually are because the Mental state story example initially included in the Discussion section was pulled from the English version of this task, and it included an embedded mental state. We have updated this example such that it comes from the ASL experiment and is representative of the ASL stimuli:

> **"Here, in native signing children and all adults, the RTPJ showed selective responses to stories that described mental states (Mental condition; "The pirate thought that a pile of gold was buried behind Jimmy's house"), with low responses to Social stories that described people's physical appearance or enduring relationships, but not their mental states (Social condition; "Sarah and Lori play together on the soccer team")."**

We have also added edited the stimuli description in the Methods section, which additionally includes a link to the text "glosses" of all ASL stimuli:

> **"During the fMRI scan, participants watched movies of a woman telling stories in ASL, which involved characters and their mental states (Mental condition), characters and their physical appearance or enduring social relationships (Social condition), or descriptions of physical objects and events in the world (Physical condition). Mental stories included relatively simple mental state verbs ("know", "want", "surprised") and did not evoke reasoning about humor, sarcasm, or pragmatics. A subset of the stimuli (24/42 stories) were directly translated from English stories previously used to measure neural responses in hearing children and**

adults (Gweon, Dodell-Feder, Bedny, & Saxe, 2012; Saxe, Whitfield-Gabrieli, Scholz, & Pelphrey, 2009); the remaining stimuli were similar in style (see https://osf.io/kyu3f/?view_only=35949e8028f743bc973932fe3adf7831 for all stimuli). All 42 stories were rated for linguistic features (syntactic complexity, number of signs, number of verbs, and, for Mental and Social stories, number of role shifts), psychological features (how easy to understand, how interesting), and imageability by 10 Deaf native signing adults who were naïve to the experimental hypotheses. These ratings were used to ensure that stimuli were matched across conditions. Stories were told using simple language, in an enthusiastic, narrative way."

The strongest argument here is that the reduced selectivity in delayed signers resulted from high neural activity in the social rather than reduced activity in the mental condition. As far as I can see, the authors have not directly tested for this statistically. I would suggest to make this prediction more explicit and then directly test for it statistically.

(I am less convinced of the additional arguments made by the authors: High ASL proficiency as tested by the ASL-RST might not have covered linguistic aspects required for the complex ToM items (e.g., second order embedding or sarcasm/reading between the lines). Similarly, I am not sure how the selectivity of language regions directly speaks to the children's ability to understand specific ToM items.)

Thank you; we have added a simple and direct test of this hypothesis to the Supplementary Information (given the post-hoc/exploratory nature of the test). We tested whether the beta estimates for Mental and Social in the group RTPJ ROI correlate with age of ASL onset – predicting an effect of age of ASL onset on the size of the beta estimate for Social, but not Mental, stories. We found evidence consistent with our prediction:

"In a post-hoc exploratory analysis, age of ASL onset correlated with the magnitude of response (beta estimate) for the Social condition ($r_s(22)=.57$, 95% CI=[.22,.90], p=.004), but not for the Mental condition ($r_s(22)=.09$, 95% CI=[.36,.55], p=.66). That is, children who experienced longer delays had higher RTPJ responses to the Social stories."

We have also edited the relevant section of the Discussion for clarity:

"In our view, reduced selectivity of RTPJ reflects differential development of ToM. An alternative hypothesis that we do not favor is that delayed access to language impeded children's comprehension of the mental state stories. The pattern of results is not consistent with this alternative. First, all children showed high RTPJ responses to the Mental stories, suggesting that the semantic content of these stories was successfully extracted. The difference in selectivity following delayed access to sign language reflects a *high* response to Social stories, not a low response to Mental stories (see Supplementary Information). Second, we did not observe an effect of delayed access to ASL on the development of selective responses in language-related regions, nor did we observe additional recruitment of brain regions that would indicate greater difficulty processing the linguistic stimuli in delayed signers (e.g.,

> **language brain regions or the multiple demand network). However, these are null results and so should be interpreted with caution. Finally, children in both groups performed well on first-order false belief tasks presented in ASL. All in all, we suggest that all children could comprehend the Mental state stories, and differences in response to the Social stories reflects a delayed developmental process of increasing response selectivity by suppressing neural responses to dis-preferred stimulus categories. "**

2) The authors have moved a number of results to the Supplemental Information. While I see how this was necessary given the complex array of findings, I feel that the complexity of the results is not well captured by the main article anymore. For reasons of transparency, I would suggest to add the following 2 findings to the Abstract and Discussion of the paper:

- The RTPJ shows a decreased selectivity in delayed signers. However, the other regions of the ToM network (in particular, the LTPJ) show a selectivity for mental versus social contents also in delayed signers (see Figure S2). This should be mentioned and discussed in the main article.

We have added the following sentence to the Results section:

> **"Exploratory analyses of other ToM brain regions (left TPJ, precuneus, mid- and ventro-medial prefrontal cortex) did not find evidence for delayed development as a function of delayed access to language (all bs<|.27|, ts<|1.2|, 95% CIs=[(-.82,-.46),(.22,.39)], ps>.25; Figure S2, Table S3)."**

And to the Discussion section:

> **"Consistent with this prediction, the RTPJ of delayed signing children showed high responses to *both* Mental stories and Social stories. Moreover, the length of the delay prior to access to ASL correlated with reduced selectivity of RTPJ (despite current ASL proficiency and controlling for age). There was no effect of language delay on other "ToM" brain regions (LTPJ, precuneus, MPFC), which are recruited during ToM reasoning but are not as selective for mental states (i.e., LTPJ is recruited for meta-representation more generally (Perner et al., 2006), and MPFC for reasoning about other aspects of people (Mitchell et al., 2005)). Overall, our results suggest that linguistic experience has a direct impact on selective responses in RTPJ. Rather than resembling the response in age-matched children, who have the same amount of biological maturation, the response profile in RTPJ in delayed signing children resembles that previous observed in young children (Gweon et al., 2012; Saxe et al., 2009), who have a similar amount of linguistic experience."**

- Delayed signers had similar patterns of activation and functional maturity of the RTPJ in the non-verbal movie-viewing task. The discussion of this finding should be added to the main paper, and this should be mentioned briefly in the Abstract.

We agree that the movie-viewing experiment is an interesting component of our study. In an effort to simultaneously maintain a thorough discussion of these data and a coherent discussion of our primary experiment, we have opted to include two pages describing and discussing the movie-viewing experiment, results, and (potential) interpretation in the

Supplementary Information. We do not mean to obscure these results; we have received feedback from multiple reviewers that a Discussion section that includes both experiments is too long/complicated and, in the case of the movie-viewing experiment results, speculative. We have added additional text to the main manuscript to help ensure that this experiment and the results are not overlooked by our readers:

Results section:

> **"We measured neural responses during a non-linguistic movie-viewing experiment. We did not observe any differences in the neural response of ToM regions or in the RTPJ specifically to the movie as a function of age of ASL onset (among children, adults, or in the full sample); see Supplemental Information for methods, results, and discussion."**

In the Discussion section:

> **"A recent study using functional near-infrared spectroscopy (fNIRS) found evidence for activation in RTPJ to false belief scenarios in 7-month old infants (Hyde, Simon, Ting, & Nikolaeva, 2018). How can RTPJ support performance on such tasks, given our evidence that RTPJ development depends on rich linguistic experience? Of course, this question requires additional research. But one possible explanation is that RTPJ is already selective for early-developing ToM concepts (e.g., reasoning about goals, perceptions, and knowledge access), and that linguistic experience shapes later-developing ToM concepts (e.g., belief representation, pragmatics) within a single continuous neural system. This explanation is consistent with one account for the lack of differences between native and delayed signers' RTPJ response during the movie-viewing experiment (see Supplementary Information for further discussion) – but additional work is necessary to rigorously assess this account."**

Abstract:

> **"Language provides a rich source of information about other people's thoughts and feelings. Consequently, delayed access to language may influence conceptual development in Theory of Mind (ToM). We used functional magnetic resonance imaging and behavioral tasks to study ToM development in child (n=33, 4-12 years old) and adult (n=36) fluent signers of American Sign Language (ASL), and provide the first characterization of neural ToM responses during an ASL task and during a movie-viewing task. Participants included deaf children whose first exposure to ASL was delayed up to 7 years (n=12). Neural responses to ToM stories (specifically, selectivity of the right temporo-parietal junction) in these children resembled responses previously observed in young children, who have the same amount of linguistic experience, rather than those in age-matched native-signing children, who have the same amount of biological maturation. These results suggest that early linguistic experience facilitates ToM development, via the development of a selective brain region for ToM."**

3) Given that the data was collected up to 8 years ago, it makes sense that the analyses were

preregistered after data collection. Nevertheless, for reasons of transparency, I would suggest to mention this in the manuscript along with the reference to the preregistration.

We agree; thank you. We have added the following sentence to the description of the study in the Introduction:

> **"ASL story task analyses were pre-registered after data collection was completed."**

And have made the following edit to the Methods section:

> **"All analysis decisions (including preprocessing, region of interest selection and definition, motion exclusion and treatment, calculation of selectivity indices) and planned analyses for the story task were pre-registered via OSF after data collection was completed (ToM: https://osf.io/mhgp8; Language: https://osf.io/7y263)."**

We will also note for the reviewer that while we pre-registered our analyses after data collection was completed, our primary aims and hypotheses were described in the supporting grant application submitted in 2009 (NSF Career award (#1122374 to RS)).

Reviewer #3 (Remarks to the Author):
The authors have answered the concerns/comments that I had and I have no further comments. I recommend publication of the manuscript.

Victoria Southgate.

Thank you, Dr. Southgate. We appreciate your help in improving this manuscript!

REVIEWERS' COMMENTS:

Reviewer #2 (Remarks to the Author):

The authors have addressed all my remaining comments, I recommend the article for publication.

Response to Reviewers
Reduced Neural Selectivity for Mental States in Deaf Children with Delayed Exposure to Sign Language

Tracking number: NCOMMS-19-19645C

Reviewers' Comments
Reviewer #2: The authors have addressed all my remaining comments, I recommend the article for publication.

Thank you; we appreciate your feedback!
